# Supplementary material for: Ecological changes have driven biotic exchanges across the Indian Ocean
Source: Sci Rep. 2021 Dec 2;11:23357. doi: 10.1038/s41598-021-02799-7 (PMC8640032; doi:10.1038/s41598-021-02799-7)
Supplement: Supplementary file 1 — Supplementary Information. [file 41598_2021_2799_MOESM1_ESM.zip › S3.pdf]

Appendix 3: Coordinates used in ancestral range reconstruction

Table S20: Geographic coordinates for the regions as used in BayArea analyses.

Region	Latitude	Longitude
Africa	-1.2500	25.7225
Asia	39.7054	81.8409
Australia	-24.1385	133.3899
Comoros	-12.1837	44.0731
IAA	1.2500	113.4847
India	20.6835	79.1195
Madagascar	-19.4258	46.6878
Mascarene	-20.2856	57.5659
Seychelles	-4.7005	55.4899
America	10.7769	-79.1874
Pacific	-13.9791	-173.2304
New Zealand	-43.6889	171.00271
Europe	46.309	20.4464
Arabic Peninsula	22.2594	47.8352
Mediterranean	36.637	-2.9501
