# Supplementary material for: Ecological changes have driven biotic exchanges across the Indian Ocean
Source: Sci Rep. 2021 Dec 2;11:23357. doi: 10.1038/s41598-021-02799-7 (PMC8640032; doi:10.1038/s41598-021-02799-7)
Supplement: Supplementary file 1 — Supplementary Information. [file 41598_2021_2799_MOESM1_ESM.zip › S2.pdf]

Appendix 2: Specifics of the analyses

Please note that the analyses that followed the exact methodology described in the main text with the parameters supplied by the original authors list only the taxa available. Those analyses where we had to modify or add calibration points, or change priors to obtain chain convergence, have a short text explaining our modifications to allow replication of our results.

1. Acrocephalidae

Table S1: Genbank numbers for the sequences used in the Acrocephalidae analyses with the distribution attributed to each terminal.

Species	Locality	CytB	COI	ND2	LDH	ODC	Myo	FIB5	RAG1
Acrocephalus aedon	Asia	FJ883020 AJ004778	HQ608890	DQ125987	FJ883054	FJ883126	FJ883104		
Acrocephalus aequinoctialis	Pacific	EF156278		EF156314					
Acrocephalus agricola	Asia	FJ883021 AJ004775	KJ453121	KJ453197	FJ883055	FJ883127	FJ883108	KJ453263	KJ453456
Acrocephalus arundinaceus	Africa Asia Europe	FJ883022 AJ004784	FR847226	AB621334	FJ883056	FJ883128	FJ883098	KJ453264	KJ453460
Acrocephalus atyphus	Pacific	EF156297		HQ844387					
Acrocephalus australis	Australia	FJ883023 AJ004786	KJ453124	KJ453200	FJ883057	FJ883129	FJ883097	KJ453267	KJ453459
Acrocephalus baeticatus	Africa	KF547904	KJ453126	KJ453201	FJ883058	FJ883130	FJ883103	KJ453268	KJ453462
Acrocephalus bistrigiceps	Asia	FJ883025 AJ004766	KJ453127	KJ453203	FJ883059	FJ883131	FJ883109	KJ453270	KJ453464
Acrocephalus brevipennis	Africa	FJ883026	KJ453129	KJ453205	FJ883060	FJ883132	FJ883099	KJ453273	KJ453467
Acrocephalus caffer	Pacific	EF156308		EF156348					
Acrocephalus concinens	Asia	FJ883027	KJ453131	KJ453206	FJ883061	FJ883133	FJ883101	KJ453274	KJ453468
Acrocephalus dumetorum	Asia Europe India	FJ883028 AJ004773	KJ453133	KJ453208	FJ883062	FJ883134	FJ883105	EF626749	FJ358146
Acrocephalus familiaris	Pacific	EU119965		EU119962					
Acrocephalus gracilirostris	Africa	FJ883029 AJ004782	KJ453135	KJ453210	FJ883063	FJ883135	FJ883095	KJ453277	KJ453470
Acrocephalus griseldis	Africa	FJ883030 AJ004779	KJ453138	KJ453212	FJ883064	FJ883136	FJ883092	KJ453279	KJ453472
Acrocephalus kerearako	Pacific	EF156292		HQ844403					
Acrocephalus melanopogon	Europe Asia	FJ883032 AJ004767	KJ453139	KJ453214	FJ883066	FJ883138	FJ883093	KJ453281	KJ453474
Acrocephalus mendanae	Pacific	EF156310		EF156322					
Acrocephalus newtoni	Madagascar	FJ883033 AJ004780	KJ453141	KJ453216	FJ883067	FJ883139	FJ883123	KJ453283	AY319972
Acrocephalus orientalis	Asia	FJ883034 AJ004785	JF499074	KJ453218	FJ883068	FJ883140	FJ883102	KJ453285	KJ453477
Acrocephalus orinus	Asia	DQ681065							
Acrocephalus paludicola	Africa Asia Europe	FJ883035 AJ004768	KJ453144	KJ453220	FJ883069	FJ883141	FJ883112	KJ453287	KJ453479
Acrocephalus palustris	Africa Asia Europe	FJ883036 AJ004774	KJ453146	KJ453222	FJ883070	FJ883142	FJ883106	KJ453289	KJ453481
Acrocephalus percernis	Pacific	EF156298		HQ844418					
Acrocephalus rimitarae	Pacific	EF156306		EF156346					
Acrocephalus rufescens	Africa	FJ883037 Z73475	KJ453148	KJ453224	FJ883071	FJ883143	FJ883124	KJ453291	KJ453484
Acrocephalus schoenobaenus	Asia	FJ883038	KJ453150	KJ453226	FJ883072	FJ883144	FJ883107	KJ453293	KJ453486
Acrocephalus scirpaceus fuscus	Asia Africa	KF547898	KJ453154	KJ453230	KJ453349	KJ453433	KJ453390	KJ453300	KJ453488
Acrocephalus scirpaceus scirpaceus	Europe Africa	KF547878 Z73483	KJ453152	KJ453229	FJ883073	FJ883145	FJ883111	KJ453302	KJ453491

Acrocephalus sechellensis	Seychelles	FJ883040 AJ004781	KJ453156	KJ453232	FJ883074	FJ883146	FJ883122	KJ453295	KJ453492
Acrocephalus stentoreus	Arabic Peninsula	FJ883031 AJ004787	FJ465293	KJ453233	FJ883065	FJ883137	FJ883100	KJ453296	KJ453495
Acrocephalus taiti	Pacific	FJ883042 AJ004777	KJ453161	HQ844427	FJ883076	FJ883148	FJ883096		
Acrocephalus tangorum	Asia IAA	FJ883041	KJ453158	KJ453235	FJ883075	FJ883147	FJ883110	KJ453305	KJ453497
Acrocephalus vaughani	Pacific	KJ453185	KJ453161	KJ453237	KJ453354	KJ453440	KJ453394	KJ453299	KJ453493
Bradypterus baboecala	Africa	FJ883053		AY382344	FJ883090	FJ883162	DQ008525		JX236420
Calamonastides gracilirostris	Africa	FJ883043	KJ453162	KJ453239	FJ883077	FJ883149	FJ883113	KJ453306	KJ453499
Hippolais icterina	Europe Africa	FJ883046 DQ008479	KJ453164	GQ242091	FJ883078	FJ883153	FJ883120	EU680651	KJ453516
Hippolais languida	Africa Arabic Peninsula Asia	FJ883047 AJ004794	KJ453166	KJ453242	FJ883079	FJ883154	FJ883094	KJ453308	KJ453502
Hippolais olivetorum	Africa	FJ883048 AJ004795	JQ175054	KJ453243	FJ883080	FJ883155	FJ883121	KJ453310	KJ453503
Hippolais polyglotta	Africa Europe	FJ883050 AJ004797	KJ453169	KJ453245	FJ883081	FJ883157	FJ883115	KJ453312	KJ453505
Iduna caligata	Asia India	FJ883044 AJ004793	GQ481975	KJ453250	FJ883084	FJ883151	FJ883116	KJ453317	KJ453512
Iduna natalensis	Africa	DQ008523	KJ453171	KJ453252	FJ883082	FJ883150	FJ883114	KJ453319	KJ453519
Iduna opaca	Africa	FJ883049 AJ004790	KJ453173	KJ453253	FJ883086	FJ883156	FJ883118	KJ453320	KJ453513
Iduna pallida	Africa Asia	FJ883045 AJ004791	KJ453175	KJ453254	FJ883085	FJ883152	FJ883119	KJ453321	KJ453509
Iduna rama	Asia	FJ883051 AJ004792	KJ453176	KJ453256	FJ883087	FJ883158	FJ883117	KJ453323	KJ453514
Iduna similis	Africa	FJ899738	KJ453178	KJ453258	FJ883083	FJ883159	FJ883125	KJ453326	KJ453520
Locustella fluviatilis	Africa Arabic Peninsula Asia Europe	HQ608847	JQ175275	GQ242093	HQ706203	GQ242152	DQ008527	GQ242048	KJ453524
Locustella lanceolata	Asia IAA	DQ119525	GU571953	AY382379	FJ883088	FJ883160	FJ883091		
Megalurus palustris	IAA India	FJ883052	JF957022	JN614731	FJ883089	FJ883161	DQ008529	EU680661	AY319988
Nesilla typica	Madagascar	KJ453193	KJ453180	KJ453260	KJ453371	KJ453455	KJ453412	EU680665	KJ453522

For the analyses for the Acrocephalidae, we complemented some of the Cytochrome B sequences (see Table S1). We made two independent runs (see main text) and combined their results through LogCombiner (Drummond & Rambaut, 2007). Molecular rates were obtained from the original article.

2. Anatini

Table S2: Genbank numbers for the sequences used in the Acrocephalidae analyses with the distribution attributed to each terminal.

Species	Locality	CytB	12S	COI	ND2	ATP6
Aix sponsa	America	AF059053		AY666569	AF059114	
Amazonetta brasiliensis	America	AF059054	HM063533	JN801484	AF059115	
Anas acuta	Africa America Asia India	AF059055	HM063538	KBBI227-07	AF059116	
Anas americana	America	AF059103	HM063537	DQ433309	AF059163	
Anas aucklandica	New Zealand	AF059056	AF173482		AF059117	AF173490
Anas bahamensis bahamensis	America	AF059058		FJ027081	AF059119	
Anas bernieri	Madagascar	AF059060			AF059121	
Anas capensis	Africa	AF059105			AF059165	
Anas carolinensis	America	AF059063	HM063539	DQ434281	AF059123	
Anas castanea	Australia	AF059065	AF173481		AF059125	AF173494

Anas chlorotis	New Zealand	AF059061	AF173484		AF059122	AF173491
Anas clypeata	Africa America Asia India	AF059062	HM063536	BROMB439-06	AF059174	
Anas crecca	Africa Asia India	AF059064	HM063540	GU571238	AF059124	
Anas cyanoptera cyanoptera	America	AF059066	AF173690	BROMB795-07	AF059126	
Anas diazi	America	AF059069			AF059129	
Anas discors	America	AF059068	JF914878	AY666325	AF059128	
Anas erythrorhyncha	Africa	AF059070			AF059130	
Anas falcata	Asia	AF059106	AY164527	GQ481322	AF059166	
Anas flavirostris flavirostris	America	AF059071		JN801487	AF059131	
Anas formosa	Asia	NC_015482	NC_015482	NC_015482	NC_015482	NC_015482
Anas fulvigula	America	AF059074		DQ432723	AF059134	
Anas georgica spinicauda	America	AF059075		FJ027093	AF059135	
Anas gracilis	Australia	AF059076	AF173480		AF059136	AF173493
Anas hottentota	Africa Madagascar	AF059077	AF173692		AF059137	
Anas laysanensis	Pacific	AF059078		JF498830	AF059138	
Anas luzonica	IAA	AF059079			AF059139	
Anas melleri	Madagascar	AF059080			AF059140	
Anas nesiotis	New Zealand	AF059057	AF173483		AF059118	AF173489
Anas penelope	Africa Asia	AF059107	AY164518	GU571239	AF059167	
Anas platalea	America	AF059084		FJ027098	AF059144	
Anas platyrhynchos	Cosmopolite	NC_009684	NC_009684	NC_009684	NC_009684	NC_009684
Anas poecilorhyncha	Asia India	AF059083	AY164517	JF499090	AF059143	
Anas puna	America	AF059085		FJ027101	AF059145	
Anas querquedula	Africa Asia India	AF059086	AF173691	GQ481326	AF059146	
Anas rhynchotis rhynchotis	Australia	AF059087		BROMB529-07	AF059147	
Anas rubripes	America	AF059088		AY666211	AF059148	
Anas sibilatrix	America	AF059108		FJ027106	AF059168	
Anas smithii	Africa	AF059089			AF059149	
Anas sparsa	Africa	AF059091			AF059151	
Anas strepera	America	AF059109	AF173689	GQ481327	AF059169	
Anas superciliosa	Australia IAA Pacific	AF059092	AF173486	JN801396	AF059152	AF173488
Anas undulata	Africa	AF059093			AF059153	
Anas versicolor	America	AF059094		FJ027115	AF059154	
Anas zonorhyncha	Asia IAA	AF059095			AF059155	
Asarcornis scutulata	IAA	AF059099	HM063548		AF059159	
Aythya americana	America	NC_000877	NC_000877	NC_000877	NC_000877	NC_000877
Cairina moschata	America	NC_010965	NC_010965	NC_010965	NC_010965	NC_010965
Callonetta leucophrys	America	AF059097	HM063546	FJ027277	AF059157	
Chenonetta jubata	Australia	AF059100	HM063545	BROMB794-07	AF059160	
Cyanochen cyanopterus	Africa	AF059101	HM063550		AF059161	
Lophonetta specularioides	America	AF059102	HM063535		AF059162	
Marmaronetta angustirostris	Mediterranean	AF059104	HM063551		AF059164	
Pachyanas (Anas) chathamica	New Zealand	KF562761	KF562761	KF562761	KF562761	KF562761

Pteronetta hartlaubii	Africa	AF059110	HM063549		AF059170
Sarkidiornis melanotos	Africa IAA India Madagascar	AF059111	HM063541	FJ028237	AF059171
Speculanas specularis	America	AF059090	HM063534		AF059150
Tachyeres pteneres	America	AF059112	HM063532	JN802006	AF059172
Tadorna tadorna	Asia	AF059113	HM063544	GU571650	AF059173

The vouchers starting with BROMB are deposited in the Barcode of Life database.

For these analyses, we partitioned each position of the codon separately for all markers, except 12S. A prior to enforce the monophyly of the ingroup and the outgroup (*Aix*, *Cairina* and *Tadorna*) was also included. For the *A. soporata* calibration we placed the mean in real space instead of log space; this means that the mean age is the actual μ of a normal distribution and not the M of the lognormal distribution (the original article seems to have done that although it is not explicit in the Materials and Methods section). We made two independent runs and combined their results through LogCombiner.

3. Palaeognathae

Table S3: Genbank numbers for the sequences used in the Palaeognathae analyses with the distribution attributed to each terminal.

Species		Mitochondrion genome	BACH1	BMP2	CMOS	DNAH3	IRBP	NT3	PNN	PTPN	RAG2	TRAF6
Acanthisitta chloris		AY325307	JX533012	JX533043	JX533074	JX533122	JX533214	JX533265	JX533296	JX533327	JX533380	JX533411
Aepyornis hildebrandti	Madagascar	KJ749824										
Alectura lathami		NC_007227										
Alligator mississippiensis		NC_001922	JX533010	JX533041	JX533072	JX533120	JX533212	JX533263	JX533294	JX533325	JX533378	JX533409
Anas platyrhynchos		NC_009684										
Anomalopteryx didiformis	New Zealand	NC_002779	JX533038	JX533069	JX533100	JX533148	JX533240	JX533291	JX533322	JX533353	JX533406	JX533437
Anser albifrons		NC_004539	JX533023	JX533054	JX533085	JX533133	JX533225	JX533276	JX533307	JX533338	JX533391	JX533422
Anseranas semipalmata		NC_005933	JX533024	JX533055	JX533086	JX533134	JX533226	JX533277	JX533308	JX533339	JX533392	JX533423
Apteryx haastii	New Zealand	NC_002782										
Apteryx mantelli	New Zealand	AY016010	JX533033	JX533064	JX533095	JX533143	JX533235	JX533286	JX533317	JX533348	JX533401	JX533432
Apteryx owenii	New Zealand	NC_013806	JX533031	JX533062	JX533093	JX533141	JX533233	JX533284	JX533315	JX533346	JX533399	JX533430
Apus apus		NC_008540	JX533021	JX533052	JX533083	JX533131	JX533223	JX533274	JX533305	JX533336	JX533389	JX533420
Archilochus colubris		NC_010094	JX533022	JX533053	JX533084	JX533132	JX533224	JX533275	JX533306	JX533337	JX533390	JX533421
Arenaria interpres		NC_003712	JX533019	JX533050	JX533081	JX533129	JX533221	JX533272	JX533303	JX533334	JX533387	JX533418
Caiman crocodilus		NC_002744	JX533011	JX533042	JX533073	JX533121	JX533213	JX533264	JX533295	JX533326	JX533379	JX533410
Casuarius bennetti	Australia IAA	AY016011										
Casuarius casuarius	Australia	AF338713	JX533036	JX533067	JX533098	JX533146	JX533238	JX533289	JX533320	JX533351	JX533404	JX533435
Ciconia ciconia		NC_002197	JX533016	JX533047	JX533078	JX533126	JX533218	JX533269	JX533300	JX533331	JX533384	JX533415
Crypturellus tataupa	America	AY016012										
Dinornis giganteus	New Zealand	NC_002672										
Dromaius novaehollandiae	Australia	AF338711	JX533037	JX533068	JX533099	JX533147	JX533239	JX533290	JX533321	JX533352	JX533405	JX533436
Emeus crassus	New Zealand	NC_002673										
Eudromia elegans	America	NC_002772	JX533025	JX533056	JX533087	JX533135	JX533227	JX533278	JX533309	JX533340	JX533393	JX533424

Gallus gallus		NC_001323										
Gavia stellata		NC_007007	JX533013	JX533044	JX533075	JX533123	JX533215	JX533266	JX533297	JX533328	JX533381	JX533412
Haematopus ater		NC_003713	JX533018	JX533049	JX533080	JX533128	JX533220	JX533271	JX533302	JX533333	JX533386	JX533417
Mullerornis agilis	Madagascar	KJ749825										
Pterocnemia pennata	America	NC_002783	JX533030	JX533061	JX533092	JX533140	JX533232	JX533283	JX533314	JX533345	JX533398	JX533429
Pterodroma brevirostris		NC_007174	JX533017	JX533048	JX533079	JX533127	JX533219	JX533270	JX533301	JX533332	JX533385	JX533416
Pygoscelis adeliae		NC_021137	JX533015	JX533046	JX533077	JX533125	JX533217	JX533268	JX533299	JX533330	JX533383	JX533414
Rhea americana	America	NC_000846	JX533029	JX533060	JX533091	JX533139	JX533231	JX533282	JX533313	JX533344	JX533397	JX533428
Struthio camelus	Africa	NC_002785	JX533028	JX533059	JX533090	JX533138	JX533230	JX533281	JX533312	JX533343	JX533396	JX533427
Taeniopygia guttata		NC_007897										
Tinamus major	America	AF338707	JX533026	JX533057	JX533088	JX533136	JX533228	JX533279	JX533310	JX533341	JX533394	JX533425

In this analysis, the complete dataset and the mitogenome had a difference in topology: the mitogenome datset retrieved the same groups as the original article, but with all markers the rheas were retrieved as a sister-group to the *Aepyornis-Apteryx-Casuarius-Dromaius* clade. These two topologies, however, did not interfere with the obtained dates or with the RASP results and our further discussion.

4. Psittacoidea

Table S4: Genbank numbers for the sequences used in the Psittacoidea analyses with the distribution attributed to each terminal.

Species	Locality	CytB	CMOS	RAG1	ZENK
Ara macao	America	MH400248	JF807951	JF807979	JF807965
Agapornis canus	Madagascar	AF001332	GQ505083	GQ505191	GQ505138
Agapornis fischeri	Africa	AF346358	GQ505084	GQ505192	GQ505139
Agapornis lilianae	Africa	AF001331	GQ505087		
Agapornis nigrigenis	Africa	AF001328	GQ505085	GQ505193	GQ505140
Agapornis roseicollis	Africa	AF001330	GQ505086	GQ505194	GQ505141
Alisterus chloropterus	Australia IAA		GQ505091	GQ505199	GQ505145
Alisterus scapularis	Australia	FJ499014	GQ505090	GQ505198	GQ505144
Aprosmictus jonquillaceus	Australia	AB177959	GQ505092	GQ505200	GQ505146
Amazona aestiva	America	NC_033336	JF807952	JF807980	JF807966
Amazona dufresniana	America	AY283454	JF807953	JF807981	JF807967
Amazona pretrei	America		JF807954	JF807982	JF807968
Barnardius zonarius	Australia	JQ066207	GQ505095	GQ505203	GQ505149
Bolbopsittacus lunulatus	IAA		JF807955	JF807983	JF807969
Cacatua galerita fitzroyi	Australia IAA	AF313755	GQ505120	GQ505231	GQ505173
Cacatua moluccensis	IAA	AB177980	GQ505121	GQ505232	GQ505174
Calyptorhynchus funereus	Australia	JF414306	GQ505118	GQ505229	
Calyptorhynchus latirostris	Australia	JF414302	GQ505119	GQ505230	GQ505172
Charmosyna pulchella	Australia IAA	JX442429	GQ505126	GQ505237	GQ505179
Coracopsis nigra	Comoros Madagascar Seychelles	GQ996494	GQ505114	GQ505224	

Coracopsis vasa	Comoros Madagascar	AF346355	GQ505113	GQ505223	GQ505167
Cyanoramphus auriceps	New Zealand	JX442418	GQ505104	GQ505213	GQ505158
Cyanoramphus novaezelandiae	New Zealand	AF346380	GQ505103	GQ505212	GQ505157
Cyclopsitta diophthalma	Australia IAA		GQ505130		
Deroptyus accipitrinus	America	DQ150992	JF807956	JF807984	JF807970
Eclectus roratus	Australia IAA	AB177965	GQ505135	GQ505244	GQ505187
Eos cyanogenia	IAA	AF346330	GQ505122	GQ505233	GQ505175
Eunymphicus cornutus	Pacific	AF242516	GQ505106	GQ505215	GQ505159
Eunymphicus uvaeensis	Pacific	AF242514	GQ505107	GQ505216	GQ505160
Guarouba guarouba	America	DQ150990	JF807957	JF807985	JF807971
Lathamus discolor	Australia	JX442416	GQ505102	GQ505211	GQ505156
Loriculus catamene	IAA		GQ505088	GQ505195	GQ505142
Loriculus galgulus	IAA	AB177967	GQ505089	GQ505196	
Loriculus philippensis	IAA			GQ505197	GQ505143
Lorius garrulus	IAA	AB177951	GQ505125	GQ505236	GQ505178
Mascarinus mascarinus	Reunion	GQ996499			
Melopsittacus undulatus	Australia	EF450826		GQ505222	GQ505166
Micropsitta finschii tristrami	Pacific	U89176	GQ505128	GQ505240	GQ505182
Micropsitta pusio	Pacific		GQ505129	GQ505241	GQ505183
Neophema chrysogaster	Australia	NC_019804	GQ505110	GQ505219	GQ505163
Neophema chrysostoma	Australia	JX442425	GQ505111	GQ505220	GQ505164
Neophema pulchella	Australia	JX442424	GQ505109	GQ505218	GQ505162
Neophema splendida	Australia	JQ066212	GQ505108	GQ505217	GQ505161
Neopsephotos bourkii	Australia	JQ066213	GQ505112	GQ505221	GQ505165
Nestor notabilis	New Zealand	KX369037	JF807958	GQ505238	GQ505180
Northiella haematogaster	Australia	JX442432	JF807959	JF807986	JF807972
Pionus menstruus	America	EF517605	JF807960	JF807987	JF807973
Platycercus caledonicus	Australia	JQ066208	GQ505097	GQ505205	GQ505151
Platycercus eximius	Australia	DQ467901		GQ505206	GQ505152
Platycercus flaveolus	Australia	JX442434	GQ505099	GQ505208	
Platycercus venustus	Australia	JX442413	GQ505098	GQ505207	GQ505153
Poicephalus gulielmi	Africa	AY283498	JF807961	JF807988	JF807974
Poicephalus meyeri	Africa	GQ996511		JF807989	JF807975
Poicephalus rufiventris	Africa	MG736916	GQ505116	GQ505226	GQ505169
Poicephalus senegalus	Africa	AF346359		GQ505227	GQ505170
Polytelis alexandrae	Australia	JQ066206	GQ505093	GQ505201	GQ505147
Polytelis anthopeplus	Australia	AF346386	GQ505094	GQ505202	GQ505148
Prioniturus discurus	IAA	JQ066197	GQ505133		GQ505186
Prioniturus luconensis	IAA	NC_027846	GQ505134		
Prioniturus montanus	IAA	JQ066185	JF807962	JF807990	JF807976
Prosopeia tabuensis	Pacific	EU627173	GQ505105	GQ505214	
Psephotus chrysopterygius	Australia	JX442436	JF807963	JF807991	JF807977
Psephotus dissimilis	Australia	JX442415	GQ505101	GQ505210	GQ505155

Psephotus varius	Australia	JX442414	GQ505100	GQ505209	GQ505154
Psittacella brehmii	Australia IAA	JQ066220	JF807964	JF807992	JF807978
Psittacula alexandri alexandri	IAA	AB177970			
Psittacula alexandri fasciata	Asia	GQ996507			
Psittacula calthrapae	India	GQ996512			
Psittacula columboides	India	AY220108			
Psittacula cyanocephala	India	GQ996508		KJ456123	
Psittacula derbiana	Asia	AF346388	U88424		
Psittacula echo	Mauritius	AY220113			
Psittacula eques	Reunion	LN614517			
Psittacula eupatria	Asia / India / IAA	AY220115	GQ505137	GQ505246	GQ505189
Psittacula exsul	Rodrigues	LN614516			
Psittacula finschii	Asia	GQ996510			
Psittacula himalayana	Asia	AY220102		KJ456124	
Psittacula krameri borealis	Asia	AY220116			
Psittacula krameri krameri	Africa	AY220117			
Psittacula krameri manillensis	India	GQ996517			
Psittacula krameri parvirostris	Africa	GQ996497			
Psittacula longicauda	IAA	GQ996509			
Psittacula roseata	India	AY220107		KJ456126	
Psittacula wardi	Seychelles	GQ996500			
Psittaculirostris desmarestii	IAA	AB177960	GQ505131	GQ505242	GQ505184
Psittaculirostris edwardsii	Australia IAA	AB177971	GQ505132	GQ505243	GQ505185
Psittacus erithacus erithacus	Africa	AY082082	GQ505115	GQ505225	GQ505168
Psitteuteles goldiei	Australia IAA	KM372512	GQ505124	GQ505235	GQ505177
Psittinus cyanurus	IAA	JQ066222		GQ505247	GQ505190
Psittrichas fulgidus	IAA	AF346391	GQ505127	GQ505239	GQ505181
Purpureicephalus spurius	Australia	JX442410	GQ505096	GQ505204	GQ505150
Tanygnathus megalorhynchus	IAA	GQ996516	GQ505136	GQ505245	GQ505188
Trichoglossus johnstoniae	IAA		GQ505123	GQ505234	GQ505176
Triclaria malachitacea	America	AY669442	GQ505117	GQ505228	GQ505171
Anas		KX534428	AF478185	AF143729	EU738887
Anser		EU585613	AY994065	DQ137227	EU738899
Larus		AB208758	U88419	AY228799	EU738965
Coracias caudata		U89184	AY056916	AF143737	EU738931
Todus angustirostris		AF441626	AF441636	DQ111790	EU738886
Gavia		HQ864537	U88423	DQ137228	EU738953
Gallus		EU839454	AY056925	AF143730	EU738891
Falco		KP863030	AY447974	AY461399	AF490155
Acanthisitta chloris		AY325307	AY056903	AY056975	EU738893
Corvus corone		JQ864491	AY056918	AY056989	EF568306
Picathartes gymnocephalus		KJ909200	AY056950	AY057019	EF568314
Pipra coronata		FJ899382	AY056951	AY057020	AF492518

Pitta	KJ456408	AY056952	AY057021	EF568299
Scopus umbretta	KX534433	AF339323	DQ881830	EU739024
Pelecanus	MH041272	AF339325	DQ881819	EU738992
Phalacrocorax carbo	NC_027267	AF339332	DQ881821	EU738997
Phoenicopterus	KJ400319	AF339336	DQ881823	EU739001
Podiceps	NC_008140	AF339334	DQ881825	EU739009
Puffinus	AJ004215	U88421	DQ881827	AF490146
Eudyptes pachyrhynchus (Spheniscidiae)	DQ137210	U88420	DQ137231	
Eudyptula minor (Spheniscidiae)	MF370525			EU738944
Struthio camelus	NC_002785	U88429	AF143727	EU738886

In this analysis, we added some terminal taxa and sequences. By checking multiple runs, we found out that the *Coracopsis* species were acting as wildcards, changing their position across the tree. Most of the time, this genus was a sister-clade of *Mascarinus*. We checked the literature and concluded that it was a good approximation to enforce the monophyly of *Coracopsis*-*Mascarinus*-*Psittrichas* clade.

5. Pycnonotidae

Table S5: Genbank numbers for the sequences used in the Pycnonotidae analyses with the distribution attributed to each terminal.

Species	Location	12S	16S	ATP6	COI	CYTB	ND2	ND3	FIB-I5	FIB-I7	GAPDH-I11	MB-I2	ODC-I6/I7	RAG1	TGFB2-I5
Alophoixus bres	IAA	AF386490	AF391227	KT312509		JN827010	DQ402228	DQ402289		DQ402319	KT311996		KT312713		GU112563
Alophoixus finschii	IAA					KY404180	KY454707	KY454726				KY454741			KY454738
Alophoixus flaveolus	Asia	AF386478	AF391215	KT312498	JQ173973	KJ456190	KJ455318	KT312363			KT311974	KJ454749	KT312691	KJ455972	
Alophoixus frater	IAA						GU112679	GU112725		GU112633					GU112587
Alophoixus ochraceus	IAA	AF386482	AF391219	KT312419		KY404181	DQ402229	DQ402290		DQ402322	KT311798		KT312551		GU112564
Alophoixus pallidus	Asia	AF386471	AF391209	KT312428		DQ008507	GQ242078	GQ242112	EF626743	GU112600	KT311828	EF625281	KT312565		GU112531
Alophoixus phaeocephalus	IAA	AF386491	AF391228	KT312520		KY404182	DQ402230	DQ402291		DQ402323	KT312018		KT312735		GU112565
Andropadus curvirostris	Africa				JQ174018										
Andropadus gracilis	Africa				JQ174019										
Andropadus importunus	Africa				FJ473230	FJ487856	AF003469	GQ242115	EF626713	GQ242059		EF625252	EF625302		
Andropadus latirostris	Africa				JQ174020										
Arizelocichla chlorigula	Africa					AH005509	AF003457		EF626703			EF625243	EF625292		
Arizelocichla fusciceps	Africa					AH005510	AF003458		EF626700			EF625240	EF625289		
Arizelocichla kakamegae	Africa					AH005504	KY454708	KY454727				KY454742			
Arizelocichla kikuyensis	Africa					AH005516	AF003463		EF626701			EF625241	EF625290		
Arizelocichla masukensis	Africa						GQ242085	GQ242120	EF626698	GQ242064		JX236344	EF625287	JX236416	
Arizelocichla milanjensis	Africa						KY454709					KY454743			
Arizelocichla montana	Africa					AH005507	AF003455								
Arizelocichla neumanni	Africa						AF003456		EF626702			EF625242	EF625291		
Arizelocichla nigriceps	Africa					AH005511	AF003459								
Arizelocichla striifacies	Africa					AH005513	KY454710	KY454728	EF626704			KY454744	EF625293		
Arizelocichla tephrolaema	Africa					AF282785	GQ242086	GQ242121	GQ242044	GQ242065		GQ242109	GQ242147		
Atimastillas flavicollis	Africa					JX236375	DQ402205	DQ402266	EF626721	DQ402318	JX236287	JX236346	EF625310		
Baeopogon clamans	Africa						DQ402203	DQ402264	EF626716			EF625256	EF625305		
Baeopogon indicator	Africa	AF386465	AF391205				DQ402204	DQ402265	EF626717	DQ402317		EF625255	EF625306		

Bernieria madagascariensis	Madagascar					AF199387	DQ402194	DQ402255		DQ402338	HQ333100	HQ333071	HQ333086	JX236419	
Bleda canicapillus	Africa						DQ402201	DQ402262	GQ242043	DQ402315		GQ242108	GQ242146		GU112528
Bleda eximius	Africa				JQ174174		DQ402202	DQ402263		DQ402316					
Bleda notatus	Africa	AF386474	AF391203				KY454711	KY454729	EF626739			KY454745	EF625328		
Bleda syndactylus	Africa	AF386466	AF391204		JQ174177	KC355099	AY136592	AY590757	EF626738	GQ242063		EF625276	EF625327	AY319976	
Calyptocichla serinus	Africa						DQ402199	DQ402260	EF626715	DQ402314		EF625254	EF625304		GU112566
Cerasophila thompsoni	Asia						KY454712	KY454730				KY454746			
Chlorocichla falkensteini	Africa						KY454713	KY454731				KY454747			
Chlorocichla flaviventris	Africa					AY228053	GQ242087	AY590758	EF626720	GQ242066		AY228290	EF625309	AY228009	
Chlorocichla laetissima	Africa				HQ998101										
Chlorocichla prigoginei	Africa				HQ998159										
Chlorocichla simplex	Africa						KY454714	KY454732				KY454748			
Criniger barbatus	Africa	AF386487	AF391224		JQ174571		DQ402208	DQ402269	EF626740			EF625278	EF625329		
Criniger calurus	Africa	AF386483	AF391220		JQ174574		DQ402206	DQ402267				DQ125947			GU112529
Criniger chloronotus	Africa			JX259157			DQ402207	DQ402268		DQ402321		JX259184			
Criniger ndussumensis	Africa	AF386472	AF391210				DQ402209	DQ402270	EF626741	DQ402324		EF625279	EF625330		
Criniger olivaceus	Africa	AF386488	AF391225												
Eurillas ansorgei	Africa						DQ402195	DQ402256	EF626708	DQ402313		EF625246	EF625297		
Eurillas curvirostris	Africa						AH005522	DQ402259	EF626706	DQ402359		EF625247	EF625295		
Eurillas gracilis	Africa						DQ402196	DQ402257	EF626709	DQ402357		EF625245	EF625298		
Eurillas latirostris	Africa	AF386467	AF096477			DQ008508	GQ242089	GQ242124	EF626710	GQ242068			EF625299		
Eurillas virens	Africa	AF386477	AF391214		HQ998164	AH005519	DQ402197	DQ402258	EF626711	DQ402358		EF625250	EF625300		
Hemixos castanonotus	Asia						GU112647	GU112693		GU112601					GU112532
Hemixos cinereus	IAA						DQ402224	DQ402285	GQ242038			GQ242104	GQ242141		GU112567
Hemixos flavala	Asia					KJ456298	GU112648	GU112694		GU112602					GU112533
Hirundo rustica	Cosmopolite	AB042349	AB042382	GU460221	FJ027653	DQ119526	HF548562	DQ402247	EF626748	DQ402309	HQ333105	AY064258	EF441240	AY064271	
Hypsipetes amaurotis	Japan	EU167068			AB765881	AB159161	DQ402222	DQ402283	GQ242037	DQ402325		GQ242103	GQ242140		GU112570
Hypsipetes borbonicus	Reunion			AY590702				AY590733							
Hypsipetes crassirostris	Seychelles			AY590703				AY590741							

Hypsipetes everetti	IAA						GU112651	GU112697		GU112605					GU112536
Hypsipetes guimarasensis	IAA						GU112658	GU112704		GU112612					GU112543
Hypsipetes leucocephalus	Asia	AF386481				KJ456308	GU112649	GU112695	GQ242040	GU112603		GQ242105	GQ242143		GU112534
Hypsipetes madagascariensis	Madagascar Comoros	AB042346	AB042379	AY590704	JQ175126		DQ402225	DQ402286		DQ402328					GU112568
Hypsipetes mindorensis	IAA						GU112655	GU112701		GU112609					GU112540
Hypsipetes moheliensis	Comoros			AY590710				AY590737							
Hypsipetes olivaceus	Mauritius			AY590709				AY590735							
Hypsipetes parvirostris	Comoros			AY590711				AY590739							
Hypsipetes philippinus	IAA	AF386486	AF391223	AY590713			GU112654	GU112700	EF626742	GU112608		EF625280	EF625331		GU112539
Hypsipetes rufigularis	IAA						GU112660	GU112706		GU112614					GU112545
Hypsipetes siquijorensis	IAA						GU112661	GU112707		GU112615					GU112546
Iole charlottae	IAA						GU112690	GU112736	GQ242036	DQ402354		GQ242102	GQ242139		GU112598
Iole palawanensis	IAA						GU112688	GU112734		GU112642					GU112596
Iole propinqua	Asia IAA	AF386475	AF391212		JQ175164		GQ369691					GQ369646			
Iole viridescens	Asia						KU601856	KU601803		KU601909					
Ixonotus guttatus	Africa	AF386470	AF391208				GQ242088	GQ242123	EF626718	GQ242067		EF625257	EF625307		
Ixos malaccensis	IAA					KY404183	DQ402227	DQ402288		DQ402329					GU112569
Ixos mcclellandii	Asia	AF386468	AF391206			JX398905	GQ242079	GQ242113	GQ242039	GU112607	KJ455060	DQ008558	GQ242142	KJ456057	GU112538
Ixos philippinus	IAA				EU541456									EF568257	
Ixos virescens	IAA						KY454715	KY454733				KY454749			
Malia grata	IAA					JX398905	JX398921	JN826851							
Neolestes torquatus	Africa				HQ998140		GQ242083	GQ242117	GQ242041	GQ242061		GQ242106	GQ242144		
Nicator chloris	Africa				JQ175554	JX236396	DQ402188	DQ402249	EU680666			EU680603	EU680745	AY319991	
Phyllastrephus albigula	Africa						HQ716722		HQ716832						
Phyllastrephus albigularis	Africa	AF386476	AF391213				DQ402210	DQ402271	EF626734	DQ402330		EF625272	EF625323		
Phyllastrephus baumanni	Africa						KY454716								
Phyllastrephus cerviniventris	Africa					JX236400			EF626726			JX236363	EF625315	JX236447	
Phyllastrephus debilis	Africa					AF199383	DQ402213	DQ402274	EF626737	DQ402335	HQ716929	EF625275	EF625326		
Phyllastrephus fischeri	Africa						DQ402216	DQ402277	EF626730	DQ402339		EF625266	EF625319		

Phyllastrephus flavostriatus	Africa					AH005523	DQ402214	DQ402275	EF626735	DQ402336		EF625273	EF625324		
Phyllastrephus fulviventris	Africa						KY454717								
Phyllastrephus hypochloris	Africa						DQ402215	DQ402276	EF626727	DQ402337		EF625265	EF625316		
Phyllastrephus icterinus	Africa	AF386469	AF391207		JQ175790	AF199382	DQ402211	DQ402272	EF626731	DQ402331		EF625269	EF625320		GU112530
Phyllastrephus lorenzi	Africa				HQ998127				EF626732			EF625270	EF625321		
Phyllastrephus placidus	Africa						DQ402212	DQ402273	EF626729	DQ402332		EF625268	EF625318		
Phyllastrephus poensis	Africa						GQ242084	GQ242118	GQ242042	GQ242062		GQ242107	GQ242145		
Phyllastrephus scandens	Africa					AF199381	DQ402218	DQ402279	EF626723			EF625261	EF625312		
Phyllastrephus strepitans	Africa								EF626725			EF625263	EF625314		
Phyllastrephus terrestris	Africa						DQ402217	DQ402278	EF626724			EF625262	EF625313		
Phyllastrephus xavieri	Africa						DQ402219	AY590761	EF626733	DQ402340		EF625271	EF625322		
Pycnonotus atriceps	IAA	AF386484	AF391221			KY404184	DQ402231	DQ402292	GQ242029	DQ402341		GQ242096	GQ242132		GU112571
Pycnonotus aurigaster	Asia IAA						KY454718	KY454734				KY454750			
Pycnonotus barbatus	Africa	AF386479	AF391216		FJ473231	FJ487857	DQ402232	DQ402293	EF626746	DQ402342	FJ357922	EF625284	EF625335	AY057027	GU112572
Pycnonotus bimaculatus	IAA						KP943466								
Pycnonotus blanfordi	Asia IAA				JQ176058		KY454719	KY454735		KP943348		KY454751			KP943473
Pycnonotus brunneus	IAA		AY574891		FJ473091	FJ487694	DQ402233	DQ402294		DQ402343					GU112573
Pycnonotus cafer	India	KF289822	KF289828		JF498896	KJ456440	KJ455616				KJ455154	KJ454895	KJ455904	KJ456130	
Pycnonotus capensis	Africa								EF626745			EF625283	EF625334		
Pycnonotus cinereifrons	IAA						GU112684	GU112730		GU112638					GU112592
Pycnonotus cyaniventris	IAA					KY404185	DQ402234	DQ402295		DQ402344					GU112574
Pycnonotus dispar	IAA						KP943427								
Pycnonotus erythropthalmos	IAA					FJ487794	DQ402235	DQ402296		DQ402345					GU112575
Pycnonotus eutilotus	IAA		AY574900			KY404186	DQ402236	DQ402297	GQ242030	DQ402346		GQ242097	GQ242133		GU112576
Pycnonotus finlaysoni	IAA	AF386473	AF391211		FJ473154	FJ487764	GQ242076	GQ242110	GQ242033	GQ242053		GQ242100	GQ242136		
Pycnonotus flavescens	Asia					KY404187	GU112669	GU112715		GU112623					GU112554
Pycnonotus flaviventris	Asia India	KJ186975	KJ186975	KJ186975	KJ186975	KJ186975	KJ186975	KJ186975	GQ242032			GQ242099	GQ242135		
Pycnonotus goiavier	IAA				FJ473057	FJ487665	DQ402237	DQ402298		DQ402347					KY454739
Pycnonotus gularis	India						KP943372								

Pycnonotus jocosus	Asia IAA India	AF386480	AF391217		GU170351	KJ456441	GQ242077	GQ242111	GQ242034	GU112624	KJ455155	DQ008557	GQ242137	KJ456131	GU112555
Pycnonotus leucogenys	Asia India				HQ168045		DQ402241	DQ402302		DQ402351	KJ455156	KJ454896	KJ455905	EF568258	GU112578
Pycnonotus leucogrammicus	IAA						KP943465								
Pycnonotus leucotis	Arabic Peninsula India						KP943428								
Pycnonotus luteolus	India						KP943367								
Pycnonotus melanicterus	India						KY454720							KJ456132	
Pycnonotus melanoleucos	IAA						DQ402242	DQ402303		DQ402352					GU112579
Pycnonotus montis	IAA					KY404188	DQ402243	DQ402304		DQ402353	KJ455157				
Pycnonotus nigricans	Africa						DQ402238	DQ402299		DQ402348					GU112580
Pycnonotus plumosus	IAA		AY574884		FJ473241	FJ487748	DQ402239	DQ402300	JN826138	DQ402349					JN826387
Pycnonotus priocephalus	India						KY454721								
Pycnonotus simplex	IAA				FJ473143	FJ487754	GU112671	GU112717		GU112625					GU112556
Pycnonotus sinensis	Asia	GU475148	GU475148	GU475148	GU475148	GU475148	GU475148	GU475148		GU112626					GU112557
Pycnonotus squamatus	IAA					KY404189	KY454722	GU112737		GU112645					KY454740
Pycnonotus striatus	Asia					KJ456444	KJ455620				KJ455158	KJ454898	KJ455907	KJ456133	
Pycnonotus taivanus	Taiwan	FJ378536	FJ378536	FJ378536	FJ378536	FJ378536	FJ378536	FJ378536							
Pycnonotus tricolor	Africa						KY454723	KY454736				KY454752			
Pycnonotus urostictus	IAA				EU541463		KJ931003	AY590762	EF626744	GU112629		EF625282	EF625333		GU112560
Pycnonotus xanthopygos	Arabic Peninsula						KP943432								
Pycnonotus xanthorrhous	Asia	AF386485	AF391222				GU112677	GU112723		GU112631					GU112562
Pycnonotus zeylanicus	IAA						DQ402240	DQ402301		DQ402350					GU112582
Setornis criniger	IAA					KY404190	DQ402221	DQ402282		DQ402355					GU112583
Spizixos canifrons	Asia						KY454724	KY454737							
Spizixos semitorques	Asia				FJ661100	JF509584	DQ402244	DQ402305	GQ242031	DQ402356		GQ242098	GQ242134		GU112584
Stelgidillas gracilirostris	Africa						GQ242082	GQ242116	EF626705	GQ242060		EF625249	EF625294		
Thapsinillas affinis	IAA						KY454725								
Thescelocichla leucopleura	Africa						DQ402200	DQ402261	EF626722	DQ402312		EF625260	EF625311		
Tricholestes criniger	IAA	AF386489	AF391226			KY404191	DQ402223	DQ402284	GQ242035	DQ402326		GQ242101	GQ242138		GU112585

The calibration for this analysis was based on a Passeriformes dating analysis (Ericson, Klopfstein, Irestedt, Nguyen, & Nylander, 2014). Thus, a prior was set on the root with a lognormal distribution (M = 3.631 and S = 0.13), so that the median would be equal to 37.8 m.y.a. and the 95% confidence interval would be between 29 and 48 m.y.a. These dates represent the estimated origin date of the sylvioidea, the less inclusive group that includes the Pycnonotidae and the outgroup (Alström, Ericson, Olsson, & Sundberg, 2006).

6. Atyidae

Table S6: Genbank numbers for the sequences used in the Atyidae analyses with the distribution attributed to each terminal.

Species	Locality	16S	28S	H3
Antecaridina lauensis	Australia IAA Pacific	EU123851		FN995457
Antecaridina sp	Australia IAA Pacific	FN995353		FN995458
Antecaridina sp	Australia IAA Pacific	EU123853		FN995459
Atya gabonensis	Africa America	EF489961	FN995546	FN995460
Atya gabonensis	Africa America	EF489989	FN995547	FN995461
Atya margaritacea	America	EF489983	FN995548	FN995462
Atya ortmannioides	America	EF489993	FN995549	FN995463
Atya scabra	America	EF489985	FN995550	FN995465
Atyaephyra desmarestii	Europe	FN995354	FN995551	FN995466
Atyella brevirostris	Africa		FN995552	FN995468
Atyoida bisulcata	Pacific	EF489995	FN995553	FN995469
Atyoida pilipes	Pacific	DQ681277	FN995554	FN995470
Atyoida pilipes	Pacific	DQ681279	FN995555	FN995471
Atyopsis moluccensis	IAA	DQ681280	FN995557	FN995473
Atyopsis spinipes	IAA Pacific	DQ681282		
Australatya striolata	Australia	AY795035	FN995558	FN995474
Caridella paski	Africa	FN995355	FN995559	FN995475
Caridina cantonensis	Asia	DQ478487	FN995560	FN995476
Caridina celebensis	IAA Pacific	FN995356	FN995561	FN995477
Caridina cf africana	Africa	DQ478483	FN995562	FN995478
Caridina endehensis	IAA	FN995357	FN995563	FN995479
Caridina ensifera	IAA	AM747734	FN995564	FN995480
Caridina gracilipes	Asia IAA	FN995358	FN995565	FN995481
Caridina gracilirostris	IAA India Madagascar	EU873516	FN995566	FN995482
Caridina serrata	Asia	DQ478512		FN995483
Caridina serratirostris	Comoros IAA Madagascar Pacific	DQ478513	FN995567	FN995484
Caridina serratirostris	Comoros IAA Madagascar Pacific	DQ478514	FN995568	FN995485
Caridina serratirostris	Comoros IAA Madagascar Pacific	DQ478516	FN995569	FN995486
Caridina sp	Australia	DQ478534	FN995570	EU123809
Caridina sp2	Asia	FN995359	FN995571	
Caridina steineri	Madagascar	DQ681274		

Caridina sumatrensis	IAA	FN995360	FN995572	FN995487
Caridina thomasi	IAA	EU873523	FN995573	FN995488
Caridina togoensis	Africa	FN995361	FN995574	FN995489
Caridina trifasciata	Asia	FN995362	FN995575	FN995490
Caridina typus ARC	Australia IAA India Mascarene Pacific Seychelles	KY069381	KY069755	
Caridina typus SUL	IAA	KY069384	KY069757	
Caridina typus TAL	IAA	KY069360	KY069741	
Caridina villadolidi	IAA	KY436221		
Caridinides wilkinsi	Australia	DQ681273	FN995577	FN995492
Caridinopsis chevalieri	Africa		FN995578	
Dugastella marocana	Africa	FJ594357	FN995579	FN995494
Dugastella marocana	Africa	FN995363	FN995580	FN995495
Dugastella valentina	Europe	FN995364	FN995581	FN995496
Dugastella valentina	Europe	FN995365	FN995582	FN995497
Edoneus marulas	IAA	FN995366	FN995583	FN995498
Edoneus marulas	IAA	FN995367	FN995584	FN995499
Gallocaris inermis	Europe	DQ641599		
Halocaridina rubra	Pacific	FN995368	FN995585	FN995500
Halocaridinides trigonophthalma	Pacific	FN995369	FN995586	FN995501
Jonga serrei	America	EF490003	FN995587	FN995502
Lancaris singhalensis	India	FN995370	FN995588	FN995503
Limnocaridina parvula	Africa	FN995371	FN995589	FN995504
Limnocaridina similis	Africa	FN995372	FN995590	FN995505
Limnocaridina tanganyikae	Africa	FN995373	FN995591	FN995506
Marosina longirostris	IAA	FN995374	FN995592	FN995507
Marosina longirostris	IAA	FN995375	FN995593	FN995508
Micratya poeyi	America	EF489991	FN995595	FN995510
Micratya sp	America	EF489994	FN995596	FN995511
Neocaridina cf zhangjiajiensis	Asia		FN995597	FN995512
Neocaridina denticulata	Asia	DQ681268	FN995598	FN995513
Neocaridina denticulata	Asia	FN995377	FN995599	FN995514
Neocaridina palmata	Asia		FN995601	FN995516
Neocaridina saccam	Asia	DQ681270		
Palaemonias alabamae	America	FN995378	FN995602	FN995517
Palaemonias alabamae	America	FN995379	FN995603	FN995518
Palaemonias alabamae	America	FN995380	FN995604	FN995519
Palaemonias alabamae	America	FN995381	FN995605	FN995520
Palaemonias sp	America	FN995382	FN995606	FN995521
Palaemonias sp2	America	FN995383	FN995607	FN995522
Paracaridina sp	Asia	FN995384	FN995608	FN995523
Paratya australiensis	Australia	EF490007	FN995610	FN995525
Paratya curvirostris	New Zealand	AY661475	FN995611	FN995526
Parisia gracilis	Australia	EU123843	FN995612	EU123810

Parisia unguis	Australia	DQ681288		FN995527
Potimirim glabra	America	EF489999	FN995613	FN995528
Potimirim potimirim	America	FN995386	FN995614	FN995529
Potimirim sp	America	FN995387	FN995615	FN995530
Potimirim sp2	America	EF490000	FN995616	FN995531
Pycneus morsitans	Australia	EU123849	FN995617	EU123849
Pycnisia raptor	Australia	DQ681271	FN995618	FN995532
Sinodina sp	Asia	FN995388	FN995619	FN995533
Stygiocaris lancifera	Australia	EU123826	FN995620	EU123804
Stygiocaris sp	Australia	EU123840	FN995621	EU123807
Stygiocaris stylifera	Australia	EU123836	FN995622	EU123806
Syncaris pacifica	America	FN995389	FN995623	FN995534
Syncaris pacifica	America	FN995390	FN995624	FN995535
Troglocaris anophtalmus	Europe	DQ641587	DQ641614	
Troglocaris bosnica	Europe	DQ641588	DQ641615	
Troglocaris kutaissiana	Europe	DQ320026		
Troglocaris planinensis	Europe	DQ320024		
Troglocaris prasence	Europe	DQ641592	DQ641618	
Troglocaris pretneri	Europe	DQ641590	DQ641616	
Troglocaris sp	Europe	DQ641593	DQ641619	
Typhlatya miravetensis	Europe	DQ641602	DQ641626	
Typhlatya mitchelli	America	FN995393	FN995627	FN995538
Typhlatya rogersi	America	FN995394	FN995628	FN995539
Typhlatya sp	America	FN995395	FN995629	FN995540
Typhlatya sp2	America	FN995396	FN995630	FN995541
Xiphocaris elongata	America	EF490010	FN995631	FN995542
Xiphocaris elongata	America	EF490011	FN995632	FN995543

In the alignments, the three clades from Bernardes et al. (2017) were treated as separated terminals. Two calibration points were used from the three alternatives in the original article (von Rintelen et al., 2012): *Atyoida roxoi* was assigned to stem of Atyidae with a lognormal distribution (M = 1.25 and S = 0.68) set to encompass the Aptian age (offset = 112 m.y.a.); the secondary calibration from Porter, Pérez-Losada, & Crandall (2005) was assigned to the root with a lognormal distribution (M=3.95 and S = 0.30; offset = 217 m.y.a.). The calibration based in the Palaemonidae fossils was not used because we decided to reduce the dataset to simplify the analyses.

7. Caecilia

The sequences used in the Caecilia analyses were downloaded directly from the original article's dryad repository (https://datadryad.org/resource/doi:10.5061/dryad.jm453), separated from other amphibian groups, and re-aligned. Proopiomelanocortin and Rhodopsin genes were removed from the analyses because they represented no/little data for the caecilians specifically. We used several points of calibration. First, we used calibration points for Tetrapoda (lognormal distribution; M = 1.8 and S = 0.44; offset = 337 m.y.a.) and for Batrachia (exponential distribution; mean = 8.0 and

offset = 260 m.y.a.) based on Benton and Donoghue (2007), Pyron (2014), Benton et al. (2015), and Feng et al. (2017). We placed a calibration in the Caecilia most recent ancestor node (lognormal distribution; M = 1.1 and S = 1.63; offset = 100 m.y.a.) based on Wiens (2011). In the original article, these dates were obtained with a dated amphibian tree and not only were these analyses robust but they were also in agreement with several other studies we analysed (e.g. (Feng et al., 2017). Calibration using the fossil from Early Cretaceous *Eocaecilia micropodia* (Jenkins & Walsh, 1993) was tested by assigning it either to the node of the Lissamphibia ancestor with a lognormal distribution or to the node of the Caecilia ancestor (M = 1.6 and S = 0.87; offset = 174 m.y.a.). *Eocaecilia* is stem-group caecilian (Maddin, Jenkins Jr, & Anderson, 2012) and should not be used to date the crown-group. Our tests showed that adding *Eocaecilia* in the Lissamphibia node made no difference to the final result. However, to treat *Eocaecilia* as a crown-group caecilian places the divergence between Indian and Seychellois taxa in a period that is congruent with the geological dates of the India-Seychelles separation.

8. Neobatrachia

Table S7: Genbank numbers for the sequences used in the Neobatrachia analyses with the distribution attributed to each terminal.

Species	Locality	CILP	DISP2	DMXL1	DSEL	EVPL	EXOC8	HYP	MSH6	PPL	RAG1	RAG2	SALL1	WFIKKN2	ZFPM2	CXCR4
Acris crepitans	Seychelles	KX200136	KX201145	KX201295	KX201817	KX202098	KX202244	KX204391	KX207070	KX208566	KX208710	KX208856	KX209700	KX211266	KX211700	KX200424
Adenomus kelaartii	India											EF107284				EF107447
Afrixalus dorsalis	Africa											DQ347236				EF107456
Agalychnis callidryas	Seychelles	KX200097	KX201101	KX201246	KX201770	KX202055	KX202199	KX204345	KX207026	KX208518	KX208666	KX208812	KX209657	KX211218	KX211654	KX200375
Agalychnis lemur	Seychelles	KX200085	KX201089	KX201232	KX201756	KX202041	KX202184	KX204333	KX207015	KX208503	KX208652	KX208799	KX209643	KX211203	KX211638	KX200360
Agalychnis saltator	Seychelles											EF174315				GQ365983
Aglyptodactylus madagascariensis	Madagascar	KX200146	KX201156	KX201306	KX201828	KX202109	KX202255	KX204402	KX207080	KX208576	KX208721	KX208867	KX209711	KX211277	KX211711	KX200434
Allobates femoralis	Seychelles		KX201120	KX201270	KX201792	KX202073	KX202221	KX204367	KX207047	KX208541	KX208686	KX208833	KX209675	KX211240	KX211675	KX200398
Allophryne ruthveni	Seychelles											EU663432				KF534373
Alsodes gargola	Seychelles	KX200128	KX201134	KX201284	KX201806	KX202086	KX202235	KX204381	KX207061	KX208555	KX208700	KX208845	KX209689	KX211254	KX211688	KX200412
Alytes obstetricans	America	KX200147	KX201157	KX201307	KX201829	KX202110	KX202256	KX204403	KX207081	KX208577	KX208722		KX209712	KX211278	KX211712	KX200435
Amazophrynella minuta	Seychelles										DQ158346				KX211639	DQ306496
Amietia lubrica	Africa	KX200201	KX201212	KX201362	KX201886	KX202164	KX202312	KX204460	KX207133	KX208633	KX208779	KX208918	KX209770	KX211334	KX211769	KX200492
Amolops loloensis	Asia	KX200118	KX201124	KX201274	KX201796	KX202077	KX202225	KX204371	KX207051	KX208545	KX208690	KX208836	KX209679	KX211244		KX200402
Amolops ricketti	Asia		KX201159	KX201309	KX201831		KX202258	KX204405	KX207083	KX208579	KX208724	KX208869	KX209714	KX211280	KX211714	KX200437
Anaxyrus canorus	Seychelles		KX201093		KX201760	KX202045	KX202188	KX204337	KX207018	KX208507	KX208656	KX208803	KX209647	KX211207	KX211643	KX200364
Anaxyrus punctatus	Seychelles	KX200104	KX201109	KX201258	KX201781	KX202061	KX202210	KX204355	KX207035	KX208529	KX208674		KX209664	KX211229	KX211663	KX200386
Andrias davidianus		KX200159	KX201170	KX201319	KX201842	KX202121		KX204416	KX207093	KX208590	KX208734	KX208880	KX209725	KX211291	KX211725	KX200447
Anodonthyla boulengerii	Madagascar			KX201252	KX201776		KX202205		KX207031	KX208524						
Aplastodiscus perviridis	Seychelles	KX200126	KX201132	KX201282	KX201804	KX202084	KX202233	KX204379	KX207059	KX208553	KX208698	KX208843	KX209687	KX211252	KX211686	KX200410
Arthroleptis poecilonotus	Africa	KX200148	KX201158	KX201308	KX201830	KX202111	KX202257	KX204404	KX207082	KX208578	KX208723	KX208868	KX209713	KX211279	KX211713	KX200436
Arthroleptis variabilis	Africa											EF396073				AY364180
Ascaphus truei	Seychelles	KX200149	KX201160	KX201310	KX201832	KX202112		KX204406	KX207084	KX208580	KX208725	KX208870	KX209715	KX211281	KX211715	KX200438
Astylosternus diadematus	Africa	KX200145	KX201155	KX201305	KX201827	KX202108	KX202254	KX204401	KX207079		KX208720	KX208866	KX209710	KX211276	KX211710	KX200433
Atelognathus reverberii	Seychelles	KX200139	KX201149	KX201299	KX201821	KX202102	KX202248	KX204395	KX207074	KX208570	KX208714	KX208860	KX209704	KX211270	KX211704	KX200428
Atelopus peruensis	Seychelles											DQ158345				DQ306495
Aubria subsigillata	Africa	KX200138	KX201147	KX201297	KX201819	KX202100	KX202246	KX204393	KX207072	KX208568	KX208712	KX208858	KX209702	KX211268	KX211702	KX200426
Babina chapaensis	Asia											EU076752				KR264317
Babina pleuraden	Asia											KR264384				KR264303
Barbourula busuangensis	IAA	KX200143	KX201153	KX201303	KX201825	KX202106	KX202252	KX204399	KX207077	KX208574	KX208718	KX208864	KX209708	KX211274	KX211708	KX200431
Barycholos pulcher	Seychelles	KX200094	KX201099	KX201241	KX201766	KX202051	KX202194	KX204343	KX207023	KX208513	KX208662	KX208808	KX209653	KX211213	KX211649	KX200370
Batrachophrynus macrostomus	Seychelles	KX200122	KX201128	KX201278	KX201800	KX202081	KX202229	KX204375	KX207055	KX208549	KX208694		KX209683	KX211248	KX211682	KX200406
Batrachyla leptopus	Seychelles	KX200134	KX201142	KX201292	KX201814	KX202095	KX202242	KX204389	KX207067	KX208563	KX208707		KX209697	KX211263	KX211697	KX200421
Batrachyla taeniata	Seychelles	KX200141	KX201151	KX201301	KX201823	KX202104	KX202250	KX204397		KX208572	KX208716	KX208862	KX209706	KX211272	KX211706	KX200429
Blommersia wittei	Madagascar											AY323774				AB612028
Bombina fortinuptialis	Asia America	KX200116	KX201122	KX201272	KX201794	KX202075	KX202223	KX204369	KX207049	KX208543	KX208688	KX208835	KX209677	KX211242	KX211677	KX200400

Bombina orientalis	Asia America	KX200154	KX201165	KX201315	KX201837	KX202117	KX202263	KX204411	KX207089	KX208585	KX208730	KX208875	KX209720	KX211286	KX211720	KX200442
Boophis madagascariensis	Comoros Madagascar	KX200095		KX201242	KX201767		KX202195			KX208514		KX208809		KX211214	KX211650	KX200371
Boophis xerophilus	Comoros Madagascar											AY364209				AY364179
Brachycephalus ephippium	Seychelles											GQ345275				GQ345180
Brachytarsophrys feae	Asia	KX200150	KX201161	KX201311	KX201833	KX202113	KX202259	KX204407	KX207085	KX208581	KX208726	KX208871	KX209716	KX211282	KX211716	
Breviceps macrops	Africa	KX200152	KX201163	KX201313	KX201835	KX202115	KX202261	KX204409	KX207087	KX208583	KX208728	KX208873	KX209718	KX211284	KX211718	KX200440
Breviceps mossambicus	Africa											EF396076				EF017965
Buergeria buergeri	Asia											AB612031				AB612035
Buergeria oxycephala	Asia	KX200090	KX201095	KX201237	KX201762	KX202047	KX202190	KX204339	KX207020	KX208509	KX208658	KX208805	KX209649	KX211209	KX211645	KX200366
Bufo bufo	America											AY583336				DQ306504
Bufo gargarizans	Asia	KX200151	KX201162	KX201312	KX201834	KX202114	KX202260	KX204408	KX207086	KX208582	KX208727	KX208872	KX209717	KX211283	KX211717	KX200439
Bufo japonicus	Asia											AB612057				AB612061
Bufotes viridis	Asia India America											KJ609682				FJ882714
Calluella guttulata	IAA											AB611861				EF017975
Callulina kreffti	Africa		KX201143	KX201293	KX201815	KX202096			KX207068	KX208564	KX208708	KX208854	KX209698	KX211264	KX211698	KX200422
Callulina laphami	Africa											KF990044				KF954751
Callulops wilhelmanus	IAA		KX201102	KX201248	KX201772		KX202201	KX204347	KX207027	KX208520	KX208667	KX208814		KX211220	KX211655	KX200377
Calyptocephalella gayi	Seychelles	KX200113	KX201118	KX201268	KX201790	KX202071	KX202219	KX204365	KX207045	KX208539	KX208684	KX208831	KX209673	KX211238	KX211673	KX200396
Centrolene bacatum	Seychelles											EU663437				KF534379
Centrolene daidaleum	Seychelles											EU663465				KF534381
Ceratophrys cornuta	Seychelles	KX200155	KX201166	KX201316	KX201838	KX202118	KX202264	KX204412		KX208586	KX208731	KX208876	KX209721	KX211287	KX211721	KX200443
Ceratophrys ornata	Seychelles											AY364218				AY364188
Ceuthomantis smaragdinus	Seychelles											GQ345287				GQ345192
Chalcorana macrops	IAA											KR264416				KR264339
Chaperina fusca	IAA											AB611865				AB611870
Chiasmocleis ventrimaculata	Seychelles	KX200089	KX201094	KX201236	KX201761	KX202046	KX202189	KX204338	KX207019	KX208508	KX208657	KX208804	KX209648	KX211208	KX211644	KX200365
Chimerella mariaelenae	Seychelles											EU663449				KF534391
Cochranella granulosa	Seychelles											EF107297				EF107460
Conraua crassipes	Africa	KX200156	KX201167	KX201317	KX201839	KX202119	KX202265	KX204413	KX207090	KX208587	KX208732	KX208877	KX209722	KX211288	KX211722	KX200444
Cophixalus cheesmanae	Australia IAA	KX200142	KX201152	KX201302	KX201824	KX202105	KX202251	KX204398	KX207076	KX208573	KX208717	KX208863	KX209707	KX211273	KX211707	KX200430
Cophixalus cryptotympanum	Australia IAA											AB611873				AB611878
Cophixalus sp.	Australia IAA	KX200099	KX201104	KX201250	KX201774	KX202057	KX202203	KX204349	KX207029	KX208522	KX208669	KX208816	KX209659	KX211222	KX211657	KX200379
Cophixalus sp. B	Australia IAA											DQ347276				EF017967
Cornufer pelewensis	Australia IAA	KX200197	KX201207	KX201357	KX201881	KX202160	KX202307	KX204455	KX207129	KX208628	KX208774	KX208915	KX209765	KX211329	KX211764	KX200487
Craugastor augusti	Seychelles		KX201177	KX201326	KX201849	KX202128	KX202274	KX204423	KX207099	KX208597	KX208741	KX208887	KX209732	KX211298	KX211732	KX200454
Craugastor fitzingeri	Seychelles	KX200120	KX201126	KX201276	KX201798	KX202079	KX202227	KX204373	KX207053	KX208547	KX208692	KX208838	KX209681	KX211246	KX211680	KX200404
Craugastor podiciferus	Seychelles											GQ345277				GQ345182
Crinia signifera	Australia	KX200158	KX201169		KX201841		KX202267	KX204415	KX207092	KX208589	KX208733	KX208879	KX209724	KX211290	KX211724	KX200446
Cruziohyla calcarifer	Seychelles											EF174317				GQ365984
Cryptobatrachus boulengeri	Seychelles	KX200157	KX201168	KX201318	KX201840	KX202120	KX202266	KX204414	KX207091	KX208588		KX208878	KX209723	KX211289	KX211723	KX200445
Ctenophryne geayi	Seychelles											AB611881				AB611886

Cyclorana maini	Australia											EF107311				EF107475
Dendrobates auratus	Seychelles											AY364214				AY364184
Dendropsophus parviceps	Seychelles	KX200110	KX201115	KX201265	KX201788	KX202068	KX202216	KX204362	KX207042	KX208536	KX208681	KX208828	KX209670	KX211235	KX211670	KX200393
Diasporus diastema	Seychelles											GQ345279				GQ345184
Discoglossus pictus	America	KX200093	KX201098	KX201240	KX201765	KX202050	KX202193	KX204342		KX208512	KX208661		KX209652	KX211212	KX211648	KX200369
Duttaphrynus melanostictus	Asia IAA India	KX200153	KX201164	KX201314	KX201836	KX202116	KX202262	KX204410	KX207088	KX208584	KX208729	KX208874	KX209719	KX211285	KX211719	KX200441
Duttaphrynus stomaticus	Asia IAA India											KJ609680				FJ882681
Dyscophus antongilii	America	KX200160	KX201171	KX201320	KX201843	KX202122	KX202268	KX204417		KX208591	KX208735	KX208881	KX209726	KX211292	KX211726	KX200448
Elachistocleis ovalis	Seychelles	KX200161	KX201172	KX201321	KX201844	KX202123	KX202269	KX204418	KX207094	KX208592	KX208736	KX208882	KX209727	KX211293	KX211727	KX200449
Eleutherodactylus coqui	Seychelles											EF107341				EF107500
Eleutherodactylus marnockii	Seychelles											EF107300				EF107463
Eleutherodactylus planirostris	Seychelles	KX200130	KX201137	KX201288	KX201809	KX202090	KX202237	KX204384	KX207063	KX208558		KX208849	KX209692	KX211258	KX211692	KX200416
Epidalea calamita	America											EU497610				FJ882709
Epipedobates tricolor	Seychelles											EF107295				EF107458
Espadarana prosoblepon	Seychelles											AY364223				AY364193
Euphlyctis cyanophlyctis	Asia India America											AB488957				AB488919
Eupsophus calcaratus	Seychelles	KX200105	KX201110	KX201259	KX201782	KX202062	KX202211	KX204356	KX207036	KX208530	KX208675	KX208822	KX209665	KX211230	KX211664	KX200387
Fejervarya granosa	Asia IAA India											AB488947				AB488913
Fejervarya limnocharis	Asia IAA India	KX200163	KX201174	KX201323	KX201846	KX202125	KX202271	KX204420	KX207096	KX208594	KX208738	KX208884	KX209729	KX211295	KX211729	KX200451
Fejervarya multistriata	Asia IAA India											AB500221				AB500243
Flectonotus fitzgeraldi	Seychelles											DQ679274				GQ345177
Gastrophryne olivacea	Seychelles	KX200164	KX201175	KX201324	KX201847	KX202126	KX202272	KX204421	KX207097	KX208595	KX208739	KX208885	KX209730	KX211296	KX211730	KX200452
Gastrophrynoides immaculatus	IAA											AB611901				AB611906
Gastrotheca pseustes	Seychelles	KX200165	KX201176	KX201325	KX201848	KX202127	KX202273	KX204422	KX207098	KX208596	KX208740	KX208886	KX209731	KX211297	KX211731	KX200453
Gastrotheca weinlandii	Seychelles			KX201244	KX201769	KX202053	KX202197			KX208516	KX208664		KX209655	KX211216	KX211652	KX200373
Heleophryne purcelli	Africa	KX200169	KX201181	KX201330	KX201853	KX202132	KX202278	KX204427	KX207103		KX208745	KX208891	KX209736	KX211302	KX211736	KX200458
Hemiphractus bubalus	Seychelles											DQ679303				GQ345179
Hemisus marmoratus	Africa	KX200168	KX201180	KX201329	KX201852	KX202131	KX202277	KX204426	KX207102	KX208600	KX208744	KX208890	KX209735	KX211301	KX211735	KX200457
Hoplobatrachus tigerinus	Africa Asia India	KX200207	KX201218	KX201366	KX201892	KX202170	KX202318	KX204465	KX207139	KX208639	KX208785	KX208924	KX209776	KX211340	KX211775	KX200498
Hoplophryne rogersi	Africa											EF396089				EF017980
Hyalinobatrachium aureoguttatum	Seychelles											EU663491				KF534403
Hyalinobatrachium colymbiphyllum	Seychelles											EU663498				KF534408
Hyalinobatrachium ibama	Seychelles											EU663507				KF534413
Hydrophylax leptoglossa	India											KR264388				KR264308
Hyla arenicolor	Asia America Seychelles											AY364220				AY364190
Hyla chinensis	Asia America Seychelles	KX200167	KX201179	KX201328	KX201851	KX202130	KX202276	KX204425	KX207101	KX208599	KX208743	KX208889	KX209734	KX211300	KX211734	KX200456
Hyla cinerea	Asia America Seychelles											AY323766				DQ306493
Hylarana sp.	India IAA	KX200119	KX201125	KX201275	KX201797	KX202078	KX202226	KX204372	KX207052	KX208546	KX208691	KX208837	KX209680	KX211245	KX211679	
Hylodes nasus	Seychelles											KJ961597				GQ345194
Hylophorbus rufescens	Australia											EF018047				EF017977
Hyloscirtus lindae	Seychelles	KX200109	KX201114	KX201264	KX201787	KX202067	KX202215	KX204361	KX207041	KX208535	KX208680	KX208827	KX209669	KX211234	KX211669	KX200392

Hyloxalus jacobuspetersi	Seychelles		KX201135	KX201285	KX201807	KX202087		KX204382		KX208556	KX208701	KX208846	KX209690	KX211255	KX211689	KX200413
Hymenochirus boettgeri	Africa	KX200170	KX201182	KX201331	KX201854	KX202133	KX202279	KX204428	KX207104	KX208601	KX208746	KX208892	KX209737	KX211303	KX211737	KX200459
Hyperolius bolifambae	Africa	KX200166	KX201178	KX201327	KX201850	KX202129	KX202275	KX204424	KX207100	KX208598	KX208742	KX208888	KX209733	KX211299	KX211733	KX200455
Hyperolius viridiflavus	Africa											AY323769				AB612019
Hypodactylus brunneus	Seychelles	KX200106	KX201111	KX201260	KX201783	KX202063	KX202212	KX204357	KX207037	KX208531	KX208676	KX208823	KX209666	KX211231	KX211665	KX200388
Hypsiboas fasciatus	Seychelles	KX200098	KX201103	KX201249	KX201773	KX202056	KX202202	KX204348	KX207028	KX208521	KX208668	KX208815	KX209658	KX211221	KX211656	KX200378
Ikakogi tayrona	Seychelles											EU663455				KF534427
Incilius luetkenii	Seychelles											KJ609668				DQ306565
Incilius nebulifer	Seychelles	KX200108	KX201113	KX201262	KX201785	KX202065	KX202214	KX204359	KX207039	KX208533	KX208678	KX208825	KX209668	KX211233	KX211667	KX200390
Indirana sp. A	India											DQ347204				EF017984
Indirana sp. B	India											DQ347215				EF017985
Ingerophrynus divergens	Asia IAA											EU712818				FJ882701
Ingerophrynus galeatus	Asia IAA											DQ158374				DQ306506
Insuetophrynus acarpicus	Seychelles	KX200103	KX201108	KX201256	KX201779	KX202060	KX202208	KX204354	KX207034	KX208527	KX208673	KX208820	KX209663	KX211227	KX211662	KX200384
Ischnocnema guentheri	Seychelles											KC468335				GQ345181
Kalophrynus interlineatus	Asia IAA											AB611909				AB611914
Kalophrynus pleurostigma	Asia IAA	KX200171	KX201183	KX201332	KX201855	KX202134	KX202280	KX204429	KX207105	KX208602	KX208747		KX209738	KX211304	KX211738	KX200460
Kaloula conjuncta	Asia IAA			KX201263	KX201786	KX202066		KX204360	KX207040	KX208534	KX208679	KX208826			KX211668	KX200391
Kaloula pulchra	Asia IAA	KX200172	KX201184	KX201333	KX201856	KX202135	KX202281	KX204430	KX207106	KX208603	KX208748	KX208893	KX209739	KX211305	KX211739	KX200461
Kurixalus odontotarsus	Asia IAA	KX200195	KX201205	KX201355	KX201879	KX202158	KX202305	KX204453	KX207127	KX208626	KX208772		KX209763	KX211327	KX211762	KX200485
Lankanectes corrugatus	India											AY571653				AY948773
Leiopelma hochstetteri	Europe	KX200132	KX201139	KX201290	KX201811	KX202092	KX202239	KX204386	KX207064	KX208560	KX208704	KX208851	KX209694	KX211260	KX211694	KX200418
Lepidobatrachus laevis	Seychelles											DQ679270				KP295530
Lepidobatrachus sp.	Seychelles	KX200111	KX201116	KX201266		KX202069	KX202217	KX204363	KX207043	KX208537	KX208682	KX208829	KX209671	KX211236	KX211671	KX200394
Leptobrachium chapaense	Asia IAA	KX200174	KX201186	KX201335	KX201858	KX202137	KX202283	KX204432	KX207108	KX208605	KX208750	KX208895	KX209741	KX211307	KX211741	KX200463
Leptodactylodon ovatus	Africa	KX200176	KX201188	KX201337	KX201860	KX202139	KX202285	KX204434	KX207110	KX208606	KX208752	KX208897	KX209743	KX211309	KX211743	KX200465
Leptodactylus albilabris	Seychelles	KX200140	KX201150	KX201300	KX201822	KX202103	KX202249	KX204396	KX207075	KX208571	KX208715	KX208861	KX209705	KX211271	KX211705	
Leptodactylus latrans	Seychelles											DQ158343				DQ306492
Leptodactylus melanonotus	Seychelles											AY364224				AY364194
Leptolalax alpinus	Asia IAA	KX200173	KX201185	KX201334	KX201857	KX202136	KX202282	KX204431	KX207107	KX208604	KX208749	KX208894	KX209740	KX211306	KX211740	KX200462
Leptopelis kivuensis	Africa											KM047088				AY364181
Leptopelis parkeri	Africa			KX201338	KX201861	KX202140	KX202286		KX207111	KX208607	KX208753		KX209744	KX211310		KX200466
Leptophryne borbonica	IAA											EF107287				EF107450
Limnodynastes salmini	Australia	KX200177	KX201189	KX201339	KX201862	KX202141	KX202287	KX204435	KX207112	KX208608	KX208754		KX209745	KX211311	KX211744	KX200467
Limnonectes fujianensis	Asia IAA	KX200175	KX201187	KX201336	KX201859	KX202138	KX202284	KX204433	KX207109		KX208751	KX208896	KX209742	KX211308	KX211742	KX200464
Limnonectes laticeps	Asia IAA											AB488960				AB277320
Limnonectes magnus	Asia IAA											JF744593				EF017994
Liophryne schlaginhaufeni	IAA			KX201247	KX201771		KX202200	KX204346		KX208519		KX208813		KX211219		KX200376
Lithodytes lineatus	Seychelles		KX201140		KX201812	KX202093	KX202240	KX204387	KX207065	KX208561	KX208705	KX208852	KX209695	KX211261	KX211695	KX200419
Litoria caerulea	Australia IAA											AY323767				GQ365988
Liurana xizangensis	Asia	KX200213	KX201224	KX201372	KX201898	KX202176	KX202324	KX204471	KX207145	KX208645	KX208791	KX208930	KX209782	KX211346	KX211781	KX200504

Mantella madagascariensis	Madagascar											AB612043				AB612047
Mantophryne lateralis	IAA			KX201245		KX202054	KX202198		KX207025	KX208517	KX208665	KX208811	KX209656	KX211217	KX211653	KX200374
Megophrys nasuta	IAA											AB612063				AB612068
Melanobatrachus indicus	India											EF018053				EF017983
Melanophryniscus stelzneri	Seychelles	KX200086	KX201090	KX201233	KX201757	KX202042	KX202185	KX204334		KX208504	KX208653	KX208800	KX209644	KX211204	KX211640	KX200361
Meristogenys kinabaluensis	IAA											EF088250				AY364176
Mertensophryne micranotis	Africa											EF107330				EF107491
Metaphrynella pollicaris	IAA											AB611927				AB611932
Metaphrynella sundana	IAA											AB611935				EF017973
Micrixalus sp.	India											DQ347216				EF017986
Microhyla annectens	Asia India IAA											AB611940				AB611945
Microhyla heymonsi	Asia India IAA	KX200179	KX201191	KX201341	KX201864		KX202289	KX204437	KX207114	KX208610	KX208756	KX208899	KX209747	KX211313	KX211746	KX200469
Microhyla marmorata	Asia India IAA											AB611948				AB611953
Microhyla okinavensis	Asia India IAA											AB611956				AB611960
Micryletta inornata	Asia											AB611962				AB611966
Mixophyes coggeri	Australia IAA	KX200178	KX201190	KX201340	KX201863	KX202142	KX202288	KX204436	KX207113	KX208609	KX208755	KX208898	KX209746	KX211312	KX211745	KX200468
Myobatrachus gouldii	Australia											AY364226				AY364196
Nannophryne cophotis	Seychelles											DQ158369				DQ306540
Nannophryne variegata	Seychelles											DQ158410				DQ306515
Nasikabatrachus sahyadrensis	India											AY364225				AY364195
Nectophrynoides tornieri	Africa											EF107329				EF107490
Nyctibates corrugatus	Africa	KX200181		KX201342	KX201866	KX202144	KX202291	KX204439	KX207116	KX208612	KX208758	KX208901	KX209749	KX211315	KX211748	KX200471
Nyctibatrachus sp.	India											AY948913				
Nyctimystes kubori	IAA	KX200182	KX201193	KX201343	KX201867	KX202145	KX202292	KX204440	KX207117	KX208613	KX208759	KX208902	KX209750	KX211316	KX211749	KX200472
Nyctimystes pulcher	IAA											AY948941				GQ365993
Nymphargus grandisonae	Seychelles											EU663443				KF534431
Occidozyga laevis	Asia IAA											DQ347254				EF017995
Occidozyga lima	Asia IAA											AB488961				AB488920
Odontobatrachus natator	Africa	KX200188	KX201199	KX201348	KX201873	KX202151	KX202298	KX204446	KX207122	KX208619	KX208765	KX208908	KX209756	KX211322	KX211755	KX200478
Odontophrynus occidentalis	Seychelles	KX200117	KX201123	KX201273	KX201795	KX202076	KX202224	KX204370	KX207050	KX208544	KX208689		KX209678	KX211243	KX211678	KX200401
Odorrana hosii	Asia											EF088259				KR264331
Odorrana schmackeri	Asia	KX200206	KX201217		KX201891	KX202169	KX202317	KX204464	KX207138	KX208638	KX208784	KX208923	KX209775	KX211339	KX211774	KX200497
Ophryophryne microstoma	Asia IAA	KX200184	KX201195		KX201869	KX202147	KX202294	KX204442	KX207119	KX208615	KX208761	KX208904	KX209752	KX211318	KX211751	KX200474
Oreolalax jingdongensis	Asia	KX200183	KX201194	KX201344	KX201868	KX202146	KX202293	KX204441	KX207118	KX208614	KX208760	KX208903	KX209751	KX211317	KX211750	KX200473
Oreophryne sp.	IAA		KX201148	KX201298	KX201820	KX202101	KX202247	KX204394	KX207073	KX208569	KX208713	KX208859	KX209703	KX211269	KX211703	KX200427
Oreophryne sp. B	IAA											EF018046				EF017976
Osteocephalus taurinus	Seychelles	KX200185	KX201196	KX201345	KX201870	KX202148	KX202295	KX204443	KX207120	KX208616	KX208762	KX208905	KX209753	KX211319	KX211752	KX200475
Otophryne pyburni	Seychelles	KX200101	KX201106	KX201253	KX201777		KX202206	KX204351	KX207032	KX208525	KX208671	KX208818	KX209661	KX211224	KX211659	KX200381
Papurana latouchii	Australia	KX200205	KX201216		KX201890	KX202168	KX202316	KX204463	KX207137	KX208637	KX208783	KX208922	KX209774	KX211338	KX211773	KX200496
Paradoxophyla palmata	Madagascar	KX200112	KX201117	KX201267	KX201789	KX202070	KX202218	KX204364	KX207044	KX208538	KX208683	KX208830	KX209672	KX211237	KX211672	KX200395
Pelobates cultripes	America											AY323758				AY364171

Pelobates syriacus	America	KX200124	KX201130	KX201280	KX201802	KX202082	KX202231	KX204377	KX207057	KX208551	KX208696	KX208841	KX209685	KX211250	KX211684	KX200408
Pelodytes ibericus	America	KX200191	KX201202	KX201351	KX201876	KX202154	KX202301	KX204449	KX207124	KX208622	KX208768	KX208911	KX209759	KX211324	KX211758	KX200481
*Pelophylax nigromaculatus**	Asia America															Dryad
Peltophryne lemur	Seychelles											DQ158386				DQ306513
Peltophryne longinasus	Seychelles											JF342384				JF342426
Peltophryne peltocephala	Seychelles	KX200133	KX201141	KX201291	KX201813	KX202094	KX202241	KX204388	KX207066	KX208562	KX208706	KX208853	KX209696	KX211262	KX211696	KX200420
Petropedetes euskircheni	Africa	KX200189	KX201200	KX201349	KX201874	KX202152	KX202299	KX204447		KX208620	KX208766	KX208909	KX209757		KX211756	KX200479
Petropedetes parkeri	Africa											DQ019505				AY364183
Phlyctimantis boulengeri	Africa	KX200186	KX201197	KX201346	KX201871	KX202149	KX202296	KX204444		KX208617	KX208763	KX208906	KX209754	KX211320	KX211753	KX200476
Phrynella pulchra	IAA											AB611969				AB611974
Phrynobatrachus africanus	Africa											KF693602				EF017996
Phrynobatrachus krefftii	Africa											GU457687				EF017997
Phrynobatrachus natalensis	Africa	KX200190	KX201201	KX201350	KX201875	KX202153	KX202300	KX204448	KX207123	KX208621	KX208767	KX208910	KX209758	KX211323	KX211757	KX200480
Phrynoidis aspera	Asia IAA											DQ158356				DQ306503
Phrynoidis juxtaspera	Asia IAA											DQ158385				DQ306542
Phrynomantis microps	Africa	KX200194	KX201204	KX201354	KX201878	KX202157	KX202304	KX204452	KX207126	KX208625	KX208771	KX208913	KX209762	KX211326	KX211761	KX200484
Phrynopus bracki	Seychelles											GQ345281				GQ345186
Phyllobates vittatus	Seychelles											EF107296				EF107459
Phyllomedusa hypochondrialis	Seychelles											AY948929				GQ366014
Phyllomedusa tomopterna	Seychelles		KX201210	KX201360	KX201884	KX202163	KX202310	KX204458		KX208631	KX208777		KX209768	KX211332	KX211767	KX200490
Physalaemus cuvieri	Seychelles	KX200100	KX201105	KX201251	KX201775	KX202058	KX202204	KX204350	KX207030	KX208523	KX208670	KX208817	KX209660	KX211223	KX211658	KX200380
Physalaemus pustulosus	Seychelles	KX200088	KX201092	KX201235	KX201759	KX202044	KX202187	KX204336	KX207017	KX208506	KX208655	KX208802	KX209646	KX211206	KX211642	KX200363
Pipa parva	Seychelles	KX200131	KX201138	KX201289	KX201810	KX202091	KX202238	KX204385		KX208559	KX208703	KX208850	KX209693	KX211259	KX211693	KX200417
Pipa pipa	Seychelles	KX200144	KX201154	KX201304	KX201826	KX202107	KX202253	KX204400	KX207078	KX208575	KX208719	KX208865	KX209709	KX211275	KX211709	KX200432
Platymantis hazelae	IAA											DQ347248				EF017993
Platypelis tuberifera	Madagascar			KX201254				KX204352						KX211225	KX211660	KX200382
Plethodontohyla inguinalis	Madagascar											AB611985				AB611990
Pleurodema somuncurensis	Seychelles	KX200091	KX201096	KX201238	KX201763	KX202048	KX202191	KX204340	KX207021	KX208510	KX208659	KX208806	KX209650	KX211210	KX211646	KX200367
Pleurodema thaul	Seychelles	KX200199	KX201209	KX201359	KX201883	KX202162	KX202309	KX204457	KX207131	KX208630	KX208776		KX209767	KX211331	KX211766	KX200489
Polypedates megacephalus	Asia IAA India	KX200192	KX201203	KX201352	KX201877	KX202155	KX202302	KX204450	KX207125	KX208623	KX208769	KX208912	KX209760	KX211325	KX211759	KX200482
Pristimantis thymelensis	Seychelles	KX200162	KX201173	KX201322	KX201845	KX202124	KX202270	KX204419	KX207095	KX208593	KX208737	KX208883	KX209728	KX211294	KX211728	KX200450
Probreviceps durirostris	Africa											KF990045				KF954752
Proceratophrys boiei	Seychelles	KX200125	KX201131	KX201281	KX201803	KX202083	KX202232	KX204378	KX207058	KX208552	KX208697	KX208842	KX209686	KX211251	KX211685	KX200409
Pseudhymenochirus merlini	Africa	KX200193		KX201353		KX202156	KX202303	KX204451		KX208624	KX208770		KX209761		KX211760	KX200483
Pseudis paradoxa	Seychelles	KX200127	KX201133	KX201283	KX201805	KX202085	KX202234	KX204380	KX207060	KX208554	KX208699	KX208844	KX209688	KX211253	KX211687	KX200411
Pseudophilautus wynaadensis	India											GQ204568				AY364169
Ptychadena cooperi	Africa Madagascar New Zealand											KF380597				KF380104
Ptychadena mascareniensis	Africa Madagascar New Zealand											AY571658				EF017992
Ptychadena oxyrhynchus	Africa Madagascar New Zealand	KX200196	KX201206	KX201356	KX201880	KX202159	KX202306	KX204454	KX207128	KX208627	KX208773	KX208914	KX209764	KX211328	KX211763	KX200486
Pyxicephalus edulis	Africa											KF991338				EF107494
Quasipaa spinosa	Asia	KX200198	KX201208	KX201358	KX201882	KX202161	KX202308	KX204456	KX207130	KX208629	KX208775	KX208916	KX209766	KX211330	KX211765	KX200488

Rana amurensis	Asia America	KX200200	KX201211	KX201361	KX201885		KX202311	KX204459	KX207132	KX208632	KX208778	KX208917	KX209769	KX211333	KX211768	KX200491
Rana berlandieri	Seychelles	KX200102	KX201107	KX201255	KX201778	KX202059	KX202207	KX204353	KX207033	KX208526	KX208672	KX208819	KX209662	KX211226	KX211661	KX200383
Rana catesbeiana	Seychelles											AB612037				AB612041
Rana chensinensis	Asia	KX200202	KX201213	KX201363	KX201887	KX202165	KX202313	KX204461	KX207134	KX208634	KX208780	KX208919	KX209771	KX211335	KX211770	KX200493
Rana draytonii	Seychelles	KX200137	KX201146	KX201296	KX201818	KX202099	KX202245	KX204392	KX207071	KX208567	KX208711	KX208857	KX209701	KX211267	KX211701	KX200425
Rana japonica	Asia											AB728272				KR264277
Rana pipiens	Seychelles											JN227250				JN227141
Rana temporaria	America											AY323776				EF017988
Rana virgatipes	Seychelles	KX200135	KX201144	KX201294	KX201816	KX202097	KX202243	KX204390	KX207069	KX208565	KX208709	KX208855	KX209699	KX211265	KX211699	KX200423
Ranitomeya imitator	Seychelles	KX200083	KX201087	KX201230	KX201754	KX202039	KX202182	KX204332		KX208501	KX208650	KX208797	KX209641	KX211201	KX211636	KX200358
Rentapia hosii	IAA	KX200084	KX201088	KX201231	KX201755	KX202040	KX202183		KX207014	KX208502	KX208651	KX208798	KX209642	KX211202	KX211637	KX200359
Rhacophorus dennysi	Asia IAA India	KX200187	KX201198	KX201347	KX201872	KX202150	KX202297	KX204445	KX207121	KX208618	KX208764	KX208907	KX209755	KX211321	KX211754	KX200477
Rhaebo glaberrimus	Seychelles											DQ158377				DQ306548
Rhaebo nasicus	Seychelles											DQ158396				DQ306512
Rhinella marina	Seychelles	KX200107	KX201112	KX201261	KX201784	KX202064	KX202213	KX204358	KX207038	KX208532	KX208677	KX208824	KX209667	KX211232	KX211666	KX200389
Rhinella ocellata	Seychelles											JN867519				DQ306538
Rhinoderma darwinii	Seychelles	KX200092	KX201097	KX201239	KX201764	KX202049	KX202192	KX204341	KX207022	KX208511	KX208660	KX208807	KX209651	KX211211	KX211647	KX200368
Rhinophrynus dorsalis	Seychelles	KX200203	KX201214	KX201364	KX201888	KX202166	KX202314		KX207135	KX208635	KX208781	KX208920	KX209772	KX211336	KX211771	KX200494
Rulyrana adiazeta	Seychelles											EU663460				KF534442
Rulyrana flavopunctata	Seychelles											EU663467				KF534443
Sachatamia ilex	Seychelles											EU663446				KF534447
Sanguirana luzonensis	IAA											EU076755				KR264350
Scaphiophryne boribory	Madagascar	KX200115	KX201121	KX201271	KX201793	KX202074	KX202222	KX204368	KX207048	KX208542	KX208687	KX208834	KX209676	KX211241	KX211676	KX200399
Scaphiophryne madagascariensis	Madagascar											AB612001				AB612006
Scaphiophryne marmorata	Madagascar	KX200114	KX201119	KX201269	KX201791	KX202072	KX202220	KX204366	KX207046	KX208540	KX208685	KX208832	KX209674	KX211239	KX211674	KX200397
Scaphiopus couchii	Seychelles	KX200208	KX201219	KX201367	KX201893	KX202171	KX202319	KX204466	KX207140	KX208640	KX208786	KX208925	KX209777	KX211341	KX211776	KX200499
Scaphiopus holbrookii	Seychelles											AB612071				AB612076
Schismaderma carens	Africa	KX200096	KX201100	KX201243	KX201768	KX202052	KX202196	KX204344	KX207024	KX208515	KX208663	KX208810	KX209654	KX211215	KX211651	KX200372
Scinax ruber	Seychelles	KX200087	KX201091	KX201234	KX201758	KX202043	KX202186	KX204335	KX207016	KX208505	KX208654	KX208801	KX209645	KX211205	KX211641	KX200362
Sclerophrys brauni	Africa											DQ158361				DQ306514
Sclerophrys maculata	Africa											KJ609678				DQ306533
Scotobleps gabonicus	Africa	KX200209	KX201220	KX201368	KX201894	KX202172	KX202320	KX204467	KX207141	KX208641	KX208787	KX208926	KX209778	KX211342	KX211777	KX200500
Scutiger gongshanensis	Asia	KX200210	KX201221	KX201369	KX201895	KX202173	KX202321	KX204468	KX207142	KX208642	KX208788	KX208927	KX209779	KX211343	KX211778	KX200501
Sooglossus thomasseti	New Zealand	KX200123	KX201129	KX201279	KX201801		KX202230	KX204376	KX207056	KX208550	KX208695	KX208840	KX209684	KX211249	KX211683	KX200407
Spea intermontana	Seychelles	KX200121	KX201127	KX201277	KX201799	KX202080	KX202228	KX204374	KX207054	KX208548	KX208693	KX208839	KX209682	KX211247	KX211681	KX200405
Spea multiplicata	Seychelles	KX200212	KX201223	KX201371	KX201897	KX202175	KX202323	KX204470	KX207144	KX208644	KX208790	KX208929	KX209781	KX211345	KX211780	KX200503
Spelaeophryne methneri	Africa											KF990042				EF107453
Staurois latopalmatus	IAA											AB612049				EF017987
Stefania ginesi	Seychelles											DQ679308				GQ345178
Stereocyclops incrassatus	Seychelles			KX201287		KX202089						KX208848		KX211257	KX211691	KX200415
Strabomantis biporcatus	Seychelles											GQ345283				GQ345188

Strabomantis sulcatus	Seychelles	KX200082	KX201086	KX201229	KX201753	KX202038	KX202181	KX204331	KX207013	KX208500	KX208649	KX208796	KX209640	KX211200	KX211635	KX200357
Strongylopus grayii	Africa	KX200211	KX201222	KX201370	KX201896	KX202174	KX202322	KX204469	KX207143	KX208643	KX208789	KX208928	KX209780	KX211344	KX211779	KX200502
Stumpffia pygmaea	Madagascar			KX201257	KX201780		KX202209			KX208528		KX208821		KX211228		KX200385
Sylvirana guentheri	Asia	KX200204	KX201215	KX201365	KX201889	KX202167	KX202315	KX204462	KX207136	KX208636	KX208782	KX208921	KX209773	KX211337	KX211772	KX200495
Synapturanus sp.	Seychelles											EF018051				EF017981
Telmatobius vellardi	Seychelles	KX200215	KX201226	KX201374	KX201900	KX202178	KX202326	KX204473	KX207147	KX208646	KX208793	KX208932	KX209784	KX211348	KX211783	KX200506
Teratohyla spinosa	Seychelles											EU663483				KF534452
Thoropa taophora	Seychelles											GQ345288				GQ345193
Trachycephalus typhonius	Seychelles											EU034147				AY364185
Trichobatrachus robustus	Africa	KX200214	KX201225	KX201373	KX201899	KX202177	KX202325	KX204472	KX207146		KX208792	KX208931	KX209783	KX211347	KX211782	KX200505
Uperodon montanus	India											AB611993				AB611998
Uperodon systoma	India											EF018049				EF017979
Uperodon variegatus	India											EF018052				EF017982
Uperoleia laevigata	Australia IAA											KJ874616				EF107474
Vitreorana helenae	Seychelles											EU663471				KF534456
Xenophrys omeimontis	Asia IAA	KX200180	KX201192		KX201865	KX202143	KX202290	KX204438	KX207115	KX208611	KX208757	KX208900	KX209748	KX211314	KX211747	KX200470
Xenopus epitropicalis	Africa	KX200216	KX201227	KX201375	KX201901	KX202179	KX202327	KX204474	KX207148	KX208647	KX208794	KX208933	KX209785	KX211349	KX211784	KX200507
Xenopus kobeli	Africa	KX200217	KX201228		KX201902	KX202180	KX202328	KX204475	KX207149	KX208648	KX208795	KX208934	KX209786	KX211350		KX200508
Xenorhina obesa	IAA											EF018048				EF017978
Xenorhina sp.	IAA	KX200129	KX201136	KX201286	KX201808	KX202088	KX202236	KX204383	KX207062	KX208557	KX208702	KX208847	KX209691	KX211256	KX211690	KX200414

* See original article for data on these taxa and for data on the outgroups

The data used in these analyses were taken from Feng et al. (2017), who used multiple sources for a large amount of data. In order to reduce the dataset, we selected the 20 most parsimony informative markers and, from those, we eliminated the ones with the least complete dataset, which reduced our selection to 14 markers: CILP, DISP2, DMXL1, DSEL, EVPL, EXOC8, HYP, MSH6, PPL, RAG1, RAG2, SALL1, WFIKKN2, ZFPM2. Since this was inbetween a genus-level and a family-level phylogeny, we attributed distributions according to the range of the entire genus. In addition, we extrapolated the dates from the original articles with soft lower boundaries, because we reduced the amount of data used, even though other articles have retrieved the same topology (e.g. Li et al., 2013) with congruent dates.

9. Emballonurinae

Table S8: Genbank numbers for the sequences used in the Emballonuridae analyses with the distribution attributed to each terminal.

Species	Locality	CytB	RAG2
Emballonura tiavato	Madagascar	DQ178278	
Emballonura tiavato	Madagascar	DQ178275	
Emballonura tiavato	Madagascar	DQ178280	HQ693737
Emballonura tiavato	Madagascar	DQ178277	
Emballonura tiavato	Madagascar	DQ178252	
Emballonura tiavato	Madagascar	DQ178255	HQ693738
Emballonura tiavato	Madagascar	DQ178253	
Emballonura tiavato	Madagascar	DQ178250	
Emballonura tiavato	Madagascar	DQ178273	
Emballonura tiavato	Madagascar	DQ178262	
Emballonura tiavato	Madagascar	DQ178264	
Emballonura tiavato	Madagascar	DQ178265	
Emballonura tiavato	Madagascar	DQ178249	
Emballonura tiavato	Madagascar	DQ178272	
Emballonura atrata	Madagascar	HQ693729	
Emballonura atrata	Madagascar	HQ693732	
Emballonura atrata	Madagascar	HQ693733	
Emballonura atrata	Madagascar	HQ693730	
Emballonura atrata	Madagascar	HQ693726	
Emballonura atrata	Madagascar	HQ693734	HQ693745
Emballonura atrata	Madagascar	HQ693727	HQ693739
Emballonura atrata	Madagascar	HQ693728	
Emballonura atrata	Madagascar	HQ693725	

Species	Locality	CytB	RAG2
Emballonura atrata	Madagascar	HQ693731	
Emballonura monticola	IAA	HQ693714	HQ693747
Emballonura monticola	IAA	HQ693715	HQ693746
Emballonura semicaudata	IAA	HQ693708	HQ693751
Emballonura semicaudata	IAA	HQ693709	HQ693751
Emballonura alecto	IAA	HQ693710	HQ693752
Emballonura alecto	IAA	HQ693711	HQ693748
Emballonura dianae	Australia	HQ693716	HQ693750
Emballonura dianae	Australia	HQ693717	HQ693750
Emballonura raffrayana	Australia	EF584224	
Emballonura beccarii	Australia	EF584222	
Emballonura beccarii	Australia	EF635537	
Emballonura serii	Australia	EF635544	
Emballonura serii	Australia	EF635543	
Mosia nigrescens	Australia	EF635559	
Coleura afra	Africa	HQ693720	HQ693743
Coleura seychellensis	Seychelles	HQ693735	
Peropteryx kappleri	America	HQ693719	HQ693741
Saccopteryx leptura	America	HQ693718	HQ693740
Cormura brevirostris	America	EF584158	HQ693742
Taphozous nudiventris	Iran	HQ693712	HQ693749
Nycteris hispida	Africa	HQ693722	HQ693736

10. Strepsirrhini

The sequences used in the Strepsirrhini analyses were downloaded directly from the original article's dryad repository (https://datadryad.org/resource/doi:10.5061/dryad.51f00) and re-aligned. All calibration points were set to lognormal with parameters that we set to retrieve the dates from the original fossil descriptions: *Aegyptopithecus* (M = 1.0 and S = 0.6; offset = 29.9 m.y.a. (Seiffert, 2006; Simons, 1965)); *Branisella* (M = 1.0 and S = 0.59; offset = 26.4 m.y.a. (Hoffstetter, 1969; Takai, Anaya, Shigehara, & Setoguchi, 2000)); *Djebelemur* (M = 1.0 and S = 0.86; offset = 41.3 m.y.a. (Godinot, 2006; Hartenberger & Marandat, 1992)); *Nycticeboides* (M = 1.0 and S = 0.83; offset = 6.4 m.y.a. (MacPhee & Jacobs, 1986)); *Plesiopithecus* (M = 1.0 and S = 0.84; offset = 33.9 m.y.a. (E. L. Simons, 1992)); *Saharagalago* (M = 1.0 and S = 1.07; offset = 33.9 m.y.a. (Seiffert, Simons, & Attia, 2003)); and *Wadilemur* (M = 1.0 and S = 0.84; offset = 33.9 m.y.a. (Seiffert, Simons, Ryan, & Attia, 2005; Simons, 1997)). The dates we obtained are older than in Herrera and Dávalos (2016), but the topology was identical except for the position of

Phaner furcifer, which in our analyses was in agreement with Masters, Silvestro, Génin, and DelPero (2013).

11. Pteropodidae

Table S9: Genbank numbers for the sequences used in the Pteropodidae analyses with the distribution attributed to each terminal.

Species	Locality	CytB	12S	16S	RAG1	RAG2	vWF	BRCA1
Acerodon celebensis	IAA	GQ410231	JN398167	AF293641	EU617946	EU617896	EU617928	JN398239
Aethalops alecto	IAA	GQ410218	GQ410312	GQ410335	GQ410263	GQ410240	GQ410286	JN398260
Aethalops alecto	IAA	GQ410219	GQ410313	GQ410336	GQ410264	GQ410241	GQ410287	
Alionycteris paucidentata	IAA	GQ410221	GQ410315	GQ410338	GQ410266	GQ410243	GQ410289	
Alionycteris paucidentata	IAA	GQ410222	GQ410316	GQ410339	GQ410267	GQ410244	GQ410290	JN398248
Aproteles bulmerae	Australia		U93066	AF293645				
Balionycteris maculata	IAA	GQ410227	GQ410321	GQ410344	GQ410272	GQ410249	GQ410295	JN398257
Boneia bidens	IAA	FJ218481	FJ218475	FJ218475	FJ218468	FJ218464	FJ218471	
Casinycteris argynnis	Africa	JN398197	JN398168	JN398168	JN398284	JN398301	JN398268	JN398264
Chironax melanocephalus	IAA	GQ410220	GQ410314		GQ410265	GQ410242	GQ410288	JN398256
Cynopterus brachyotis	IAA	AB046321	U93068	U93068				
Cynopterus brachyotis	IAA	GQ410210	GQ410303	GQ410327	GQ410256	GQ410233	GQ410279	JN398258
Cynopterus sphinx	IAA	DQ445703	GQ410302	GQ410326	EU617947	EU617897	DQ445697	KT875821
Desmalopex leucopterus	IAA	JN398198	JN398169	JN398169	EU617966	EU617915	EU617929	JN398252
Dobsonia inermis	Australia	DQ445704	FJ218476	FJ218476	EU617948	EU617898	DQ445686	JN398234
Dobsonia minor	Australia	DQ445705	FJ218477	FJ218477	FJ218467	FJ218463	DQ445701	
Dobsonia moluccensis	Australia	FJ218484	JN398196	JN398196	EU617949	EU617899	EU617930	JN398220
Dobsonia praedatrix	Australia	JN398199	JN398170	JN398170	JN398285	JN398302	JN398269	JN398222
Dyacopterus spadiceus	IAA	GQ410230	GQ410324	GQ410347	GQ410275	GQ410252	GQ410298	JN398259
Eidolon helvum	Africa	JN398200	JN398171	JN398171	EU617950	EU617900	EU617931	JN398240
Eonycteris robusta	IAA	JN398201	JN398172	JN398172	JN398286	JN398303	JN398270	JN398251
Eonycteris spelaea	IAA	AB062476	U93059					
Eonycteris spelaea	IAA	FJ218482	FJ529123	FJ529123	EU617951	EU617901	DQ445684	JN398254
Epomophorus wahlbergi	Africa	DQ445706	JN398174	JN398174	EU617953	EU617903	DQ445691	JN398266
Epomops franqueti	Africa	DQ445707	KT875876	KT875876	EU617952	EU617902	DQ445692	
Epomops franqueti	Africa	JN398202	JN398175	JN398175	JN398287	JN398304	JN398271	JN398233
Haplonycteris fischeri	IAA	GQ410225	GQ410319	GQ410342	GQ410270	GQ410247	GQ410293	JN398245
Haplonycteris fischeri	IAA	GQ410226	GQ410320	GQ410343	GQ410271	GQ410248	GQ410294	
Harpyionycteris celebensis	IAA	JN398203	FJ218473	FJ218473	JN398288			JN398225
Harpyionycteris whiteheadi	IAA	DQ445708	FJ218474	FJ218474	EU617954	EU617904	DQ445690	JN398246
Hypsignathus monstrosus	Africa	JN398204	JN398176	JN398176	JN398289	JN398305	JN398272	JN398235
Latidens salimalii	India	GQ410217	GQ410311	FJ009216				
Lissonycteris angolensis	Africa	JN398205	JN398177	JN398177	JN398290	JN398306	JN398273	JN398243
Macroglossus minimus	Australia	JN398206	JN398178	JN398178	EU617955	EU617905	DQ445693	JN398229
Macroglossus sobrinus	IAA		JN398179	JN398179	JN398291	JN398307	JN398274	JN398236

Megaerops ecaudatus	IAA	GQ410214	GQ410308	GQ410332	GQ410260	GQ410237	GQ410283	JN398262
Megaerops kusnotoi	IAA	GQ410215	GQ410309	GQ410333	GQ410261	GQ410238	GQ410284	JN398255
Megaerops wetmorei	IAA	GQ410212	GQ410306	GQ410330	GQ410258	GQ410235	GQ410281	
Megaerops wetmorei	IAA	GQ410213	GQ410307	GQ410331	GQ410259	GQ410232	GQ410282	
Megaloglossus woermanni	Africa	DQ445710	JN398180	JN398180	EU617956	EU617906	DQ445702	JN398231
Melonycteris fardoulisi	Australia	FJ218478	JN398181	JN398181	EU617957	EU617907	DQ445699	JN398237
Melonycteris melanops	Australia	JN398207	JN398182	JN398182	FJ218465	FJ218461	FJ218469	JN398223
Micropteropus pusillus	Africa	JN398208	JN398183	JN398183	JN398292	JN398308	JN398275	JN398241
Myonycteris torquata	Africa	FJ218483	JN398184	JN398184	EU617958	EU617908	DQ445700	JN398232
Nanonycteris veldkampii	Africa	JN398209	JN398185	JN398185	JN398293	JN398309	JN398276	JN398253
Nyctimene cephalotes	IAA	JN398210	JN398186	JN398186	JN398294		JN398277	JN398226
Nyctimene robinsoni	Australia	AF144066	GQ410325	GQ410348	GQ410276	GQ410253	GQ410299	JN398221
Nyctimene vizcaccia	Australia	DQ445711	JN398187	JN398187	EU617959	EU617904	DQ445698	JN398238
Otopteropus cartilagonodus	IAA	GQ410223	GQ410317	GQ410340	GQ410268	GQ410245	GQ410291	
Otopteropus cartilagonodus	IAA	GQ410224	GQ410318	GQ410341	GQ410269	GQ410246	GQ410292	JN398249
Penthetor lucasi	IAA	GQ410216	GQ410310	GQ410334	GQ410262	GQ410239	GQ410285	JN398261
Ptenochirus jagori	IAA	FJ218480	GQ410304	GQ410328	EU617960	EU617910	DQ445696	JN398250
Ptenochirus jagori	IAA	GQ410211	GQ410305	GQ410329	GQ410257	GQ410234	GQ410280	
Pteralopex atrata	Australia		U93069	AF293643				
Pteralopex acrodonta	Pacific	FJ561376	FJ588908					
Pteropus admiralitatum	Australia	KJ532441	U93072					
Pteropus aldabrensis	Seychelles	FJ561394	FJ588896					
Pteropus conspicillatus	Australia	FJ561378	FJ588879		EU617963			
Pteropus conspicillatus	Australia	FJ561379	FJ588880					
Pteropus conspicillatus	Australia	FJ561380	FJ588881					
Pteropus dasymallus	IAA	AB042770	AB042770	AB042770				
Pteropus giganteus	India	FJ561381	FJ588882		JN414929	AY011964		
Pteropus giganteus	India	JN398211	AY012138	AY011170	EU617964	EU617913	EU617935	JN398242
Pteropus hypomelanus	IAA	FJ561382	FJ588883	AF044623	EU617965	EU617914	AF203777	
Pteropus hypomelanus	IAA	FJ561383	FJ588884	AF069537		AY141025	DQ445687	
Pteropus livingstonii	Comoros	FJ561384	FJ588885					
Pteropus niger	MI	FJ561385	FJ588886					
Pteropus poliocephalus	Australia	FJ561386	FJ588887		EU617971	EU617920	EU617940	
Pteropus poliocephalus	Australia	FJ561387	FJ588888					
Pteropus pumilus	IAA	FJ561388	FJ588889		EU617972	EU617921	EU617941	
Pteropus pumilus	IAA	FJ561389	FJ588890					
Pteropus pumilus	IAA	FJ561390	FJ588891					
Pteropus rodricensis	MI	FJ561391	FJ588892	AF044624				
Pteropus rodricensis	MI	FJ561392	FJ588893					
Pteropus rufus	Madagascar	FJ561393	FJ588894					
Pteropus scapulatus	Australia	AF321050	AF321050	AF321050	EU617974			
Pteropus scapulatus	Australia	FJ561377	FJ588895					
Pteropus seychellensis comoroensis	Comoros.	FJ561395	FJ588897					

Pteropus seychellensis comoroensis	Comoros	FJ561396	FJ588898					
Pteropus seychellensis comoroensis	Comoros	FJ561397	FJ588899					
Pteropus seychellensis comoroensis	Comoros	FJ561398	FJ588900					
Pteropus seychellensis seychellensis	Seychelles	FJ561399	FJ588901					
Pteropus seychellensis seychellensis	Seychelles	FJ561400	FJ588902					
Pteropus speciosus	IAA	AB062474						
Pteropus tonganus	Pacific	JN398213	JN398188	JN398188	EU617976	EU617924	DQ445695	JN398267
Pteropus vampyrus	IAA	EF584230	KJ532340		EU617977	EU617925	EU617944	
Pteropus vampyrus	IAA	FJ561402	FJ588904	NC_026542				
Pteropus vampyrus	IAA	FJ561403	FJ588905	KP214033				
Pteropus vampyrus	IAA	JN398212	JN398189	JN398189	JN398295	JN398310	JN398278	JN398228
Pteropus voeltzkowi	Africa	FJ561404	FJ588906					
Pteropus voeltzkowi	Africa	FJ561405	FJ588907					
Rousettus aegyptiacus	Africa	DQ445713	AB205183	AB205183	EU617979		DQ445688	KT875815
Rousettus aegyptiacus	Africa	DQ445714	AB205183	AB205183	KT875845		DQ445694	
Rousettus amplexicaudatus	IAA	AB046329	U93070	AF203742	AF447512	AF447529	AY057836	AF447500
Rousettus leschenaultii	India	JN398218	JN398190	JN398190	JN398300	JN398313	JN398283	JN398219
Rousettus madagascariensis	Madagascar	JN398214	JN398191	JN398191	JN398296	JN398311	JN398279	JN398224
Scotonycteris zenkeri	Africa	JN398216	JN398192	JN398192	JN398297	JN398312	JN398280	JN398265
Sphaerias blanfordi	Asia	GQ410228	GQ410322	GQ410345	GQ410273	GQ410250	GQ410296	
Sphaerias blanfordi	Asia	GQ410229	GQ410323	GQ410346	GQ410274	GQ410251	GQ410297	JN398230
Stenonycteris lanosus	Africa	JN398215	JN398193	JN398193	JN398298	JN400925	JN398281	JN398244
Styloctenium mindorensis	IAA	JN398217	JN398194	JN398194	JN398299		JN398282	JN398227
Syconycteris australis	Australia	FJ218479	JN398195	JN398195	FJ218466	FJ218462	FJ218470	JN398247
Syconycteris australis	Australia	GQ410232	U93060	AF293650	GQ410278	GQ410255	GQ410301	
Thoopterus nigrescens	IAA	KC747699	U93067	AF293646				
Megaderma lyra	IAA	DQ888678	AF069538	AF069538	AF203757	AF203767	U31616	AF203749
Megaderma lyra	India	DQ888678	AF069538	AF069538	AF203757	AF203767	U31616	AF203749
Rhinopoma hardwickii	India	AY629005	AF263232	AF263232	AF447518	AF447535	AF447551	AF447504
Rhinopoma hardwickii	Africa	AY629006	AF263232	AF263232	AF447518	AF447535	AF447551	AF447504
Hipposideros commersoni	Madagascar	KR606333	AY395856	AY395856	AF203760	AF203770	AF203778	AF203752
Artibeus jamaicensis	America	AF061340	AF061340	AF061340	AY834655	AY834663	AY834737	AY834647
Rhinolophus creaghi	IAA	EF108163			AF447511	AF447528	AF447546	AF447499

Since we did not have a solid calibration point for this family without including a considerable number of microchiropteran terminals, we used the dates from Agnarsson, Zambrana-Torrelio, Flores-Saldana, and May-Collado (2011): a root prior with lognormal distribution (M = 4.075 and S = 0.05) and a Pteropodidae-Rhinolophoidea prior with lognormal distribution (M = 3.989 and S = 0.09).

12. Boidae

Table S10: Genbank numbers for the sequences used in the Boidae analyses with the distribution attributed to each terminal.

Species	Locality	CytB	CMOS	NT3	BDNF	RAG1	ODC
Acrantophis dumerili	Madagascar MI	U69735	AY099963	AY988049	AY988032	AY988066	DQ465533
Acrochordus granulatus	IAA	AF217841	AF471124	EU390905	EU402621	EU402831	
Acrochordus javanicus	IAA Australia	KX694897	HM234058	AY988053	AY988036	AY988070	DQ465538
Anilius scytale	America	U69738	AY099965	AY988055	AY988038	AY988072	DQ465540
Aspidites melanocephala	Australia	U69741	DQ465557	DQ465558	DQ465559	DQ465560	DQ465552
Boa constrictor	America	U69740	AF471115	AY988047	AY988030	AY988064	DQ465531
Calabaria reinhardtii	Africa	AY099985	AY099978	AY988058	AY988041	AY988075	DQ465542
Candoia carinata	IAA	AY099984	AY099961	AY988048	AY988031	AY988065	DQ465532
Candoia aspera	IAA Australia	U69751					
Charina bottae	America	AY099986	AY099971	AY988059	AY988042	AY988076	DQ465543
Corallus caninus	America	U69763	AY987964	AY988044	AY988027	AY988061	DQ465528
Cylindrophis ruffus	IAA	AF471032	AF471133	AY988054	AY988037	AY988071	DQ465539
Epicrates cenchria	America	U69777	AY099966	AY988045	AY988028	AY988062	DQ465529
Epicrates striatus	America	U69799	DQ465553	DQ465554	DQ465555	DQ465556	DQ465551
Gongylophis colubrinus	Africa	U69812	DQ465568	DQ465569	DQ465570	DQ465571	DQ465550
Gongylophis conicus	India	GQ225658	DQ469787	AY988057	AY988040	AY988074	DQ465547
Eryx elegans	Asia	U69818					
Eryx jayakari	Arabic Peninsula		DQ465565		DQ465566	DQ465567	DQ465548
Eryx johnii	India	U69823	AY099975	DQ465575	DQ465576	DQ465577	DQ465546
Eunectes notaeus	America	U69810	AY099964	AY988046	AY988029	AY988063	DQ465530
Exiliboa placata	America	AY099989	AY099973	AY988051	AY988034	AY988068	DQ465535
Lichanura trivirgata	America	U69844	AY099974	DQ465578	DQ465579	DQ465580	DQ465536
Loxocemus bicolor	America	AY099993	AY099969	DQ465572	DQ465573	AY444061	DQ465545
Morelia spilota	Australia	U69851	AF544723	AY988052	AY988035	AY988069	DQ465537
Ramphotyphlops braminus	America	AY099990	AY099980	AY988060	AY988043	AY988077	DQ465544
Sanzinia madagascariensis	Madagascar	U69866	AY099982	AY988050	AY988033	AY988067	DQ465534
Tropidophis haetianus	America	U69868	AY099962	AY988056	AY988039	AY988073	DQ465541
Xenopeltis unicolor	IAA	AY121369	DQ465561	DQ465562	DQ465563	DQ465564	DQ465549

Some of the calibration points used by the original article have been updated by recent discoveries. We set the following calibration points with lognormal distribution: *Coniophis* (M = 1.0 and S = 1.59; offset = 83.0 m.y.a. (Gómez, Báez, & Rougier, 2008; Marsh, 1892)); *Charina prebottae* (M = 1.0 and S = 0.8; offset = 13.6 m.y.a. (Brattstrom, 1958)); *Cheilophis* (M = 1.0 and S = 0.715; offset = 55.0 m.y.a. (Miller, 1955; Rage, 1984)); *Dunnophis* (M = 0.8 and S = 0.585; offset = 59.0 m.y.a. (Estes, 1976; Szyndlar, Smith, & Rage, 2008)); *Helagras* (M = 1.2 and S = 0.5; offset = 63.3 m.y.a. (Sullivan & Lucas, 1986)); *Dinilysia* (M = 1.25 and S = 1.42; offset = 89.0 m.y.a. (Caldwell & Albino, 2003)); *Pseudoepicrates* (M = 1.0 and S = 0.94; offset = 15.97 m.y.a. (Onary & Hsiou, 2018)); and Nigerophiidae (M = 1.5 and S = 1.5; offset = 66.0 m.y.a. (Laduke, Krause, Scanlon, & Kley, 2010)).

13. Chamaeleoninae

The sequences used in the Chamaeleoninae analyses were downloaded directly from the original article's dryad repository (https://datadryad.org/resource/doi:10.5061/dryad.11350). All calibration points were identical to those of the original article.

14. Gekkonidae

Table S11: Genbank numbers for the sequences used in the Gekkonidae analyses with the distribution attributed to each terminal.

Species	Location	12S	CMOS	CytB	ND2	PDC	RAG1	RAG2
Afroedura karroica	Africa		JQ945523		JX041302	JQ945345	JQ945277	JQ945415
Afroedura multiporis	Africa				EU054232	EU054184	EU054208	
Afroedura pondolia	Africa				EU054231		EU054207	
Afrogecko porphyreus	Africa	DQ852696	AY172919	EF490750	EF490776	EF490697	EF490723	JQ945418
Afrogekko plumicaudus	Africa		JQ945525		JX041304	JQ945347	JQ945279	JQ945417
Afrogecko swartbergensis	Africa		JQ945527		JX041305	JQ945348	JQ945280	JQ945419
Agamura persica	Asia	DQ852726	DQ852728	EU589176	JX041306	JQ945349	JQ945281	JQ945420
Ailuronyx seychellensis	Seychelles	AY221267	AY221331	FJ830052			KY037922	
Ailuronyx tachyscopaeus	Seychelles		JQ945529		JX041307	JQ945350	JQ945282	JQ945421
Ailuronyx trachygaster	Seychelles		JQ945530		JX041308	JQ945351	JQ945283	JQ945422
Alsophylax pipiens	Asia		JQ945531		JX041309	JQ945352	JQ945284	JQ945423
Blaesodactylus antongilensis	Madagascar		JQ945534		EU054254	EU054206	EU054230	JQ945426
Blaesodactylus boivini	Madagascar	EU596607			EU054252	EU054204	EU054228	
Blaesodactylus sakalava	Madagascar				EU054251	EU054203	EU054227	
Bunopus crassicauda	Arabic Peninsula	EU589154		EU589177				
Bunopus tuberculatus	Arabic Peninsula	EU589160	AF148706	EU589181	HQ443541	JQ945355	JQ945287	JQ945427
Calodactylodes aureus	India	DQ852697	AY172921					
Calodactylodes illingworthorum	India		JQ945536		JX041318	JQ945356	JQ945288	JQ945428
Chondrodactylus angulifer	Africa	DQ275403	JQ945537	AY123394	JX041320	JQ945357	JQ073242	JQ945429
Chondrodactylus bibronii	Africa		EU293690			EU293712	EU293645	EU293735
Chondrodactylus fitzsimonsi	Africa	DQ275404		AF449125	JN393945		DQ275448	
Chondrodactylus turneri	Africa	DQ852708	AY172938	AF449124	KY224249	KM073612	KM073525	
Christinus alexanderi	Australia				KF666813		KF666897	KF666856
Christinus guentheri	Australia				KF666800		KF666887	KF666846
Christinus marmoratus	Australia	DQ852698	FJ855461		JX041322	JQ945358	FJ855440	JQ945430
Cnemaspis africana	Africa		JQ945539		JX041323	JQ945359	JQ945291	JQ945431
Cnemaspis dickersonae	Africa		JQ945540		JX041324	JQ945360	JQ945292	JQ945432
Cnemaspis kandiana	India		JQ945541		JX041325	JQ945361	JQ945293	JQ945433
Cnemaspis kendallii	IAA		AY172923		JX041326	JQ945362	JQ945294	JQ945434
Cnemaspis limi	IAA		EF534935		JX041327	EF534851	EF534809	EF534977
Cnemaspis podihuna	India		JQ945543		JX041328	JQ945363	JQ945295	JQ945435
Cnemaspis tropidogaster	India	DQ852716	DQ852729					

Cnemaspis uzungwae	Africa		JQ945544		JX041329	JQ945364	JQ945296	JQ945436
Colopus kochii	Africa		JQ945545	AY123398	JX041336	JQ945365	DQ275418	JQ945437
Colopus wahlbergii	Africa	DQ275375	JQ945546	AY123395	JX041337	JQ945366	DQ275419	JQ945438
Crossobamon orientalis	Asia	DQ852715	DQ852730	HM921200	HQ443529	JQ945368	JQ945299	JQ945440
Cryptactites peringueyi	Africa	DQ852718	DQ852731		JX041339	JQ945369	JQ945300	JQ945441
Cyrtodactylus angularis	Australia IAA India Asia		JQ945549		HQ401212	JQ945370	JQ945301	JQ945442
Cyrtodactylus ayeyarwadyensis	Australia IAA India Asia		JQ945550	EU268380	EU268348	EU268317		JQ945443
Cyrtodactylus consobrinus	Australia IAA India Asia			EU268381	EU268349	EU268318		
Cyrtodactylus irregularis	Australia IAA India Asia		JQ945551		JX041341	JQ945371	JQ945302	JQ945444
Cyrtodactylus jarujini	Australia IAA India Asia		JQ945552		HQ401213	JQ945372	JQ945303	JQ945445
Cyrtodactylus loriae	Australia IAA India Asia			EU268382	EU268350	EU268319		
Cyrtodactylus novaeguineae	Australia IAA India Asia		HQ426531		HQ401210	HQ426185	HQ426274	HQ426447
Cyrtodactylus philippinicus	Australia IAA India Asia		JQ945553		JX041344	JQ945373	JQ945304	JQ945446
Cyrtopodion agamuroides	Asia	EU589161		EU589185				
Cyrtopodion caspium	Asia	EU589163	JQ945620	EU589187	JX041448	JQ945409	JQ945340	JQ945514
Cyrtopodion longipes	Asia	EU589170	JQ945621	EU589193	JX041449	JQ945410	JQ945341	JQ945515
Cyrtopodion russowii	Asia		JQ945588		JX041384	JQ945383	JQ945315	JQ945481
Cyrtopodion scabrum	Asia	EU589172	HQ426532	EU589195	JX041345	HQ426186	HQ426275	HQ426448
Cyrtopodion spinicaudum	Asia		JQ945589		JX041385	JQ945384	JQ945316	JQ945482
Delma butleri	Australia		AY134548		AY134584	HQ426187	KP851280	HQ426449
Dixonius siamensis	IAA		JQ945557		EU054299	EU054267	EU054283	JQ945450
Dixonius vietnamensis	IAA		JQ945558		EU054298	EU054266	EU054282	JQ945451
Ebenavia inunguis	Africa Comoros Madagascar MI	EU596608	FJ830144	FJ830053	EF536191	HQ426191	EF536143	HQ426453
Elasmodactylus tetensis	Africa	DQ275407	JQ945559	AY026926	JX041349	JQ945376	DQ275451	JQ945452
Elasmodactylus tuberculosus	Africa	DQ275408		AF449134			DQ275452	
Geckolepis maculata	Comoros Madagascar	EU596610	JQ945562		EU054235	EU054187	EU054211	JQ945455
Geckolepis typica	Madagascar				EU054233	EU054185	EU054209	
Geckonia chazaliae	Africa	AF363574	AF363556	AF364326	JX041443	EU293705	EU293638	JQ301343
Gehyra australis	Australia		JQ945563		GQ257759	JN019113		JQ945456
Gehyra barea	IAA				JN393915	JN393993	JN393960	
Gehyra brevipalmata	IAA				JN393910	JN393987	JN393955	
Gehyra dubia	Australia		JQ945565		GQ257788	JN393989		JQ945458
Gehyra marginata	IAA				JN393931	JN394009	JN393975	
Gehyra mutilata	IAA	DQ852699	FJ830146	FJ830054	GQ257784	JN019114	FJ830237	FJ830328
Gehyra nana	Australia		JQ945567		GQ257760	JN393998		JQ945460
Gehyra oceanica	Pacific				GQ257785	JN394001		
Gehyra variegata	Australia	AF090185	AF090851		AY369026	JN393994		JQ945461
Gehyra xenopus	Australia				GQ257790	JN394010		
Gekko athymus	IAA				JN019075	JN019107	JN019139	
Gekko auriverrucosus	Asia			EU417695	JN019062	JN019096	JN019127	
Gekko badenii	IAA		JQ945569		JN019065	JN019099	JN019130	JQ945462
Gekko chinensis	Asia		JQ945571	EU417676	JN019058	JN019092	JN019123	JQ945464
Gekko gecko	Asia Pacific	HM370130	EU366455	NC_007627	AY282753	JN019087	AY662625	EF534981

Gekko grossmanni	IAA				JN019064	JN019098	JN019129	
Gekko hokouensis	Asia	AF323511		EU417691	JN019060	JN019094	JN019125	
Gekko japonicus	Asia	AF318271		EU417688	JN019059	JN019093	JN019124	
Gekko mindorensis	IAA		JQ945572		FJ487887	JN019108	JN019140	JQ945465
Gekko monarchus	IAA		JQ945573		FJ487870	JN019110	JN019141	JQ945466
Gekko petricolus	IAA				JN019067	JN019101	JN019132	
Gekko smithii	Asia IAA				FJ487868	JN019091	JN019121	
Gekko swinhonis	Asia	AF323518		EU417702	JN019061	JN019095	JN019126	
Gekko vittatus	IAA	NC_008772	JQ945575	NC_008772	AB178897	JN019106	JN019137	JQ945468
Goggia lineata	Africa	AY763261	AY172930		JX041353	JQ945378	JQ945310	JQ945469
Hemidactylus aaronbaueri	India	HM595676		HM595641		HM622367		
Hemidactylus angulatus	Africa	EF202130	HQ426540	DQ120237	EU268367	EU268336	HM559686	
Hemidactylus bouvieri	Africa	DQ120424	AY863042	DQ120249				EF540746
Hemidactylus bowringii	Asia	AF323515		EU268405	EU268374	EU268343		
Hemidactylus brasilianus	America		HQ426523	DQ120257	EU268351	EU268320		HQ426439
Hemidactylus depressus	India			HM559594	HM559626	HM559659	HM559692	
Hemidactylus dracaenacolus	Arabic Peninsula	DQ120380		DQ120209				
Hemidactylus fasciatus	Africa	DQ852724	JQ945577	EU268403	EU268371	EU268340	JQ945311	JQ945470
Hemidactylus flaviviridis	Africa Arabic Peninsula Asia	DQ120454	HQ426541	EU268388	EU268356	EU268325	HM559694	HQ426458
Hemidactylus forbesii	Arabic Peninsula	DQ120339		DQ120168				
Hemidactylus foudaii	Africa	DQ120385		DQ120214				
Hemidactylus frenatus	Australia IAA India Asia	NC_012902	EF534940	EU116513	EU268359	EU268328	EF534814	EF534982
Hemidactylus garnotii	Australia IAA	DQ120459		EU268395	EU268364	EU268333	HM559697	
Hemidactylus giganteus	India	HM595692		HM595658		HM559665		
Hemidactylus gracilis	India	HM595696		HM595660	EU268379	HM622374		
Hemidactylus granti	Arabic Peninsula	DQ120381		DQ120210				
Hemidactylus greefii	Africa	DQ120413	AY863044	EU268401	EU268369	EU268338		HQ426459
Hemidactylus karenorum	IAA	DQ120464		EU268394	EU268362	EU268331		
Hemidactylus leschenaultii	India	HM595697		HM595662		HM559669		
Hemidactylus longicephalus	Africa	DQ120416	HQ426544	DQ120246	HM559637	HM559670	HM559703	HQ426460
Hemidactylus mabouia	Africa	HM180323	HQ426545	EU268393	EU268361	EU268330	HM559704	HQ426462
Hemidactylus macropholis	Africa	DQ120343	HQ426547	DQ120208	JX041369	HQ426203	HQ426292	HQ426463
Hemidactylus maculatus	India	HM595699		HM595664		HM559674		
Hemidactylus modestus	Africa	DQ120386		DQ120215				
Hemidactylus oxyrhinus	Arabic Peninsula	DQ120344		DQ120173				
Hemidactylus palaichthus	America	DQ120434	HQ426548	EU268400	EU268368	EU268337		HQ426464
Hemidactylus persicus	Asia	HM595701		EU268409	EU268377	EU268346		
Hemidactylus platycephalus	Africa Madagascar	DQ120437	AY863045	DQ120270			JQ073246	
Hemidactylus platyurus	Asia IAA India		HQ426530	EU268384	EU268352	EU268321	HM559685	HQ426446
Hemidactylus prashadi	India	HM595702		HM595668		HM559676		
Hemidactylus pumilio	Arabic Peninsula	DQ120382		DQ120211				
Hemidactylus reticulatus	India	HM595705		EU268410		EU268347		
Hemidactylus robustus	Africa Arabic Peninsula	DQ120345	HQ426549	EU268408	EU268376	EU268345	EU054271	HQ426465

Hemidactylus sataraensis	India	HM595708		HM595672				
Hemidactylus triedrus	India	HM595709	HQ426550	HM595675		HM559683		HQ426466
Hemiphyllodactylus aurantiacus	India			FJ971012	JN393933	JN394011	JN393977	
Hemiphyllodactylus typus	Asia IAA India Pacific			FJ971011	GQ257744			
Hemiphyllodactylus yunnanensis	Asia		JQ945579	FJ971005	JN393949	JN394013	JN393979	JQ945472
Heteronotia binoei	Australia	NC_010292	JQ945580	NC_010292	NC_010292	EU054270	EU054285	JQ945473
Heteronotia planiceps	Australia		JQ945581		EU054300	EU054268	EU054284	JQ945474
Homopholis fasciata	Africa	DQ852723	DQ852735		EU054250	EU054202	EU054226	JQ945475
Homopholis mulleri	Africa				EU054241	EU054193	EU054217	
Homopholis walbergii	Africa				EU054248	EU054200	EU054224	
Lepidodactylus moestus	Pacific				JN019079	JN019111	JN019143	
Lepidodactylus novaeguineae	IAA		JQ945583		JX041378	JQ945380	JQ945312	JQ945476
Lepidodactylus orientalis	IAA				JN019080	JN019112	JN019144	
Luperosaurus cumingii	IAA		JQ945585		JQ437902	JQ945381	JQ945313	JQ945478
Lygodactylus arnoulti	Madagascar			GU593523			GU593585	GU593395
Lygodactylus bradfieldi	Africa	DQ852705	AY172935		EU423279	HQ426212	HQ426301	GU593360
Lygodactylus gravis	Africa			GU593500			GU593556	GU593366
Lygodactylus gutturalis	Africa			GU593522			GU593583	GU593393
Lygodactylus heterurus	Madagascar			GU593546			GU593611	GU593421
Lygodactylus klugei	America		HQ426555			HQ426209	HQ426298	HQ426471
Lygodactylus lawrencei	Africa			GU593496			GU593552	GU593362
Lygodactylus luteopicturatus	Africa		FJ830142	FJ830051			FJ830234	
Lygodactylus madagascariensis	Madagascar	EU596612		EU596695				
Lygodactylus miops	Madagascar		HQ426556			HQ426210	HQ426299	HQ426472
Lygodactylus mirabilis	Madagascar		HQ426557	GQ910848	JX041382	HQ426211	HQ426300	GU593413
Lygodactylus montanus	Madagascar			GU593536			GU593601	GU593411
Lygodactylus picturatus	Africa			FJ830051			JQ073235	FJ830325
Lygodactylus tolampyae	Madagascar		HQ426559	GU593526	JX041383	HQ426213	HQ426302	GU593399
Lygodactylus verticillatus	Madagascar			GU593529			GU593595	GU593404
Matoatoa brevipes	Madagascar	EU596614	JQ945587	EF490751	EF490777	EF490698	EF490724	JQ945480
Nactus acutus	IAA				EU054289	EU054257	EU054273	
Nactus cheverti	Australia				HM997154	HM997178	HM997166	
Nactus eboracensis	Australia				HM997156	HM997180	HM997168	
Nactus galgajuga	Australia				HM997158	HM997182	HM997170	
Nactus multicarinatus	Australia IAA				HM997160	HM997184	HM997172	
Nactus pelagicus	Pacific	DQ852706	AY172936		HM997163	HM997187	EU054275	JQ945484
Nactus vankampeni	IAA		JQ945591		EU054296	EU054264	EU054280	JQ945485
Narudasia festiva	Africa	DQ852707	EF534934		JX041387	EF534850	EF534808	EF534976
Pachydactylus affinis	Africa	DQ275389		AY123414			DQ275433	
Pachydactylus austeni	Africa	DQ275371	JQ945596	AF449126	JX041390	JQ945389	DQ275415	
Pachydactylus barnardi	Africa	DQ275378		AY123410			DQ275422	
Pachydactylus bicolor	Africa	DQ275394		AY123400			DQ275438	
Pachydactylus capensis	Africa	DQ275387		AF449133	HQ165962	HQ165977	DQ275431	

Pachydactylus caraculicus	Africa	DQ275393		AY123399			DQ275437	
Pachydactylus fasciatus	Africa	DQ275384		AF449128	HQ165949	HQ165964	DQ275428	
Pachydactylus formosus	Africa	DQ275377		AY123411			DQ275421	
Pachydactylus gaiasensis	Africa	DQ275399	JQ945597	AY123407	JX041391	JQ945390	DQ275443	JQ945490
Pachydactylus geitje	Africa	DQ275380		AF449132			DQ275424	
Pachydactylus haackei	Africa	DQ275402		AF449121			DQ275446	
Pachydactylus kladaroderma	Africa	DQ275401	JQ945598	AF449122	JX041392	JQ945391	DQ275445	JQ945491
Pachydactylus labialis	Africa	DQ275379		AF449130			DQ275423	
Pachydactylus maculatus	Africa	DQ275382		AF449127			DQ275426	
Pachydactylus mariquensis	Africa	DQ275370		DQ278601			DQ275414	
Pachydactylus mclachlani	Africa				HQ165951	HQ165966	HQ165981	
Pachydactylus monicae	Africa			DQ349180	HQ165953	HQ165968	HQ165983	
Pachydactylus namaquensis	Africa	DQ275400		AF449123			DQ275444	
Pachydactylus oculatus	Africa	DQ275381		AY123402			DQ275425	
Pachydactylus oreophilus	Africa	DQ275398		AY123406			DQ275442	
Pachydactylus oshaughnessyi	Africa	DQ275391		AY123413			DQ275435	
Pachydactylus parascutatus	Africa	DQ275397		AY123405			DQ275441	
Pachydactylus punctatus	Africa	DQ275411	EU293691	AF449135	JX041393	EU293713	EU293646	EU293736
Pachydactylus purcelli	Africa			DQ349161	HQ165955	HQ165970	HQ165985	
Pachydactylus rangei	Africa		JQ945599	AY123397	JX041394	JQ945392	JQ945324	JQ945492
Pachydactylus rugosus	Africa	DQ275376	JQ945600	AF449129	JX041395	JQ945393	DQ275420	JQ945493
Pachydactylus scherzi	Africa	DQ275392		AY123401			DQ275436	
Pachydactylus scutatus	Africa	DQ275395		AY123403			DQ275439	
Pachydactylus serval	Africa	DQ275385		DQ349168	HQ165957	HQ165972	DQ275429	
Pachydactylus tigrinus	Africa	DQ275390		AY123412			DQ275434	
Pachydactylus tsodiloensis	Africa	DQ275383		AY123408			DQ275427	
Pachydactylus vansoni	Africa	DQ275388		AY123415			DQ275432	
Pachydactylus vanzyli	Africa		JQ945601	AY123396	JX041396	JQ945394	JQ945326	JQ945494
Pachydactylus weberi	Africa	DQ275386	JQ945602	AF449131	HQ165961	HQ165976	DQ275430	JQ945495
Paragehyra gabriellae	Madagascar		JQ945603		JX041399	JQ945396	JQ945328	JQ945496
Paroedura androyensis	Madagascar	GU128974	HQ256721	EF490748	EF490774	EF490695	EF490721	
Paroedura bastardi	Madagascar	GU128980	HQ256725		EF536211	EF536187	EF536163	
Paroedura gracilis	Madagascar	GU128969	HQ256726		EF536209	EF536185	EF536161	
Paroedura homalorhina	Madagascar				EF536214	EF536190	EF536166	
Paroedura karstophila	Madagascar			EF490749	EF490775	EF490696	EF490722	
Paroedura lohatsara	Madagascar	GU128971	HQ256731		EF536203	EF536179	EF536155	
Paroedura masobe	Madagascar	GU128977	HQ426560		EF536193	EF536169	EF536145	HQ426478
Paroedura oviceps	Madagascar	EU596615	HQ256733		EF536208	EF536184	EF536160	
Paroedura picta	Madagascar	GU128965	EU293692		EF536198	EF536174	EF536150	EU293737
Paroedura sanctijohannis	Comoros				EF536205	EF536181	EF536157	
Paroedura stumpffi	Madagascar	GU128964	HQ256740		EF536202	EF536178	EF536154	
Paroedura tanjaka	Madagascar				EF536201	EF536177	EF536153	
Paroedura vazimba	Madagascar	GU128981	HQ256742		EF536195	EF536171	EF536147	

Perochirus ateles	Pacific		JQ945604		JN393946	JN394015	JN393984	JQ945497
Phelsuma abbotti	IAA	EU596618	AY221346	FJ829977	EU423290		FJ830148	FJ830239
Phelsuma andamanense	IAA	AY221277	FJ830063	FJ829982			FJ830155	FJ830246
Phelsuma antanosy	Madagascar		FJ830064	FJ829983			FJ830156	FJ830247
Phelsuma astriata	Seychelles	AY221273	AY221337	FJ829985	EU423286		FJ830158	FJ830249
Phelsuma barbouri	Madagascar		FJ830069	FJ829988	EU423289		FJ830161	FJ830252
Phelsuma berghofi	Madagascar		FJ830070	FJ829989			FJ830162	FJ830253
Phelsuma borbonica	MI	AY221286	AY221350	FJ829990	JX041400	HQ426216	FJ830163	HQ426479
Phelsuma breviceps	Madagascar		FJ830072	FJ829991			FJ830164	FJ830255
Phelsuma cepediana	MI	AY221294	FJ830073	FJ829992	EU423294		FJ830165	FJ830256
Phelsuma comorensis	Comoros	DQ911636					JQ073229	FJ830257
Phelsuma dubia	Africa	EF424452	FJ830080	FJ829996	EU423285		FJ830172	FJ830263
Phelsuma edwardnewtoni	MI	AY221292	AY221353	AY221398				
Phelsuma flavigularis	Madagascar	EF424466	FJ830082	FJ829997			FJ830174	FJ830265
Phelsuma gigas	MI	AY221293	AY221354	AY221399				
Phelsuma guentheri	MI	AY221310	FJ830084	FJ830000			FJ830176	FJ830267
Phelsuma guimbeaui	MI		AY221372	FJ830041	EU423291	HQ426217	HQ426306 FJ830221	HQ426480
Phelsuma guttata	Madagascar		FJ830085	FJ830001	EU423284		FJ830177	FJ830268
Phelsuma hielscheri	Madagascar		FJ830086	FJ830002			FJ830178	FJ830269
Phelsuma inexpectata	MI		FJ830087	FJ830003	JN393939	JN394016	FJ830179	FJ830270
Phelsuma kely	Madagascar		FJ830088	FJ830004			FJ830180	FJ830271
Phelsuma klemmeri	Madagascar		FJ830090	FJ830006			FJ830182	FJ830273
Phelsuma laticauda	Comoros Madagascar	DQ911673	AY221343	FJ830012	EU423281	JQ945398	FJ830189	FJ830280
Phelsuma lineata	Madagascar	AY221279	AY221342	FJ830021	EU423283		JN654861	FJ830285
Phelsuma madagascariensis	Madagascar	DQ852709	EF534937	EF424450	EU423288	AB081507	EF534811 FJ830175	EF534979
Phelsuma malamakibo	Madagascar		FJ830108	FJ830023			FJ830200	FJ830291
Phelsuma modesta	Madagascar	EF424465	FJ830110	EF424451	EU423287	HQ426218	FJ830202	HQ426481
Phelsuma mutabilis	Madagascar	AY221272	FJ830114	FJ830029			FJ830206	FJ830297
Phelsuma nigristriata	Comoros		FJ830117	FJ830032			FJ830209	FJ830300
Phelsuma ornata	MI	AY221323	FJ830118	FJ830033	EU423282		FJ830210	FJ830301
Phelsuma parkeri	Africa	DQ911693	FJ830119	DQ911751			FJ830211	FJ830302
Phelsuma pronki	Madagascar		FJ830121	FJ830034			FJ830213	FJ830304
Phelsuma pusilla	Madagascar		FJ830122	FJ830035			FJ830214	FJ830305
Phelsuma quadriocellata	Madagascar	AY221282	FJ830123	FJ830036			FJ830215	FJ830306
Phelsuma ravenala	Madagascar		FJ830078	FJ829994			FJ830170	FJ830261
Phelsuma robertmertensi	Comoros	DQ911643	FJ830128	DQ911701			FJ830220	FJ830311
Phelsuma seippi	Madagascar		FJ830130	FJ830042			FJ830222	FJ830313
Phelsuma serraticauda	Madagascar	AY221278	FJ830131	FJ830043	EU423296		FJ830223	FJ830314
Phelsuma standingi	Madagascar	AY221281	FJ830133	FJ830045			FJ830225 GU593609	FJ830316
Phelsuma sundbergi	Seychelles			FJ830048	EU423295		FJ830228	FJ830319
Phelsuma vanheygeni	Madagascar		FJ830137	FJ830049			FJ830229	FJ830320
Pseudogekko smaragdinus	IAA		JQ945608		JQ437897	JQ437940	JQ945332	JQ945501
Ptenopus carpi	Africa	DQ852711	AY172942		JX041422	JQ945402	JQ945333	JQ945502

Ptychozoon kuhli	IAA		JQ945610		JQ437918	JQ437960	JQ945334	JQ945503
Ptychozoon lionotum	IAA		JQ945611		JQ437914	JQ437956	JQ945335	JQ945504
Pygopus nigriceps	Australia	DQ852693	AY172943					
Rhoptropus afer	Africa	DQ275409	JQ945613	AY026923	JX041430	JQ945405	DQ275453	JQ945506
Rhoptropus boultoni	Africa	DQ852712	EF534936	AY026922	JX041431	EF534852	EF534810	EF534978
Rhoptropus bradfieldi	Africa	EU596606	JQ945614	AY026924	JX041432	JQ945406	JQ945337	JQ945507
Stenodactylus petrii	Africa Arabic Peninsula	DQ852722	DQ852738		HQ443558		JX440679	
Stenodactylus sthenodactylus	Africa Arabic Peninsula		JQ945617		HQ443546	JQ945408	JQ945339	JQ945510
Microgecko helenae	Asia		JQ945590		JX041386	JQ945385	JQ945317	JQ945483
Tropiocolotes tripolitanus	Africa		JQ945623		HQ443537	JQ945412	JQ945343	JQ945517
Urocotyledon inexpectata	Africa Seychelles		JQ945624	JN225804	JX041461	JN225672	JQ945344	JQ945518
Uroplatus alluaudi	Madagascar	EU596620		EF490766	EF490793	EF490713	EF490740	
Uroplatus ebenaui	Madagascar	EU596624	JN038097	EF490763	EF490788	EF490710	EF490736	
Uroplatus fimbriatus	Madagascar	EU596641			EF490792	EF490712	EF490739	
Uroplatus giganteus	Madagascar		JQ945625	EF490765	EF490791	EF490711	EF490738	JQ945519
Uroplatus guentheri	Madagascar	EU596689	JQ945626	EF490752	EF490778	EF490699	EF490725	JQ945520
Uroplatus henkeli	Madagascar	EU596650	HQ426591	EF490770	EF490797	EF490717	EF490744	HQ426510
Uroplatus lineatus	Madagascar	EU596656		EF490754	EF490780	EF490701	EF490727	
Uroplatus phantasticus	Madagascar	EU596664	HQ426592	EF490773	EF490800	EF490720	EF490747	HQ426511
Uroplatus pietschmanni	Madagascar	EU596687		EF490755	EF490781	EF490702	EF490728	
Uroplatus sikorae	Madagascar	EU596681		EF490758	EF490785	EF490706	EF490731	
Oedura marmorata	Australia				AY369015	EF534819	EF534779	EF534945
Sphaerodactylus elegans	America	KF017637	EF534912	KF017628	JN393942	EF534828	EF534787	EF534954
Sphaerodactylus roosevelti	America	DQ852713	AY172946		JN393943	EF534825	EF534785	EF534951
Woodworthia maculata	Australia		JQ945628		GU459852	GU459651	GU459449	JQ945522

The calibration points used in this run were taken from Heinicke, Daza, Greenbaum, Jackman, & Bauer (2014): one point for the Diplodactylidae (most recent common ancestor between *Oedura* and *Woodworthia*; exponential distribution; M = 17.0 and offset = 16.0 m.y.a.); one point for the Pygopodidae (most recent common ancestor between *Delma* and *Pygopus*; exponential distribution; M = 10.0 and offset = 20.0 m.y.a.); one point for the Sphaerodactylidae (most recent common ancestor between *Sphaerodactylus elegans* and *S. roosevelti*; exponential distribution; M = 3.0 and offset = 15.0 m.y.a.); and one point for the Gekkonidae (ingroup; lognormal distribution; M = 3.0 and S = 1.0; offset = 100.0 m.y.a.). In this last calibration point, we altered the offset from 97 to 100 m.y.a. based on the original description of *Cretaceogekko burmae* (Arnold & Poinar, 2008).

15. Typhlopoidea

Table S12: Genbank numbers for the sequences used in the Typhlopoidea analyses with the distribution attributed to each terminal

Species	Location	BDNF	BMP2	NT3	AMEL	RAG1
Acutotyphlops kunuaensis	IAA	GU902419	GU902499	GU902590	GU902339	GU902669
Acutotyphlops sp	IAA	GU902459	GU902552	GU902629	GU902379	GU902704

Acutotyphlops subocularis	IAA	GU902418	GU902498	GU902589	GU902338	GU902668
Afrotyphlops angolensis	Africa	GU902389	GU902469	GU902562	GU902312	GU902639
Afrotyphlops bibronii	Africa	GU902450	GU902528	GU902620	GU902370	GU902696
Afrotyphlops congestus	Africa	GU902448	GU902526	GU902618	GU902368	GU902694
Afrotyphlops elegans	Africa	GU902391	GU902471	GU902564	GU902314	GU902641
Afrotyphlops fornasinii	Africa	GU902447		GU902617	GU902367	GU902693
Afrotyphlops lineolatus	Africa	GU902451	GU902529	GU902621	GU902371	GU902697
Afrotyphlops mucruso	Africa	GU902387	GU902467	GU902560	GU902310	GU902637
Afrotyphlops obtusus	Africa		GU902548	GU902625	GU902375	GU902700
Afrotyphlops punctatus	Africa	GU902395	GU902475	GU902567	GU902318	GU902645
Afrotyphlops schlegelii	Africa	GU902449	GU902527	GU902619	GU902369	GU902695
Amerotyphlops brongersmianus	America	GU902390	GU902470	GU902563	GU902313	GU902640
Amerotyphlops reticulatus	America	GU902396	GU902476	GU902568	GU902319	GU902646
Anilios australis	Australia	GU902409	GU902489	GU902580	GU902331	GU902659
Anilios bicolor	Australia	GU902410	GU902490	GU902581	GU902332	GU902660
Anilios bituberculatus	Australia	GU902403	GU902483	GU902574	GU902325	GU902653
Anilios diversus	Australia	GU902411	GU902491	GU902582	GU902333	GU902661
Anilios endoterus	Australia	GU902399	GU902479	GU902570		GU902649
Anilios ganei	Australia	GU902412	GU902492	GU902583	GU902334	GU902662
Anilios grypus	Australia	GU902413	GU902493	GU902584		GU902663
Anilios guentheri	Australia	GU902404	GU902484	GU902575	GU902326	GU902654
Anilios hamatus	Australia	GU902401	GU902481	GU902572	GU902323	GU902651
Anilios howi	Australia	GU902414	GU902494	GU902585	GU902335	GU902664
Anilios kimberleyensis	Australia	GU902406	GU902486	GU902577	GU902328	GU902656
Anilios ligatus	Australia	GU902405	GU902485	GU902576	GU902327	GU902655
Anilios longissimus	Australia	GU902408	GU902488	GU902579	GU902330	GU902658
Anilios pilbarensis	Australia	GU902400	GU902480	GU902571	GU902322	GU902650
Anilios pinguis	Australia	GU902415	GU902495	GU902586	GU902336	GU902665
Anilios splendidus	Australia	GU902416	GU902496	GU902587	GU902337	GU902666
Anilios troglodytes	Australia	GU902417	GU902497	GU902588		GU902667
Anilios unguirostris	Australia	GU902407	GU902487	GU902578	GU902329	GU902657
Anilios waitii	Australia	GU902402	GU902482	GU902573	GU902324	GU902652
Antillotyphlops catapontus	America	GU902426	GU902506	GU902596	GU902346	GU902676
Antillotyphlops dominicanus	America	GU902428	GU902508	GU902598	GU902348	
Antillotyphlops geotomus	America	GU902429	GU902509	GU902599	GU902349	
Antillotyphlops granti	America	GU902430	GU902510	GU902600	GU902350	GU902678
Antillotyphlops hypomethes	America	GU902431	GU902511	GU902601	GU902351	GU902679
Antillotyphlops monastus	America	GU902434	GU902514	GU902604	GU902354	
Antillotyphlops naugus	America	GU902435	GU902515	GU902605	GU902355	GU902681
Antillotyphlops platycephalus	America	GU902437	GU902517	GU902607	GU902357	GU902683
Antillotyphlops richardi	America	GU902438	GU902518	GU902608	GU902358	GU902684
Cubatyphlops anchaurus	America	GU902423	GU902503	GU902593	GU902343	GU902673
Cubatyphlops anousius	America	GU902445	GU902524	GU902615	GU902365	GU902691

Cubatyphlops arator	America	GU902424	GU902504	GU902594	GU902344	GU902674
Cubatyphlops caymanensis	America	GU902427	GU902507	GU902597	GU902347	GU902677
Cubatyphlops contorhinus	America	GU902446	GU902525	GU902616	GU902366	GU902692
Cubatyphlops notorachius	America	GU902436	GU902516	GU902606	GU902356	GU902682
Gerrhopilus hedraeus	IAA	GU902392	GU902472	GU902565	GU902315	GU902642
Gerrhopilus mirus	India	GU902394	GU902474	GU902566	GU902317	GU902644
Indotyphlops albiceps	IAA	GU902382	GU902462	GU902555	GU902305	GU902632
Indotyphlops braminus	Africa Asia	GU902383	GU902463	GU902556	GU902306	GU902633
Indotyphlops pammeces	India	GU902458	GU902551	GU902628	GU902378	GU902703
Letheobia feae	Africa	GU902385	GU902465	GU902558	GU902308	GU902635
Letheobia newtoni	Africa	GU902388	GU902468	GU902561	GU902311	GU902638
Madatyphlops andasibensis	Madagascar	GU902453	GU902545	GU902622	GU902373	GU902698
Madatyphlops arenarius	Madagascar	GU902455	GU902547	GU902624	GU902374	GU902699
Malayotyphlops luzonensis	IAA	GU902393	GU902473		GU902316	GU902643
Ramphotyphlops acuticaudus	IAA	GU902381	GU902461	GU902554	GU902304	GU902631
Ramphotyphlops lineatus	IAA	GU902384	GU902464	GU902557	GU902307	GU902634
Ramphotyphlops sp	IAA	GU902420	GU902500		GU902340	GU902670
Rhinotyphlops lalandei	Africa	GU902386	GU902466	GU902559	GU902309	GU902636
Rhinotyphlops unitaeniatus	Africa	GU902452	GU902530		GU902372	
Sundatyphlops polygrammicus	IAA	GU902421	GU902501	GU902591	GU902341	GU902671
Typhlophis squamosus	America	GU902398	GU902478		GU902321	GU902648
Typhlops agoralionis	America	GU902422	GU902502	GU902592	GU902342	GU902672
Typhlops capitulatus	America	GU902425	GU902505	GU902595	GU902345	GU902675
Typhlops eperopeus	America	GU902444	GU902523	GU902614	GU902364	GU902690
Typhlops jamaicensis	America	GU902432	GU902512	GU902602	GU902352	
Typhlops lumbricalis	America	GU902433	GU902513	GU902603	GU902353	GU902680
Typhlops rostellatus	America	GU902439	GU902546	GU902609	GU902359	GU902685
Typhlops schwartzi	America	GU902440	GU902519	GU902610	GU902360	GU902686
Typhlops sp	America	GU902454	GU902553	GU902623	GU902380	GU902705
Typhlops sulcatus	America	GU902441	GU902520	GU902611	GU902361	GU902687
Typhlops sylleptor	America	GU902442	GU902521	GU902612	GU902362	GU902688
Typhlops syntherus	America	GU902443	GU902522	GU902613	GU902363	GU902689
Xenotyphlops grandidieri	Madagascar	GU902457	GU902550	GU902627	GU902377	GU902702
Xenotyphlops sp	Madagascar	GU902456	GU902549	GU902626	GU902376	GU902701
Xerotyphlops vermicularis	Eurasia	GU902397	GU902477	GU902569	GU902320	GU902647

16. Natricinae

Table S13: Genbank numbers for the sequences used in the Natricinae analyses with the distribution attributed to each terminal.

Species	Locality	12S	CMOS	COI	CytB	ND1	ND2	ND4
Adelophis foxi	America				AF420069	AF420070	AF420071	AF420072
Afronatrix anoscopus	Africa		AF471123		AF420073	AF420074	AF420075	AF420076

Amphiesma craspedogaster	Asia		JQ687437		JQ687429		JQ687459	JQ687412
Amphiesma sauteri	Asia	AF402622			AF402905		AF384824	
Amphiesma stolatum	IAA		JQ687450		JQ687432		JQ687464	JQ687425
Atretium yunnanensis	Asia		JQ687448		GQ281787		JQ687463	JQ687423
Clonophis kirtlandii	America	AF402625			AF402908		AF384827	
Coluber constrictor	America	AY122667	AY486937	AY122649	AY486913	AY486962	AY487001	AY487040
Enhydris plumbea	IAA	DQ343650	EF395934	DQ343650	DQ343650	DQ343650	DQ343650	DQ343650
Imantodes cenchoa	America	EU728586	GQ457865	EU728586	EU728586	EU728586	EU728586	EU728586
Lycognathophis seychellensis	MI		FJ387220					
Macropisthodon rudis	Asia		JQ687442		GQ281780		JQ687458	JQ687417
Natriciteres olivacea	Africa	AF544772	AF471146		AF471058			
Natrix maura	Africa	AF402623			AY866530	AY873742	AY870616	EU437572
Natrix natrix	Eurasia	AY122682	AF471121	AY122664	AF471059	AY873760	AY870640	AY873736
Natrix tessellata	Arabic Peninsula				EU119168	AY873772	AY870641	AY873735
Nerodia cyclopion	America	AF402626		GQ279084	AF402909		AF384828	
Nerodia erythrogaster	America	AF402629		GQ279023	GQ285504	HQ121595	GQ285402	AF420084
Nerodia fasciata	America	AF402627		GQ279080	AY866529	AY873738	AY870612	AY873705
Nerodia floridana	America	AF402628			AF402911		AF384830	
Nerodia harteri	America	AF402652			AF402935		AF384854	
Nerodia rhombifer	America			GQ279077	GQ285446	HQ121995	GQ285289	
Nerodia sipedon	America	AF402630		GQ278935	AF402913	HQ121602	DQ915161	
Nerodia taxispilota	America	AF402631		GQ279082	AF402914	HQ121606	AF384833	U49322
Opisthotropis cheni	Asia		JQ687441		GQ281779		JQ687457	JQ687416
Opisthotropis guangxiensis	Asia		JQ687447		GQ281776		JQ687462	JQ687422
Opisthotropis lateralis	Asia		JQ687445		GQ281782		JQ687461	JQ687420
Opisthotropis latouchii	Asia		JQ687446		GQ281783			JQ687421
Regina alleni	America	AF402633			AF402916		AF384835	
Regina grahami	America	AF402635			AF402918	HQ121593	AF384837	
Regina rigida	America	AF402636	AF471120		AF471052		AF384838	
Regina septemvittata	America	AF402634			AF402917		AF384836	
Rhabdophis nuchalis	Asia	AF402624	JQ687438		AF402907		AF384826	JQ687413
Rhabdophis subminiatus	Asia	AF544776	AF544713		GQ281777			U49325
Rhabdophis tigrinus	Asia	AF236679	AF471119		AF471051		JQ687460	JQ687419
Seminatrix pygaea	America	AF402637			AF402920		AF384839	
Sinonatrix aequifasciata	Asia		JQ687440		JQ687430		JQ687456	JQ687415
Sinonatrix annularis	Asia	AF236677	AF544712		JQ687431			
Sinonatrix percarinata 1	Asia		JQ687439		GQ281784		JQ687455	JQ687414
Sinonatrix percarinata 2	IAA		JQ687451		JQ687433		JQ687465	JQ687426
Storeria dekayi	America	AF402639	AF471154	EF417389	AF471050	EF417436	EF417460	EF417365
Storeria occipitomaculata	America	AF402638			AF402921		AF384840	U49323
Thamnophis atratus	America				AF420085	AF420086	AF420087	AF420088
Thamnophis brachystoma	America				AF420089	AF420090	HM630351	AF420092
Thamnophis butleri	America	AF402640			AF402923	AF420093	HM630350	AF420095

Thamnophis chrysocephalus	America			EF460849	AF420108	AF420096	AF420097	AF420098
Thamnophis couchii	America	AF402653			AF402936	AF420104	AF384855	AF420106
Thamnophis cyrtopsis	America	AF402641		EF417388	EF417412	EF417435	EF417459	EF417364
Thamnophis elegans	America	AF402642			AF402925	AF420114	AF384844	AF420116
Thamnophis eques	America				AF420117	AF420118	AF420119	AF420120
Thamnophis exsul	America				AF420125	AF420126	AF420127	AF420128
Thamnophis fulvus	America				AF420129	AF420130	AF420131	AF420132
Thamnophis gigas	America				AF420133	AF420210	AF420209	AF414099
Thamnophis godmani	America		AF471165		AF420135	AF420136	AF420137	AF420138
Thamnophis hammondii	America				AF420139	AF420140	AF420141	AF420142
Thamnophis marcianus	America	AF402643			AF402926	AF420144	AF384845	AF420146
Thamnophis melanogaster	America			EF417386	EF417410	EF417433	EF417457	EF417362
Thamnophis mendax	America				AF420151		AF420152	
Thamnophis ordinoides	America	AF402644			AF402927	AF420158	AF384846	AF420160
Thamnophis proximus	America	AF402645			AF402928	AF420162	AF384847	AF420164
Thamnophis radix	America	AF402651			AF402934	AF420170	HM630344	AF420172
Thamnophis rufipunctatus	America				AF420173	AF420174	AF420175	AF420176
Thamnophis sauritus	America				AF420177	AF420178	AF420179	AF420180
Thamnophis scalaris	America				AF420181	AF420182	AF420183	AF420184
Thamnophis scaliger	America				AF420189	AF420190	AF420191	AF420192
Thamnophis sirtalis	America	AF402647	DQ902094		AF402929	AF420194	DQ995396	AY136272
Thamnophis sumichrasti	America				AF420197	AF420198	AF420199	AF420200
Thamnophis valida	America			EF417385	EF417408	EF417432	EF417437	EF417360
Trachischium monticola	India		JQ687453		JQ687435			JQ687428
Tropidoclonion lineatum	America				AF420205	AF420206	AF420207	AF420208
Virginia striatula	America	AF402650			AF402933		AF384852	
Xenochrophis flavipunctatus	IAA	AF544780	AF544714				FJ416748	
Xenochrophis piscator	IAA India	GQ225679	GQ225669	GQ225666	GQ225659			
Xenochrophis punctulatus	IAA		AF471106		AF471079	AY486996	AY487035	AY487074
Xenochrophis schnurrenbergeri	India	GQ225678	GQ225668	GQ225663	GQ225660			
Xenochrophis vittatus	IAA	EF395871	EF395920		EF395895			

In these analyses, we used the same calibration points as the original article, but we have made some alterations in the distribution parameters: for instance, in the Natricinae calibration, we set a lognormal distribution with offset of 30 m.y.a., mean of 0.4, and a standard deviation of 0.8. This yielded a 95% confidence interval from 30 to 37 m.y.a., which included the age of the fossil (30-32 m.y.a.) and set a soft constraint in the lower bound of the distribution. The same principle was applied fro the other two points: the *Natrix* calibration point (lognormal distribution; M = 0.4 and S = 0.9; offset = 22.0 m.y.a.) and the *Thamnophis* (lognormal distribution; M = 0.8 and S = 0.72; offset = 16.0 m.y.a.). We combined the logs for three independent analyses to obtain the final result.

17. Testudinidae

Table S14: Genbank numbers for the sequences used in the Testudinidae analyses with the distribution attributed to each terminal.

Species	Location	12S	16S	CytB	CMOS	RAG2
Chersina angulata	Africa	DQ497248	DQ497269	DQ497292	DQ497325	DQ497361
Aldabrachelys arnoldii	Seychelles	AY081779	AY081780	DQ497293	DQ497326	DQ497362
Aldabrachelys dussumieri	Seychelles	DQ497249	DQ497270	DQ497294	DQ497327	DQ497363
Aldabrachelys hololissa	Seychelles	AY081783	AY081784	DQ497295	DQ497328	DQ497364
Chelonoidis carbonarius	America	AB090019	AF192926	DQ497296	DQ497329	DQ497365
Chelonoidis chilensis	America	AF175336	AF192924	DQ497297	DQ497330	DQ497366
Chelonoidis denticulatus	America	DQ497250	AF192927	DQ497298	DQ497331	DQ497367
Geochelone elegans	India	AY081785	AY081786	DQ497299	DQ497332	DQ497368
Chelonoidis nigra	America	AF020885	AF020892	DQ497300	DQ497333	DQ497369
Stigmochelys pardalis pardalis	Africa	DQ497251	DQ497271	DQ497301	DQ497334	DQ497370
Stigmochelys pardalis babcocki	Africa	DQ497252	DQ497272	DQ497302	DQ497335	DQ497371
Geochelone platynota	IAA	DQ497253	DQ497273	DQ497303	DQ497336	DQ497372
Astrochelys radiata	Madagascar	AF020883	AF020890	DQ497304	DQ497337	DQ497373
Centrochelys sulcata	Africa	AY081787	AY081788	DQ497305	DQ497338	DQ497374
Astrochelys yniphora	Madagascar	AF020882	AF020889	DQ497306	DQ497339	DQ497375
Gopherus agassizii	America	AY434630		AY434562		
Gopherus polyphemus	America	AF020879	AF020886	DQ497307	DQ497340	DQ497376
Homopus boulengeri	Africa	DQ497254	DQ497274	DQ497308	DQ497341	DQ497377
Homopus signatus	Africa	DQ497255	DQ497275	DQ497309	DQ497342	DQ497378
Indotestudo elongata	Asia IAA	DQ497256	DQ497276	DQ497310	DQ497343	DQ497379
Indotestudo forstenii	IAA	DQ080044	DQ080044	DQ080044		
Indotestudo travancorica	India	DQ497257	DQ497277	DQ497311	DQ497344	DQ497380
Kinixys belliana	Africa	DQ497258	DQ497278	DQ497312	DQ497345	DQ497381
Kinixys homeana	Africa	DQ497259	DQ497279	DQ497313	DQ497346	DQ497382
Malacochersus tornieri	Africa	DQ497260	DQ497280	DQ497314	DQ497347	DQ497383
Manouria emys emys	IAA	DQ497261	DQ497281	DQ497315	DQ497348	DQ497384
Manouria emys phayrei	Asia	DQ497262	DQ497282	DQ497316	DQ497349	DQ497385
Manouria impressa	Asia IAA	DQ497263	DQ497283	DQ497317	DQ497350	DQ497386
Psammobates tentorius	Africa	DQ497264	DQ497284	DQ497318	DQ497351	DQ497387
Pyxis arachnoides	Madagascar	AF020880	AF020887	DQ497319	DQ497352	DQ497388
Pyxis planicauda	Madagascar	AF020881	AF020888	DQ497320	DQ497353	DQ497389
Testudo graeca	Africa Arabic Peninsula	AF175330	DQ497285	DQ497321	DQ497354	DQ497390
Agrionemys horsfieldii	Asia	AB090020	DQ497286	DQ497322	DQ497355	DQ497391
Testudo kleinmanni	Africa	AF175332	DQ497287	DQ497323	DQ497356	DQ497392
Cylindraspis indica	MI			AF371247		
Cylindraspis peltastes	MI			AF371256		
Cylindraspis vosmaeri	MI			AF371260		
Glyptemys insculpta	America	DQ497265	DQ497288	AF258876	DQ497357	DQ497393

Deirochelys reticularia	America	DQ497266	DQ497289	AF258877	DQ497358	DQ497394
Rhinoclemmys melanosterna	America	DQ497267	DQ497290	AY434590	DQ497359	DQ497395
Rhinoclemmys nasuta	America	DQ497268	DQ497291	DQ497324	DQ497360	DQ497396
Platysternon megacephalum	Asia	DQ256377	DQ256377	DQ256377		KC683679

The calibration points for this analysis were taken from (Joyce, Parham, Lyson, Warnock, & Donoghue, 2013): a calibration point was set for the outgroup based on the Emydidae-*Platysternon* (lognormal distribution; M = 3.6 and S = 0.289; offset = 32.0 m.y.a.) and another calibration point was set to the root based on the age of the Testudinoidea (lognormal distribution; M = 3.12 and S = 0.4; offset = 50.3 m.y.a.). These parameters were set in a way that the offset was the minimum age constraints used by Joyce et al. (2013) and the median of the distribution was the lower bound of the 95% confidence interval obtained by their analyses. In this way, we allowed soft constraints for both upper and lower bounds of the calibration distribution to avoid age overestimation. We made three independent runs and combined their results through LogCombiner.

18. Falconidae

Table S15: Genbank numbers for the sequences used in the Falconidae analyses with the distribution attributed to each terminal

Species	Location	FGB	MB	ND1	PEPCK	PER2	RAG1	TGFB2	VIM
Accipiter striatus	America	KM875747	KM875875	KM875999				KM876435	
Caracara cheriway	America	KM875858	KM875961	KM876089	KM876202		KM876413	KM876543	KM876660
Caracara plancus	America	JN650312	JN650325	JN650427	JF909810	JN650357	JN650344	JN650374	JN650392
Cathartes aura	America	KX534487	KM875884	KM876007	KM876121	KM876224	KM876316	KM876444	KM876576
Coragyps atratus	America	KM875756	KM875883	KM876006	KM876120	KM876223	KM876315	KM876443	KM876574
Daptrius ater	America	JN650302	JN650317	JN650434	JN650402	JN650350	JN650333	JN650366	JN650385
Falco alopex	Africa	KM875801	KM875978	KM876043				KM876492	KM876610
Falco amurensis	Africa Asia	KM875836	KM875942	KM876070	KM876180	KM876291	KM876372	KM876521	KM876637
Falco araeus	Seychelles	KM875807	KM875915	KM876044	KM876159	KM876262	KM876351	KM876493	KM876611
Falco ardosiaceus	Africa	KM875781	KM875969	KM876023				KM876471	
Falco berigora	Australia	KM875735	KM875869	KM875987	KM876098	KM876302	KM876386	KM876423	KM876553
Falco biarmicus	Africa Asia	KM875788	KM875954	KM876082	KM876194	KM876303	KM876378	KM876477	KM876653
Falco cenchroides	Australia	KM875734	KM875868	KM875986	KM876099	KM876212	KM876387	KM876424	KM876555
Falco cherrug	Asia	KM875798	KM875911	KM876036	KM876154	KM876275	KM876403	KM876506	KM876606
Falco chicquera	Africa India	KM875856	KM875968	KM876087				KM876541	
Falco columbarius	America Asia Europe	KM875753	KM875876	KM876000	KM876110	KM876220	KM876321	KM876436	KM876567
Falco concolor	Africa	KM875759	KM875887	KM876011	KM876125	KM876228	KM876395	KM876448	KM876581
Falco cuvierii	Africa	KM875731	KM875972	KM875983				KM876420	
Falco deiroleucus	America	KM875815	KM875922	KM876051	KM876165	KM876271	KM876357	KM876500	KM876619
Falco dickinsoni	Africa	KM875793	KM875967	KM876031				KM876480	
Falco eleonorae	Africa Madagascar	KM875774	KM875896	KM876085	KM876138	KM876243	KM876338	KM876464	KM876657
Falco fasciinucha	Africa	KM875822	KM875930	KM876057	KM876171	KM876277	KM876408	KM876509	KM876626
Falco femoralis	America	KM875764	KM875952	KM876080	KM876192	KM876301	KM876376	KM876532	KM876651

Falco hypoleucos	Australia	KM875732	KM875866	KM875984				KM876421	
Falco jugger	India			KM875998					
Falco longipennis	Australia	KM875741	KM875873	KM875994	KM876106	KM876213	KM876390	KM876431	KM876562
Falco mexicanus	America	KM875752	KM875879	KM876002	KM876114	KM876219	KM876324	KM876495	KM876570
Falco moluccensis	IAA	KM875831	KM875937	KM876066				KM876516	
Falco naumanni	Africa Asia	KM875839	KM875951	KM876079	KM876191	KM876299	KM876375	KM876531	KM876650
Falco newtoni	Madagascar Seychelles	KM875777	KM875900	KM876024	KM876143	KM876247	KM876340	KM876467	KM876597
Falco novaeseelandiae	New Zealand	KM875766	KM875892	KM876014	KM876130	KM876237	KM876333	KM876455	KM876584
Falco pelegrinoides	America Asia Europe	KM875768	KM875981		KM876129	KM876235	KM876330	KM876452	KM876582
Falco peregrinus	Cosmopolite	KM875751	KM875979	KM876019	KM876137	KM876242	KM876416	KM876445	KM876590
Falco punctatus	Mascarene		KM875925	KM876053	KM876167	KM876272	KM876358	KM876503	KM876621
Falco rufigularis	America	KM875804	KM875908	KM876033	KM876151	KM876256	KM876347	KM876490	KM876604
Falco rupicoloides	Africa	KM875851	KM875924	KM876052	KM876195	KM876304	KM876379	KM876502	KM876654
Falco rupicolus	Africa	KM875785	KM875902	KM876026	KM876146	KM876250	KM876344	KM876527	KM876645
Falco rusticolus	America Asia Europe	KM875827	KM875932	KM876058	KM876173	KM876279	KM876362	KM876510	KM876628
Falco severus	India IAA	KM875799	KM875971	KM876037					
Falco sparverius	America	KM875749	KM875877	KM876004	KM876112	KM876215	KM876382	KM876540	KM876568
Falco subbuteo	Africa Asia	KM875845	KM875950	KM876078	KM876190	KM876297	KM876411	KM876529	KM876665
Falco subniger	Australia	KM875733	KM875867	KM875985	KM876096	KM876240	KM876336	KM876461	KM876551
Falco tinnunculus	Africa Asia IAA India	KM875865	KM875949	KM876077	KM876188	KM876296	KM876374	KM876528	KM876646
Falco vespertinus	Africa Asia Europe	KM875805	KM875914	KM876042	KM876158	KM876261	KM876350	KM876491	KM876609
Falco zoniventris	Madagascar	KM875802		KM876038				KM876546	
Herpetotheres cachinnans	America	KM875770		KM876040	KM876157	KM876260	KM876398	KM876459	KM876608
Ibycter americanus	America	KM875857	KM875960	KM876088	KM876201		KM876417	KM876542	KM876659
Micrastur buckleyi	America			JF909874					
Micrastur gilvicollis	America	JF899902	JF909632	JF909866	JF909800	JF909714	JF909670	JF909843	JF909759
Micrastur mintoni	America	JF899891	JF909616	JF909860	JF909783	JF909693	JF909654	JF909824	JF909740
Micrastur mirandollei	America	JF899906	JF909631	JF909873	JF909798	JF909712	JF909669	JF909842	JF909758
Micrastur plumbeus	America	KM875860			KM876204		KM876415	KM876545	KM876662
Micrastur ruficollis	America	KM875769		KM876015	KM876133		KM876397	KM876458	KM876661
Micrastur semitorquatus	America	JF899914	JF909623	JF909870	JF909808	JF909721	JF909676	JF909850	JF909765
Microhierax caerulescens	Asia IAA India	KM875833	KM875939	KM876068	KM876177	KM876283	KM876365	KM876518	KM876632
Microhierax erythrogenys	IAA	KM875784	KM875906	KM876025	KM876144	KM876248	KM876343	KM876473	KM876598
Microhierax fringillarius	IAA	KM875792	KM875975	KM876064				KM876515	
Microhierax latifrons	IAA	KM875791	KM875977	KM876030				KM876479	
Microhierax melanoleucos	Asia India	KM875780	KM875976	KM876022				KM876470	
Milvago chimachima	America	JN650309	JN650326	JN650430	JN650411	JN650358	JN650342	JN650375	JN650393
Milvago chimango	America								KM876636
Phalcoboenus albogularis	America	JN650316	KM875935	JN650423					JN650401
Phalcoboenus australis	America		JN650319	JN650435	JN650403	JN650351	JN650334	JN650367	JN650386
Phalcoboenus carunculatus	America	KM875758	KM875886	KM876010	KM876124		KM876394	KM876447	KM876577
Phalcoboenus megalopterus	America	JF899903	JF909628	JN650422	JN650417	JN650362	JN650347	JN650380	JN650398
Polihierax insignis	IAA	KM875829	KM875936	KM876063				KM876514	

Polihierax semitorquatus	Africa	KM875834	KM875940	KM876069	KM876178	KM876284	KM876366	KM876519	KM876633
Spiziapteryx circumcincta	America	JN650306	JN650323	JN650432	JN650408	JN650355	JN650339	JN650371	JN650390

19. *Aphananthe*

Table S16: Genbank numbers for the sequences used in the *Aphananthe* analyses with the distribution attributed to each terminal.

Species	Locality	psbA-trnH	trnL-trnF	ETS	ITS
Aphananthe cuspidata	Asia	KR086759	KR086777	KR086725	KR086741
A. cuspidata	IAA	KR086760	KR086778	KR086726	KR086742
A. monoica	America	KR086763	KR086779	KR086729	KR086745
A. monoica	America	KR086764	KR086780	KR086730	KR086746
A. philippinensis	Australia IAA	KR086765	KR086781	KR086731	KR086747
A. philippinensis	Australia IAA	KR086766	JN040357	KR086732	KR086748
A. philippinensis	Australia IAA	KR086767	KR086782	KR086733	KR086749
A. philippinensis	Australia IAA	KR086768	KR086783 -		KR086750
A. sakalava	Madagascar	KR086770	KR086784 -		KR086752
A. aspera	Asia	KR086753	KR086771	KR086719	KR086735
A. aspera	Asia	KR086754	KR086772	KR086720	KR086736
A. aspera	Asia	KR086755	KR086773	KR086721	KR086737
A. aspera	Asia	KR086756	KR086774	KR086722	KR086738
Celtis bungeana	Asia	KR086757	KR086775	KR086723	KR086739
C. julianae	Asia	KR086758	KR086776	KR086724	KR086740
Gironniera subequalis	Asia	KR086761	JN040375	KR086727	KR086743
Lozanella permollis	America	KR086762	JN040379	KR086728	KR086744
Pteroceltis tatarinowii	Asia	KR086769	JN040385	KR086734	KR086751

20. Exacum

Table S17: Genbank numbers for the sequences used in the *Exacum* analyses with the distribution attributed to each terminal.

Species	Locality	trnL	ITS
Exacum affine	Arabic Peninsula	AJ490202	AJ489877
Exacum affine	Arabic Peninsula	AJ490203	AJ489878
Exacum affine	Arabic Peninsula	AJ490204	AJ489879
Exacum atropurpureum	India	AJ490205	AJ489880
Exacum bulbilliferum	Madagascar	AJ490206	AJ489881
Exacum caeruleum	Arabic Peninsula	AJ490207	AJ489882
Exacum dolichantherum	Madagascar	AJ490208	AJ489883
Exacum exiguum	Madagascar	AJ490209	AJ489884
Exacum fruticosum	Madagascar	AJ490210	AJ489885
Exacum gracilipes	Arabic Peninsula	AJ490211	AJ489886
Exacum hamiltonii	India	AJ490212	AJ489887

Exacum humbertii	Madagascar	AJ490213	AJ489888
Exacum intermedium	Madagascar	AJ490214	AJ489889
Exacum linearifolium	Madagascar	AJ490215	AJ489890
Exacum macranthum	India	AJ490216	AJ489891
Exacum macranthum	India	AJ490217	AJ489892
Exacum marojejyense	Madagascar	AJ490218	AJ489893
Exacum microcarpum	Madagascar	AJ490219	AJ489894
Exacum millotii	Madagascar	AJ490220	AJ489895
Exacum nummularifolium	Madagascar	AJ490221	AJ489896
Exacum oldenlandioides	Africa	AJ490222	AJ489897
Exacum pallidum	India	AJ490223	AJ489898
Exacum pedunculatum	India	AJ490224	AJ489899
Exacum quinquenervium	Madagascar	AJ490225	AJ489900
Exacum sessile	India	AJ490226	AJ489901
Exacum stenophyllum	Madagascar	AJ490227	AJ489902
Exacum subacaule	Madagascar	AJ490228	AJ489903
Exacum subteres	Madagascar	AJ490229	AJ489904
Exacum subverticillatum	Madagascar	AJ490230	AJ489905
Exacum sutaepense	IAA	AJ490231	AJ489906
Exacum tetragonum	India	AJ490232	AJ489907
Exacum tetragonum	India	AJ490233	AJ489908
Exacum trinervium	India	AJ490234	AJ489909
Exacum trinervium var. ritigalense	India	AJ490235	AJ489910
Exacum walkeri	India	AJ490236	AJ489911
Exacum walkeri	India	AJ490237	AJ489912
Exacum wightianum	India	AJ490238	AJ489913
Gentianothamnus madagascariensis	Madagascar	AJ490240	AJ489914
Tachiadenus carinatus	Madagascar	AJ490249	AJ489923
Tachiadenus longiflorus	Madagascar	AJ490250	AJ489924
Ornichia madagascariensis	Madagascar	AJ490242	AJ489917
Ornichia trinervis	Madagascar	AJ490243	AJ489918

21. *Ficus*

Table S18: Genbank numbers for the sequences used in the *Ficus* analyses with the distribution attributed to each terminal.

Species	Locality	ITS	ETS	G3pdh	ncpGS
Ficus alongensis 1	Asia	KJ845962	KJ845902	KJ846015	
Ficus alongensis 2	Asia	KJ845963	KJ845903		
Ficus arnottiana 1	India		KJ845879		
Ficus arnottiana 2	India		KJ845880		
Ficus caulocarpa 1	IAA	KJ845953			
Ficus caulocarpa 2	IAA	KJ845954	KJ845894	KJ846009	

Ficus caulocarpa 3	IAA	KJ845955	KJ845895	KJ846010	
Ficus concinna 1	IAA	KJ845989	KJ845928	KJ846035	
Ficus concinna 2	IAA	KJ845990	KJ845929	KJ846036	
Ficus concinna 3	IAA	KJ845991	KJ845930	KJ846037	KJ846071
Ficus concinna 4	IAA	KJ845992	KJ845931	KJ846038	KJ846072
Ficus cordata 1	Africa	KJ845973	KJ845912	KJ846020	
Ficus cordata 2	Africa	KJ845974	KJ845913	KJ846021	
Ficus cordata 3	Africa	KJ845975	KJ845914	KJ846022	KJ846063
Ficus densifolia 1	Mascarene	KJ845983	KJ845922	KJ846030	KJ846068
Ficus densifolia 2	Mascarene	KJ845984	KJ845923	KJ846031	KJ846069
Ficus densifolia 3	Mascarene	KJ845985	KJ845924	KJ846032	
Ficus densifolia 4	Mascarene	KJ845986	KJ845925	KJ846033	KJ846070
Ficus geniculata geniculata 1	IAA	KJ845940	KJ845882	KJ845999	KJ846044
Ficus geniculata geniculata 2	IAA	KJ845941	KJ845883	KJ846000	KJ846045
Ficus geniculata geniculata 3	IAA	KJ845942	KJ845884		KJ846046
Ficus geniculata insignis	Australia	KJ845943	KJ845885	KJ846001	KJ846047
Ficus glaberrima siamensis 1	IAA	KJ845996	KJ845935	KJ846041	KJ846076
Ficus glaberrima siamensis 2	IAA	KJ845997	KJ845936	KJ846042	KJ846077
Ficus glaberrima siamensis 3	IAA	KJ845998	KJ845937	KJ846043	
Ficus henneana 1	Australia	KJ845967		KJ846016	KJ846058
Ficus henneana 2	Australia	KJ845968	KJ845907		KJ846059
Ficus hookeriana hookeriana	India	KJ845988	KJ845927		
Ficus ingens 1	Africa	KJ845964	KJ845904		KJ846056
Ficus ingens 2	Africa	KJ845965	KJ845905		
Ficus ingens 3	Africa	KJ845966	KJ845906		KJ846057
Ficus lecardii1	Africa	KJ845971	KJ845910	KJ846018	KJ846061
Ficus lecardii2	Africa	KJ845972	KJ845911	KJ846019	KJ846062
Ficus madagascariensis	Madagascar	KJ845956	KJ845896		KJ846053
Ficus middletonii	IAA	KJ845952	KJ845893	KJ846008	KJ846052
Ficus orthoneura 1	IAA	KJ845987	KJ845926	KJ846034	
Ficus prasinicarpa 1	IAA	KJ845947			
Ficus prasinicarpa 2	Australia	KJ845948	KJ845889		
Ficus prolixa 1	Pacific	KJ845949	KJ845890	KJ846005	KJ846051
Ficus prolixa 2	Pacific	KJ845950	KJ845891	KJ846006	
Ficus pseudoconcinna	IAA	KJ845946	KJ845888	KJ846004	KJ846050
Ficus religiosa 1	Asia / India	KJ845980	KJ845919	KJ846027	KJ846066
Ficus religiosa 2	IAA	KJ845981	KJ845920	KJ846028	
Ficus religiosa 3	IAA	KJ845982	KJ845921	KJ846029	KJ846067
Ficus cf rumphii	IAA	KJ845995	KJ845934		KJ846075
Ficus rumphii 1	IAA	KJ845993	KJ845932	KJ846039	KJ846073
Ficus rumphii 2	IAA	KJ845994	KJ845933	KJ846040	KJ846074
Ficus salicifolia 1	Africa	KJ845976	KJ845915	KJ846023	KJ846064
Ficus salicifolia 2	Arabic Peninsula	KJ845977	KJ845916	KJ846024	

Ficus subpisocarpa pubipoda 1	IAA	KJ845969	KJ845908		
Ficus subpisocarpa pubipoda 2	IAA	KJ845970	KJ845909	KJ846017	KJ846060
Ficus superba 1	IAA	KJ845944	KJ845886	KJ846002	KJ846048
Ficus superba 2	IAA	KJ845945	KJ845887	KJ846003	KJ846049
Ficus tsjakela	India	KJ845951	KJ845892	KJ846007	
Ficus verruculosa 1	Africa	KJ845978	KJ845917	KJ846025	
Ficus verruculosa 2	Africa	KJ845979	KJ845917	KJ846026	KJ846065
Ficus virens glabella 1	IAA	KJ845960	KJ845900	KJ846013	KJ846055
Ficus virens glabella 2	IAA	KJ845961	KJ845901	KJ846014	
Ficus virens virens 1	Australia	KJ845957	KJ845897	KJ846011	KJ846054
Ficus virens virens 2	IAA	KJ845958	KJ845898	KJ846012	
Ficus virens virens 3	Australia	KJ845959	KJ845899		
Ficus virens virens 4	India	KJ845938	KJ845881		
Ficus virens virens 5	India	KJ845939			

22. Loranthaceae

Table S19: Genbank numbers for the sequences used in the Loranthaceae analyses with the distribution attributed to each terminal

Species	Location	LSU	SSU	matK	rbcL	trnL-F
Actinanthella menyharthii	Africa	EU544352	EU544313	EU544408		
Aetanthus nodosus	America		EU544314	EU544409		
Agelanthus sansibarensis	Africa	EU544353	U59946	EU544410	EU544464	DQ340573
Agelanthus zizyphifolius	Africa	MG999385	MG999460	MG999409	MG999430	MG999482
Alepis flavida	New Zealand	EF464474	L24139	EF464508	KT626664	EF464481
Amyema queenslandica	Australia	EU544355	EU544315	EU544412		
Amyema glabra	Australia	EU544354	AF039073	EU544411	EU544465	EU544476
Amylotheca duthieana	IAA	EU544356	EU544316	EU544413	KF496311	EU544477
Anacolosa papuana	Australia		DQ790104	DQ790181	DQ790144	
Antidaphne viscoidea	America		L24080	EF464500	L26068	
Arjona tuberosa	America	EF464480	EF464468	EF464513	EF464532	EF464483
Atkinsonia ligustrina	Australia	EF464475	EF464464	DQ787444	EF464526	DQ788714
Bakerella sp.	Madagascar	EU544358	EU544318	EU544415	EU544466	EU544479
Baratranthus axanthus	IAA	EU544357	EU544317	EU544414		EU544478
Benthamina alyxifolia	Australia	EU544359	EU544319	EU544416	AY957440	EU544480
Berhautia senegalensis	Africa	EU544360	EU544320	EU544417		
Cathedra acuminata	America		FJ848847	DQ790182	DQ790145	
Cecarria obtusifolia	Australia	EU544361	EU544321	EU544418	EU544467	EU544481
Cladocolea gracilis	America	EU544362	EU544322	EU544419		EU544482
Dactyliophora novaeguineae	Australia	EU544363	EU544323	EU544420		EU544483
Decaisnina triflora	IAA	EU544364	EU544324	EU544421	EU544468	EU544484
Decaisnina aherniana	IAA	MG999386	MG999461	MG999410		
Dendropemon bicolor	America	EU544365	AF039075	EU544422	EU544469	

Dendrophthoe curvata	IAA	EU544367	EU544325	EU544424		
Dendrophthoe longituba	IAA	EU544366		EU544423		EU544485
Dendrophthoe gangliiformis	IAA		MG999462	MG999411	MG999431	MG999483
Dendrophthoe pentandra	Asia	MG999387	MG999463	MG999412	MG999432	MG999484
Desmaria mutabilis	America	EF464476	EF464465	EF464509	EF464527	EF464486
Diplatia furcata	Australia	EU544368	L24088	EU544425	KF496292	EU544486
Elytranthe albida	Asia	MG999388	MG999464	MG999413	MG999433	MG999485
Emelianthe panganensis	Africa	EU544369	EU544326	EU544426	MG999434	MG999486
Englerina ramulosa	Africa	EU544370	L24140	EU544427	EU544470	DQ340577
Englerina woodfordioides	Africa	MG999389	MG999465	MG999414	MG999435	MG999487
Erianthemum dregei	Africa	EU544371	L25679	EU544428		EU544488
Eubrachion ambiguum	America	AF389273	L24141	EF464498		
Gaiadendron punctatum	America	DQ790209	L24143	DQ787445	L26072	DQ340617
Globimetula dinklagei	Africa	EU544372	AF039076	EU544429		EU544489
Helicanthes elastica	India	EU544375	EU544328	EU544432		
Helixanthera parasitica	Asia	MG999391	MG999466		MG999437	MG999488
Helixanthera cylindrica	IAA	EU544374	EU544327	EU544431		
Helixanthera sampsonii	Asia			KP093921	KP094864	
Helixanthera coccinea	IAA	EU544373		EU544430		EU544490
Helixanthera kirkii	Africa	MG999390	AF039077		MG999436	
Ileostylus micranthus	New Zealand	EU544376	EU544329	EU544433	EU544471	EU544491
Lepeostegeres lanceifolius	IAA	EU544379		EU544435		
Lepidaria forbesii	IAA	EU544378	EU544330	EU544434		EU544492
Lepidoceras chilense	America		EF464459	EF464499	EF464519	
Ligaria cuneifolia	America	EF464477	L24152	EF464510	EF464528	DQ442940
Loranthus europaeus	Europe	EU544380	L24153	EU544436	JQ933393	EU544493
Loranthus odoratus	Asia	EU544381	EU544331			EU544494
Loxanthera speciosa	IAA	EU544382	EU544332	EU544437		EU544495
Lysiana filifolia	Australia	EU544383	EU544333	EU544438		EU544496
Macrosolen cochinchinensis	Asia IAA	EU544384	EU544334	EU544439	MG999439	MG999490
Macrosolen tricolor	IAA	MG999393	MG999468	MG999416	MG999440	MG999491
Macrosolen bibracteolatus	Asia	MG999392	MG999467	MG999415	MG999438	MG999489
Misodendrum linearifolium	America	DQ790211	L24397	DQ787438	L26074	DQ788712
Misodendrum punctulatum	America	KP263250	KP263284	DQ787443	EF464531	DQ788711
Moquiniella rubra	Africa	DQ790207	AF039078	DQ790171	DQ790132	EF464489
Muellerina eucalyptoides	Australia	EU544385	EU544335	EU544440	EU544472	EU544498
Notanthera heterophylla	America	EF464478	EF464466	EF464511	EF464529	DQ442939
Nuytsia floribunda	Australia	DQ790210	DQ790103	DQ787446	DQ790134	DQ788716
Oedina pendens	Africa	EU544386	EU544336	EU544441		EU544499
Oliverella rubroviridis	Africa	EU544387	EU544337	EU544442		
Oncella ambigua	Africa		EU544338	EU544443		
Oncocalyx sulfureus	Africa	EU544388	EU544339	EU544444	MG999442	EU544500
Oncocalyx fischeri	Africa	MG999394	MG999469	MG999417	MG999441	MG999492

Oryctanthus occidentalis	America	EU544389	L24408	EU544445		EU544501
Osyris alba	Europe	MG999395			MG999443	MG999493
Osyris lanceolata	Africa	MG999396	MG999470	MG999418	MG999444	MG999494
Passovia pyrifolia	America	EU544392	L24412	EU544448		EU544504
Peraxilla tetrapetala	New Zealand	EU544390	EU544340	EU544446	JQ933439	EU544502
Phanerodiscus capuronii	Madagascar	DQ790219	DQ790122	DQ790180	DQ790143	
Phragmanthera crassicaulis	Africa	EU544391	EU544341	EU544447		EU544503
Phragmanthera regularis	Africa	MG999397	MG999471	MG999419	MG999445	DQ340579
Plicosepalus sagittifolius	Africa	EU544393	EU544342	EU544449	MG999447	MG999496
Plicosepalus curviflorus	Africa	MG999398	MG999472	MG999420	MG999446	MG999495
Psittacanthus calyculatus	America	EU544394	L24414	EU544450		DQ340610
Quinchamalium chilense	America	KP263257	EF464469	EF464514	EF464533	EF464491
Santalum album	Asia	MG999399	MG999473	MG999421	MG999448	MG999497
Schoepfia fragrans	Asia	MG999400	MG999474	MG999422	MG999449	MG999498
Schoepfia jasminodora	Asia	MG999401	JQ613226	HQ415321	MG999450	AY191152
Schoepfia schreberi	America	AF389261	L24418	AY957454	L11205	DQ788717
Scurrula ferruginea	IAA	EU544395	EU544343	EU544451	KF114863	EU544505
Scurrula parasitica	Asia - IAA	EU544397	EU544345	EU544451	MG999454	MG999502
Scurrula pulverulenta	Asia	EU544396	EU544344	EU544452		
Scurrula chingii	Asia	MG999403	MG999476	MG999424	MG999452	MG999500
Scurrula chingii	Asia	MG999404	MG999477	MG999425	MG999453	MG999501
Scurrula philippensis	Asia	MG999405	MG999478	MG999426	MG999455	MG999503
Scurrula buddleioides	Asia	MG999402	MG999475	MG999423	MG999451	MG999499
Septulina glauca	Africa	EU544398	EU544346		AM235022	EU544506
Socratina bemarivensis	Madagascar	EU544399	EU544347	EU544454		EU544507
Sogerianthe sesailiflora	IAA	EU544400	EU544348	EU544455		EU544508
Spragueanella rhamnifolia	Africa			EU544456		
Struthanthus oerstedii	America	EU544402	L24421	EU544457	JQ594622	EU544509
Struthanthus woodsonii	America	EU544403	EU544349	EU544458	EU544474	EU544510
Tapinanthus constrictiflorus	Africa	EU544404	L24422	EU544459	EU213529	EU544511
Taxillus chinensis	IAA	EU544405	EU544350	EU544460	MG999456	MG999504
Taxillus thibetensis	Asia	MG999407	MG999480	MG999428	MG999458	MG999506
Taxillus tsaii	Asia	MG999408	MG999481	MG999429	MG999459	MG999507
Taxillus sutchuenensis	Asia	MG999406	MG999479	MG999427	MG999457	MG999505
Tolypanthus involucratus	Asia			EU544461		
Tripodanthus acutifolius	America	EU544406	L24424	EU544462	EU544475	EU544513
Tristerix corymbosus	America	EF464479	EF464467	EF464512	EF464530	DQ340605
Tupeia antarctica	New Zealand	DQ790208	L24425	DQ790172	DQ790133	EF464494
Vanwykia remota	Africa	EU544407	EU544351	EU544463		EU544514

23. Rubiaceae

Sample list was taken from the supplementary material of the original article (available at: https://onlinelibrary.wiley.com/action/downloadSupplement?doi=10.1111%2Fjbi.12981&file=jbi12981-sup-0001-AppendixS1.xls). In order to simplify the analysis, we chose the tribes that presented a diverse biogeographic distribution (Coffeae, Bertiereae, Octotropideae, and Pavetteae) and built the trees independently. In the alignments, we excluded the large gaps that represented apomorphic indels (of a single terminal). The calibration was made by extrapolating the dates obtained by the original article. For the Coffeae-Bertiereae analysis three calibration points were set: Bertiereae (lognormal distribution; M = 0.5 and S = 0.87; offset = 10.0 m.y.a.), Coffeae (lognormal distribution; M = 0.5 and S = 0.87; offset = 11.0 m.y.a.), and a root calibration (lognormal distribution; M = 0.5 and S = 0.95; offset = 15.0 m.y.a.). For the Octotropideae analysis three calibration points were set: root (lognormal distribution; M = 1.65 and S = 0.345; offset = 16.7 m.y.a.), *Didymosalpinx-Mantalania* (lognormal distribution; M = 1.74 and S = 0.33; offset = 14.0 m.y.a.), and *Burchellia-Polysphaeria* (lognormal distribution; M = 1.64 and S = 0.344; offset = 15.7 m.y.a.). For the Pavetteae-Sherbournieae analysis three calibration points were set: root (lognormal distribution; M = 1.48 and S = 0.34; offset = 16.4 m.y.a.), Pavetteae (lognormal distribution; M = 1.22 and S = 0.352; offset = 10.8 m.y.a.), and Sherbournieae (lognormal distribution; M = 1.73 and S = 0.35; offset = 9.97 m.y.a.).

References

Alström, P., Ericson, P. G. P., Olsson, U., & Sundberg, P. (2006). Phylogeny and classification of the avian superfamily Sylvioidea. *Molecular Phylogenetics and Evolution*, *38*(2), 381–397. doi:10.1016/j.ympev.2005.05.015

Arnold, E. N., & Poinar, G. (2008). A 100 million year old gecko with sophisticated adhesive toe pads, preserved in amber from Myanmar. *Zootaxa*, *1847*, 62–68. doi:10.5281/zenodo.274428.

Benton, M. J., & Donoghue, P. C. J. (2007). Paleontological Evidence to Date the Tree of Life. *Molecular Biology and Evolution*, *24*(1), 26–53. doi:10.1093/molbev/msl150

Benton, M. J., Donoghue, P. C. J., Asher, R. J., Friedman, M., Near, T. J., & Vinther, J. (2015). Constraints on the timescale of animal evolutionary history. *Palaeontologia Electronica*, *18*(1), 1–106. doi:https://doi.org/10.26879/424

Bernardes, S. C., Pepato, A. R., von Rintelen, T., von Rintelen, K., Page, T. J., Freitag, H., & de Bruyn, M. (2017). The complex evolutionary history and phylogeography of *Caridina typus* (Crustacea: Decapoda): long-distance dispersal and cryptic allopatric species. *Scientific Reports*, *7*(1), 9044. doi:10.1038/s41598-017-08494-w

Brattstrom, B. H. (1958). New records of Cenozoic amphibians and reptiles from California. *Bulletin of The Southern California Academy of Sciences*, *57*, 5–13.

Caldwell, M. W., & Albino, A. (2003). Exceptionally preserved skeletons of the cretaceous snake *Dinilysia patagonica* Woodward, 1901. *Journal of Vertebrate Paleontology*, *22*(4), 861–866.

Drummond, A. J., & Rambaut, A. (2007). BEAST: Bayesian evolutionary analysis by sampling trees. *BMC Evolutionary Biology*, *7*(1), 214. doi:10.1186/1471-2148-7-214

Ericson, P. G., Klopfstein, S., Irestedt, M., Nguyen, J. M., & Nylander, J. A. (2014). Dating the diversification of the major lineages of Passeriformes (Aves). *BMC Evolutionary Biology*, *14*(1), 8. doi:10.1186/1471-2148-14-8

Estes, R. (1976). Middle Paleocene lower vertebrates from the Tongue River Formation, Southeastern Montana. *Journal of Paleontology*, *50*(3), 500–520.

Feng, Y.-J., Blackburn, D. C., Liang, D., Hillis, D. M., Wake, D. B., Cannatella, D. C., & Zhang, P. (2017). Phylogenomics reveals rapid, simultaneous diversification of three major clades of Gondwanan frogs at the Cretaceous–Paleogene boundary. *Proceedings of the National Academy of Sciences*, *114*(29), E5864–E5870. doi:10.1073/pnas.1704632114

Godinot, M. (2006). Lemuriform origins as viewed from the fossil record. *Folia Primatologica*, *77*(6), 446–464. doi:10.1159/000095391

Gómez, R. O., Báez, A. M., & Rougier, G. W. (2008). An anilioid snake from the Upper Cretaceous of northern Patagonia. *Cretaceous Research*, *29*(3), 481–488. doi:10.1016/j.cretres.2008.01.002

Hartenberger, J.-L., & Marandat, B. (1992). A new genus and species of an early Eocene primate from North Africa. *Human Evolution*, *7*(1), 9–16. doi:10.1007/BF02437474

Heinicke, M. P., Daza, J. D., Greenbaum, E., Jackman, T. R., & Bauer, A. M. (2014). Phylogeny, taxonomy and biogeography of a circum-Indian Ocean clade of leaf-toed geckos (Reptilia: Gekkota), with a description of two new genera. *Systematics and Biodiversity*, *12*(1), 23–42. doi:10.1080/14772000.2013.877999

Herrera, J. P., & Dávalos, L. M. (2016). Phylogeny and divergence times of lemurs inferred with recent and ancient fossils in the tree. *Systematic Biology*, *65*(5), 772–791. doi:10.1093/sysbio/syw035

Hoffstetter, M. R. (1969). Un primate de l'Pliocène inférieur sudamericain: *Branisella boliviana* gen. et sp. nov. *Comptes Rendus Mathematique Academie des Sciences*, *269*, 434–437.

Jenkins, P. A., & Walsh, D. M. (1993). An Early Jurassic caecilian with limbs. *Nature*, *365*(6443), 246. doi:10.1038/365246a0

Joyce, W. G., Parham, J. F., Lyson, T. R., Warnock, R. C. M., & Donoghue, P. C. J. (2013). A divergence dating analysis of turtles using fossil calibrations: an example of best practices. *Journal of Paleontology*, *87*(4), 612–634. doi:10.1666/12-149

Laduke, T. C., Krause, D. W., Scanlon, J. D., & Kley, N. J. (2010). A Late Cretaceous (Maastrichtian) snake assemblage from the Maevarano Formation, Mahajanga Basin, Madagascar. *Journal of Vertebrate Paleontology*, *30*(1), 109–138. doi:10.1080/02724630903409188

Li, J.-T., Li, Y., Klaus, S., Rao, D.-Q., Hillis, D. M., & Zhang, Y.-P. (2013). Diversification of rhacophorid frogs provides evidence for accelerated faunal exchange between India and Eurasia during the Oligocene. *Proceedings of the National Academy of Sciences*, *110*(9), 3441–3446. doi:10.1073/pnas.1300881110

Maddin, H. C., Jenkins Jr, F. A., & Anderson, J. S. (2012). The braincase of *Eocaecilia micropodia* (Lissamphibia, Gymnophiona) and the origin of caecilians. *PLOS ONE, 7*(12), e50743. doi: 10.1371/journal.pone.0050743

MacPhee, R. D. E., & Jacobs, L. L. (1986). *Nycticeboides simpsoni* and the morphology, adaptations, and relationships of Miocene Siwalik Lorisidae. *Rocky Mountain Geology*, *24*(3), 131–161. doi:10.2113/gsrocky.24.special_paper_3.131

Marsh, O. C. (1892). Notice of new reptiles from the Laramie Formation. *American Journal of Science, Series 3 Vol. 43*(257), 449–453. doi:10.2475/ajs.s3-43.257.449

Masters, J. C., Silvestro, D., Génin, F., & DelPero, M. (2013). seeing the wood through the trees: the current state of higher systematics in the Strepsirhini. *Folia Primatologica*, *84*(3–5), 201–219. doi:10.1159/000353179

Miller, H. W. (1955). Some eocene reptiles from New Jersey. *The Academy of Natural Sciences of Philadelphia*, *Notulae Natura*, *268,* 1–5.

Onary, S., & Hsiou, A. S. (2018). Systematic revision of the early Miocene fossil *Pseudoepicrates* (Serpentes: Boidae): implications for the evolution and historical biogeography of the West Indian boid snakes (*Chilabothrus*). *Zoological Journal of the Linnean Society*, *184*(2), 453–470. doi:10.1093/zoolinnean/zly002

Porter, M. L., Pérez-Losada, M., & Crandall, K. A. (2005). Model-based multi-locus estimation of decapod phylogeny and divergence times. *Molecular Phylogenetics and Evolution*, *37*, 355–369. doi:10.1016/j.ympev.2005.06.021

Pyron, R. A. (2014). Biogeographic analysis reveals ancient continental vicariance and recent oceanic dispersal in amphibians. *Systematic Biology*, *63*(5), 779–797. doi:10.1093/sysbio/syu042

Rage, J.-C. (1984). The fossil snake *Cheilophis huerfanoensis* Gilmore, 1938, from Eocene of Colorado: redescription and reappraisal of relationships. *Journal of Vertebrate Paleontology*, *3*(4), 219–222. doi:10.1080/02724634.1984.10011977

Seiffert, E. R. (2006). Revised age estimates for the later Paleogene mammal faunas of Egypt and Oman. *Proceedings of the National Academy of Sciences*, *103*(13), 5000–5005. doi:10.1073/pnas.0600689103

Seiffert, E. R., Simons, E. L., & Attia, Y. (2003). Fossil evidence for an ancient divergence of lorises and galagos. *Nature*, *422*(6930), 421–424. doi:10.1038/nature01489

Seiffert, E. R., Simons, E. L., Ryan, T. M., & Attia, Y. (2005). Additional remains of Wadilemur elegans, a primitive stem galagid from the late Eocene of Egypt. *Proceedings of the National Academy of Sciences*, *102*(32), 11396–11401. doi:10.1073/pnas.0505310102

Simons, E. L. (1965). New fossil apes from Egypt and the initial differentiation of Hominoidea. *Nature*, *205*(4967), 135. doi:10.1038/205135a0

Simons, E. L. (1992). Diversity in the early tertiary anthropoidean radiation in Africa. *Proceedings of the National Academy of Sciences*, *89*(22), 10743–10747. doi:10.1073/pnas.89.22.10743

Simons, E. L. (1997). Discovery of the smallest Fayum Egyptian primates (Anchomomyini, Adapidae). *Proceedings of the National Academy of Sciences*, *94*(1), 180–184.

Sullivan, R. M., & Lucas, S. G. (1986). Annotated list of lower vertebrates from the Paleocene Nacimiento Formation (Puercan-Torrejonian), San Juan Basin, New Mexico. *Journal of Herpetology*, *20*(2), 202–209. doi:10.2307/1563945

Szyndlar, Z., Smith, R., & Rage, J.-C. (2008). A new dwarf boa (Serpentes, Booidea, 'Tropidophiidae') from the Early Oligocene of Belgium: a case of the isolation of Western European snake faunas. *Zoological Journal of the Linnean Society*, *152*(2), 393–406. doi:10.1111/j.1096-3642.2007.00357.x

Takai, M., Anaya, F., Shigehara, N., & Setoguchi, T. (2000). New fossil materials of the earliest new world monkey, *Branisella boliviana*, and the problem of platyrrhine origins. *American Journal of Physical Anthropology*, *111*(2), 263–281. doi:10.1002/(SICI)1096-8644(200002)111:2<263::AID-AJPA10>3.0.CO;2-6

von Rintelen, K., Page, T. J., Cai, Y., Roe, K., Stelbrink, B., Kuhajda, B. R., … von Rintelen, T. (2012). Drawn to the dark side: A molecular phylogeny of freshwater shrimps (Crustacea: Decapoda: Caridea: Atyidae) reveals frequent cave invasions and challenges current taxonomic hypotheses. *Molecular Phylogenetics and Evolution*, *63*(1), 82–96. doi:10.1016/j.ympev.2011.12.015

Wiens, J. J. (2011). Re-evolution of lost mandibular teeth in frogs after more than 200 million years, and re-evaluating Dollo's law. *Evolution*, *65*(5), 1283–1296. doi:10.1111/j.1558-5646.2011.01221.x
